# Development of Diagnostic Biomarkers for Detecting Diabetic Retinopathy at Early Stages Using Quantitative Proteomics

**DOI:** 10.1155/2016/6571976

**Published:** 2015-11-09

**Authors:** Jonghwa Jin, Hophil Min, Sang Jin Kim, Sohee Oh, Kyunggon Kim, Hyeong Gon Yu, Taesung Park, Youngsoo Kim

**Affiliations:** ^1^Department of Biomedical Sciences, Seoul National University College of Medicine, 28 Yongon-Dong, Seoul 110-799, Republic of Korea; ^2^Department of Biomedical Engineering, Seoul National University College of Medicine, 28 Yongon-Dong, Seoul 110-799, Republic of Korea; ^3^Department of Ophthalmology, Samsung Medical Center, Sungkyunkwan University School of Medicine, Seoul 135-710, Republic of Korea; ^4^Department of Biostatistics, Seoul Metropolitan Government-Seoul National University Boramae Medical Center, 20 Borame-ro 5-gil, Dongjak-gu, Seoul 156-707, Republic of Korea; ^5^Department of Ophthalmology, Seoul National University College of Medicine, 28 Yongon-Dong, Seoul 110-799, Republic of Korea; ^6^Department of Statistics, Seoul National University, 1 Gwanak-ro, Gwanak-gu, Seoul 151-747, Republic of Korea

## Abstract

Diabetic retinopathy (DR) is a common microvascular complication caused by diabetes mellitus (DM) and is a leading cause of vision impairment and loss among adults. Here, we performed a comprehensive proteomic analysis to discover biomarkers for DR. First, to identify biomarker candidates that are specifically expressed in human vitreous, we performed data-mining on both previously published DR-related studies and our experimental data; 96 proteins were then selected. To confirm and validate the selected biomarker candidates, candidates were selected, confirmed, and validated using plasma from diabetic patients without DR (No DR) and diabetics with mild or moderate nonproliferative diabetic retinopathy (Mi or Mo NPDR) using semiquantitative multiple reaction monitoring (SQ-MRM) and stable-isotope dilution multiple reaction monitoring (SID-MRM). Additionally, we performed a multiplex assay using 15 biomarker candidates identified in the SID-MRM analysis, which resulted in merged AUC values of 0.99 (No DR versus Mo NPDR) and 0.93 (No DR versus Mi and Mo NPDR). Although further validation with a larger sample size is needed, the 4-protein marker panel (APO4, C7, CLU, and ITIH2) could represent a useful multibiomarker model for detecting the early stages of DR.

## 1. Introduction

Diabetic retinopathy (DR) is a common microvascular complication caused by diabetes mellitus (DM) and is a leading cause of vision impairment and loss among adults [1]. It ultimately affects more than 90% of diabetic patients to some degree. Approximately 10% of patients with diabetes for ≥15 years develop severe visual impairment, and about 2% of these patients become blind [2, 3]. Vision impairment associated with DR can be further categorized into nonproliferative diabetic retinopathy (NPDR) and proliferative diabetic retinopathy (PDR); NPDR causes central vision loss when it induces diabetic macular edema (DME) [4].

PDR is the more advanced stage of DR and is characterized by retinal neovascularization. The manifestations of PDR include vitreous hemorrhage, formation of fibrous periretinal tissue accompanying neovascularization, traction retinal detachment, and, finally, total vision loss [5, 6]. Currently, the main treatments for DR are retinal photocoagulation and vitreoretinal surgery; there are no specific drugs currently available to prevent or slow down DR. Although the control of systemic risk factors, such as hyperglycemia, hypertension, and dyslipidemia [7], has been shown to improve clinical therapies for diabetes-induced vision loss, more effective clinical therapies for DR patients are needed.

Recently, biomarker discovery has become important in many aspects of disease diagnosis and drug discovery and development [8–10]. Predictive biomarkers can save time and money and lead to better diagnoses, ultimately aiding the cure of diseases. Indeed, many biomarkers have an important diagnostic role in the detection or prediction of disease. However, convenient biomarkers must be detectable in tests of blood, urine, or saliva, which can be performed at the bedside or in an outpatient setting [11–13]. Although urinary and tissue samples have been primarily used in the past to identify biomarkers, plasma and serum can also be collected noninvasively and contain factors that can be more effective in biomarker development. Indeed, blood is routinely collected and contains thousands of proteins, including secreted proteins and proteins shed into the blood by tumors [9, 14–16].

The pipeline for biomarker development usually includes 4 steps—discovery, verification, validation, and product development. Clinical verification or validation is well known to be the biggest barrier for biomarker development. To overcome this barrier, effective methods to facilitate large-scale biomarker validation are needed. Immunological methods and multiple reaction monitoring (MRM) are being considered as possible solutions. Between these 2 methods, the MRM approach has a much higher throughput and accuracy and allows for substantial multiplexing [17]. With the use of MRM analysis, more than 100 candidate proteins can be simultaneously targeted and measured at the clinical verification stage.

There have been many proteomic studies on DR that analyze the retina, vitreous, or plasma, which have led to a better understanding of the DR proteome [17–19]. Based on these studies, we decided to use a systemic approach to identify biomarkers for DR-related diseases.

First, to obtain biomarker candidates that are specifically expressed in the tissues of macula hole (MH, nondiabetic controls), NPDR, and PDR patients, we performed data-mining on the previously published DR-related studies [6, 18–25] and our experimental data set in human vitreous. This proteomic information provided baseline data, including 96 possible DR biomarker candidates. Second, the 96 selected candidates were used to carry out a semiquantitative multiple reaction monitoring (SQ-MRM) verification experiment, in which we used plasma samples from type 2 diabetes patients without DR (No DR), with mild nonproliferative DR (Mi NPDR), and with moderate nonproliferative DR (Mo NPDR). We were able to select 15 candidates by SQ-MRM verification and, thereafter, subsequent stable-isotope dilution multiple reaction monitoring (SID-MRM) verification narrowed these candidates down to 11 potential markers. Third, we constructed a model for a multimarker panel using these 11 verified markers and performed leave-one-out cross validation (LOOCV) on the panel to avoid overfitting and to calculate error rates. For this statistical evaluation, we used linear discriminant analysis (LDA) methods.

Consequently, this systemic approach allowed us to select optimal biomarker candidates for inclusion in a multimarker panel. We determined that a 4-marker panel was a better diagnostic tool for detecting early grade NPDR and was associated with lower error rates than the use of the single best marker alone.

## 2. Materials and Methods

### 2.1. Plasma Sample Preparation

To verify DR biomarker candidates, we collected plasma from 20 diabetic patients with no diabetic retinopathy (No DR), mild nonproliferative diabetic retinopathy (Mi NPDR), or moderate nonproliferative diabetic retinopathy (Mo NPDR). DR was classified by the international clinical diabetic retinopathy disease severity scale [26]. Each plasma sample was collected in 10 mL tubes containing K_2_-EDTA. Tubes were centrifuged at 3000 ×g for 10 min at 4°C, and 100 *μ*L was aliquoted into new tubes and stored at −70°C.

All patients provided informed consent before being enrolled in this study, in accordance with the protocol approved by the Institutional Review Board at Seoul National University Hospital (IRB number H-0807-086-251).

### 2.2. MARS Depletion

To deplete plasma using a MARS column, plasma was diluted 5-fold with MARS buffer A (1 : 4) and filtered with a 0.22 *μ*m Ultrafree-MC Durapore centrifugal filter (Cat. number UFC30GVNB, Millipore, Billerica, MA, USA). Each plasma sample was applied to a MARS column on an LC-10AT HPLC system (Shimadzu, Kyoto, Japan). The sample loop volume of the HPLC was set to 200 *μ*L, and 200 *μ*L of 5-fold diluted plasma was injected into the MARS column. The total LC run time of 37 min involved the following: 100% MARS buffer A at a flow rate of 0.7 mL/min for the first 11 min, sample injection, wash for 11 min, 100% MARS buffer B at a flow rate of 1.0 mL/min for 5 min, and 100% MARS buffer A at a flow rate of 0.7 mL/min for 10 min.

The UV detector was set to 280 nm for plasma injection, and the eluted fractions were collected in 250 *μ*L aliquots. The flow-through and bound fractions were eluted in 10 fractions (total, 2.5 mL), and the eluted fractions were pooled for MRM.

### 2.3. Preparation of Plasma for Mass Spectrometry

The protein concentration in the vitreous and depleted plasma was measured using BCA methods according to the protocol provided by the manufacturer. Aliquots of 100 *μ*g protein were reduced, alkylated, and digested according to the protocol. Briefly, 60 *μ*L 9 M urea (5.4054 g) and 30 mM dithiothreitol (DTT; 0.04628 g) in 10 mL 100 mM Tris-base (pH 8.0) were added to each sample and incubated for 30 min at 37°C. Each sample was allowed to cool at room temperature before 9 *μ*L 500 mM iodoacetamide (IAA; 0.0925 g/mL) was added. The solution was incubated for 20 min at room temperature. To dilute the urea from 6 M to 0.6 M, 771 *μ*L 100 mM Tris buffer (pH 8.0) was added to each sample. Each sample was then digested with trypsin (Promega, Madison, WI, USA) at a protein-to-enzyme ratio of 50 : 1 at 37°C overnight. To quench the digestion reaction, 50 *μ*L 0.1% TFA was added. The digested peptide mixture was applied onto an HLB Oasis cartridge (Waters Milford, MA, USA) for desalting, and peptides were eluted using 1 mL 60% ACN with 0.1% FA solution.

### 2.4. Determination of Transitions Using the Skyline Program

To determine the optimal MRM transition, including precursor (Q1) and fragment (Q3) ions, we used an* in silico* method utilizing the Skyline program (version 2.1), which is an open-source software application for developing MRM methods and analyzing MRM data [27]. The FASTA file for each protein selected by data-mining was imported into the Skyline program; the precursor and fragment ions for each protein were generated by performing* in silico* digestion. Our peptide filter conditions were as follows: the precursor length range was set at 8 to 20 amino acids, and peptides with repeat arginine (Arg, R) or lysine (Lys, K) residues were not used. To avoid using peptides that included potentially modified forms in the sequence, methionine (Met, M) and cysteine (Cys, C) residues were discarded. If proline (Pro) was next to an arginine (Arg, R) or lysine (Lys, K) residue, the peptide was not used. Peptides containing histidine (His, H) were also discarded because of the possibility of alterations in the side chain charge.

### 2.5. Multiple Reaction Monitoring Using Triple Quadrupole Mass Spectrometry

Our semiquantitative multiple reaction monitoring (SQ-MRM) and stable-isotope dilution multiple reaction monitoring (SID-MRM) analyses were performed using a triple quadrupole linear ion trap mass spectrometer (a 4000 QTRAP coupled with a nano-Tempo MDLC, Applied Biosystems, Carlsbad, CA, USA), as described in a previous study [28]. To reduce the void volume and obtain sharper intensity peaks, we used a modified sample loop (100 *μ*m ID capillary tubing containing 1.0 *μ*L sample) in an autosampler. In our MRM analysis, a 15 cm homemade analytical column was used—an IntegraFrit capillary (ID, 75 *μ*m; OD, 360 *μ*m) was packed with Magic C18AQ (200 Å, Michrom Bioresources, Madison, WI, USA). We directly injected 1 *μ*L of our prepared sample with Sol A (98% DW, 2% ACN, and 0.1% FA) and Sol B (98% ACN, 2% DW, and 0.1% FA) into the analytical column without a trap column; the flow rate was set to 300 nL/min. Exponential gradient elution (60 min) was performed by increasing the mobile phase composition from 0 to 40% Sol B. The gradient was then increased to 90% B for 10 min and 0% B for 10 min to equilibrate the column for the next run. The optimal parameters for a triple quadrupole mass spectrometer that interfaced with a nanospray source were set as follows: ion spray (IS), 2100 V; source temperature, 160°C; high collision gas, approximately 4-3 × 10^−5^ torr; and curtain gas, 15. MS parameters for declustering potential (DP) and collision energy (CE) were determined using the Skyline program. In the MRM run, the scan time for each transition and the pause time between transition scans were set to 15 ms and 5 ms, respectively. MRM analysis was composed of 2 steps: the first MRM step determined the transition and the second step monitored the target transition for quantification. In the first MRM step, an enhanced mass scan (EMS) ranging from 400 to 1200* m/z* (scan speed, 4000 Da/s) was performed, and an enhanced product ion (EPI) scan was then carried out to obtain MS/MS spectra. A MASCOT search was performed to identify the protein. In the second MRM step, an MRM analysis was performed to quantify the selected target transition without any information-dependent scan or MS/MS scan (see the Supplementary Methods for more details in Supplementary Material available online at http://dx.doi.org/10.1155/2016/6571976). In the SQ-MRM analysis, we spiked each sample with 50 fmol betaGAL (GDFQFNISR, 542.3/636.3* m/z*) during our preparation of the digested sample. In the SID-MRM analysis, 15 synthetic heavy peptides (>95% purity) were used.

### 2.6. Synthetic Peptides

For the SID-MRM analysis, we first synthesized stable-isotope standard (SIS) peptides (>95% purity) for 15 proteins (APLP2, APOA4, APOH, B3GNT1, C4B, C5, C7, CD14, CLU, FN1, GSN, ITIH2, KRT1, SERPINF1, and VTN). Synthetic peptides were obtained from 21st Century Biochemicals (Marlboro, MA, USA). Peptide sequences were synthesized as unmodified peptides with free N- and C-terminal amino acids. The purity of the synthetic peptides was >95%, as measured by HPLC. For stable isotope-labeled peptides, the C-terminal arginine or lysine contained ^13^C- and ^15^N-labeled atoms. Peptide stock concentrations were determined by amino acid analysis (AAA).

### 2.7. Determination of the Lowest Limit of Detection (LLOD) and the Lowest Limit of Quantification (LLOQ)

To determine the LLOD and LLOQ, we used the method described by Keshishian et al. and other authors [29–31]. The LLOD was calculated based on the variance of the blank sample (sample b, with no “spiked” in analyte) and standard deviation (S.D.) of the sample with the lowest concentration among the “spiked” in samples (0.1 fmol/*μ*L). Equation (1) was used to determine LLOD as follows:

(1) $$\begin{array}{c} LLOD=LOB+c_{\beta }\times S.D.s. \end{array}$$

For this equation, if the analyte is detected when it is not present and if the analyte is not detected when it is present, the type I error rate *α* and the type II error rate *β* are 0.05 and 0.05, respectively. The limit of the blank (LOB) was defined as the 95th percentile of the blank sample. For a relatively small number of repeated measurements of the blank, the *c*_*β*_ was approximated as follows: *c*_*β*_ = *t*_1−*β*_; thus,

(2) $$\begin{array}{c} LLOD={mean}_{b}+\frac{t_{1-\beta }\left(S.D._{b}+S.D.s\right)}{\sqrt{n}}. \end{array}$$

For this equation, *t*_1−*β*_ is equal to the (1 − *β*) percentile of the standard *t* distribution with *n* degrees of freedom; *n* is equal to the number of replicates. Consequently, the LLOD equation can be expressed as in the following equation:

(3) $$\begin{array}{c} LLOD={mean}_{b}+\frac{t0.95\left(S.D._{b}+S.D.s\right)}{\sqrt{n}}. \end{array}$$

The LLOQ was calculated using the following standard calculation [29–31]: LLOD × 3 = LLOQ.

### 2.8. Statistical Analyses

To enhance the classification power of the biomarkers, we constructed a multimarker panel using individual biomarkers and then performed a statistical evaluation of the panel. Using the SPSS Statistics software program (IBM Inc., Armonk, NY, USA, ver. 20), we first conducted a correlation analysis to examine the variable stability by Pearson's correlation analysis. Second, Student's* t*-test and stepwise multivariate analysis of variance (MANOVA) analysis [32] were performed to select the multimarker proteins that contributed to the discriminatory power between the No DR and Mo NPDR groups. Finally, to validate the multimarker models from multivariate analysis, LOOCV was conducted to avoid overfitting and to calculate the error rate of the model by comparing the No DR and Mo NPDR groups. To validate the multimarker panel, we used the linear discriminant analysis (LDA) method. We used the MedCalc (MedCalc Software, Mariakerke, Belgium) program to perform the ANOVA and chi-square tests and to generate receiver operating characteristic (ROC) curves.

### 2.9. Gene Ontology (GO) and Functional Analysis

To analyze the GO terms in the protein data sets, we used the DAVID bioinformatics resource (http://david.abcc.ncifcrf.gov/), which allows both the functional classification and the ID conversion of the proteins that we identified. The “biological process” and “molecular function” classifications were analyzed using PANTHER ID numbers (http://www.pantherdb.org/).

## 3. Results

### 3.1. Plasma Characteristics of the No DR, Mi NPDR, and Mo NPDR Groups

We collected 20 plasma samples with a 1 : 1 gender ratio from each of the No DR, Mi NPDR, and Mo NPDR patient groups. The patient gender, patient age, DM status, HbAlc, and plasma concentration for these 60 plasma samples are shown in Supplementary Table  1. We sorted patients that had no complications from diabetes except diabetic retinopathy and, thus, all patients in this study have no kidney diseases like diabetic nephropathy.

To examine whether any other factors could interfere with the MRM analysis, we carried out ANOVA tests for age, DM duration, and HbAlc and used chi-square tests for gender and hypertension (Supplementary Table  2). We detected no significant differences related to gender, age, hypertension, or HbAlc. For DM duration, increased DM duration was observed in the NPDR group compared to the No DR group. There were also differences in DM duration between the No DR and Mo NPDR groups, as indicated by the ANOVA findings (F-statistics).

### 3.2. Our Overall Scheme for DR Biomarker Development

**Figure 1:**
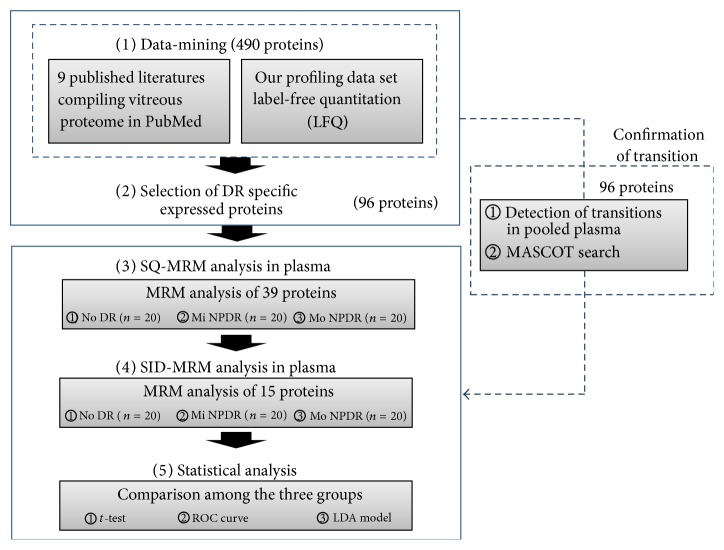
The overall scheme for DR biomarker development. The first step to develop biomarkers is to identify candidates. To that end, we performed a comprehensive proteomics study of DR samples, which included 2 steps: (1) discovering and (2) validating biomarker candidates. In the discovery stage, 96 proteins were selected as DR-specific proteins in human vitreous. Using a MASCOT search, 39 proteins were confirmed in human plasma. In the verification stage, SQ-MRM and SID-MRM analyses were performed to verify the candidates. Finally, statistical analysis was performed on the 15 verified candidates.

The first step in biomarker development is to identify candidates. To this end, we performed a comprehensive proteomics study of DR samples, which included 2 steps: (1) discovery of biomarker candidates in vitreous samples and (2) verification of biomarker candidates in plasma samples (Figure 1).

First, we performed data-mining on previously published DR-related studies and our experimental data from human vitreous samples. In the candidate discovery stage, 96 proteins were selected from literatures and our experimental data (Supplementary Tables  3–5). The 1870 transitions for these 96 proteins were generated using the Skyline program and then were confirmed by MRM analysis, where 39 proteins were confirmed (Supplementary Table  6). The 39 candidates were then examined by SQ-MRM analysis, where the expression of 15 proteins significantly differed between the No DR and Mi NPDR or Mo NPDR groups with an AUC > 0.7. Furthermore, the 15 candidate proteins were also verified by SID-MRM analysis using the same plasma samples.

For the final stage of biomarker development, models were constructed for multimarker panels composed of protein variables having discriminatory power. First, we checked for correlation and multicollinearity among the 15 potential markers to evaluate the discrimination power. Next, using Student's* t*-test and stepwise MANOVA analyses, we selected 4 potential markers with an expression pattern that differed significantly between the No DR and Mo NPDR groups. Using the 4 differentially expressed marker proteins, a multimarker panel was constructed and statistical validation was performed using LOOCV. To examine the efficacy of the model, the LDA method was used for statistical analysis (Figure 1).

### 3.3. Selection of Biomarker Candidates

We carried out data-mining on all previously published DR-related studies in humans [6, 18–25] (Supplementary Table  5). For standardization and integration of protein names, all accession number formats, such as Uniprot, IPI, GI number, and GenBank number, were converted into gene symbols. Protein data, such as identification and quantification in a DR-related study, were extracted; data were compiled from 9 DR proteomics studies that reported quantitative data, and a total of 465 proteins were collected by data-mining. Additionally, we also collected LC-ESI-MS/MS experimental data, 136 differentially expressed proteins. In the data-mining stage, 490 proteins were ultimately identified. To select the most reliable candidates, proteins found in more than 4 hits in 10 datasets were selected (our experimental data set had “2 weighted hits”). Consequently, 96 proteins were finally selected as candidates and 1870 transitions were generated for these proteins using the Skyline program.

For MRM analysis, we first optimized the collision energy (CE) and declustering potential (DP) using the Skyline parameter optimization module. We then confirmed whether the 1870 transitions could be detected in pooled plasma and also tested whether these transitions could be identified as target proteins using a MASCOT search. From MASCOT searches, 39 proteins were ultimately confirmed, for which 22 proteins had 2 peptides and 17 proteins had 1 peptide (Supplementary Table  6).

### 3.4. Confirmation of Biomarker Candidates by SQ-MRM Analysis

**Figure 2:**
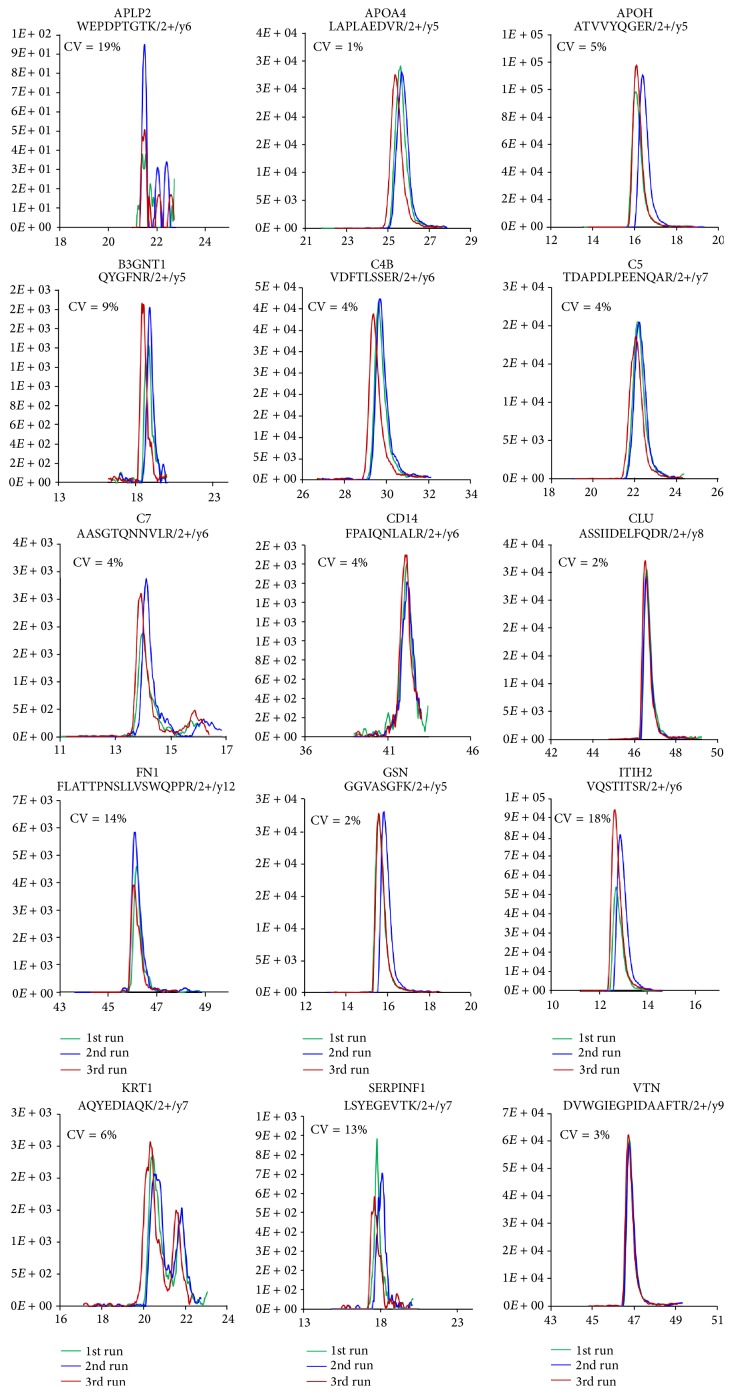
The reproducibility of the 3 independent MRM analyses. Before performing the SQ-MRM analysis, we first determined the reproducibility in 3 independent experiments using pooled plasma samples; representative results of 15 proteins are shown. The reproducibility testing showed that APLP2, FN1, ITIH2, and SERPINF1 had higher CV values (19%, 14%, 18%, and 13%, resp.), while the other proteins had lower CV values.

Before performing MRM analysis, we first determined the MRM reproducibility in 3 replicate experiments using pooled plasma samples for the detection of 15 representative proteins (Figure 2). We found that APLP2, FN1, ITIH2, and SERPINF1 had higher CV values (19%, 14%, 18%, and 13%, resp.), while the other proteins had lower CV values (Figure 2). However, the CV values for all of the proteins were less than 20%, even though we did not use an internal standard for normalization.

To obtain a stable quantitative peak area in the SQ-MRM analysis, we spiked 50 fmol betaGAL (GDFQFNISR, 542.3/636.3* m/z*) into the individual digested samples. To verify the selected biomarker candidates, we measured the relative concentration of the 39 candidates in the No DR, Mi NPDR, and Mo NPDR groups. Using MRM analysis, we confirmed differential measurements of these proteins among the No DR (*n* = 20), Mi NPDR (*n* = 20), and Mo NPDR (*n* = 20) groups.

**Figure 3:**
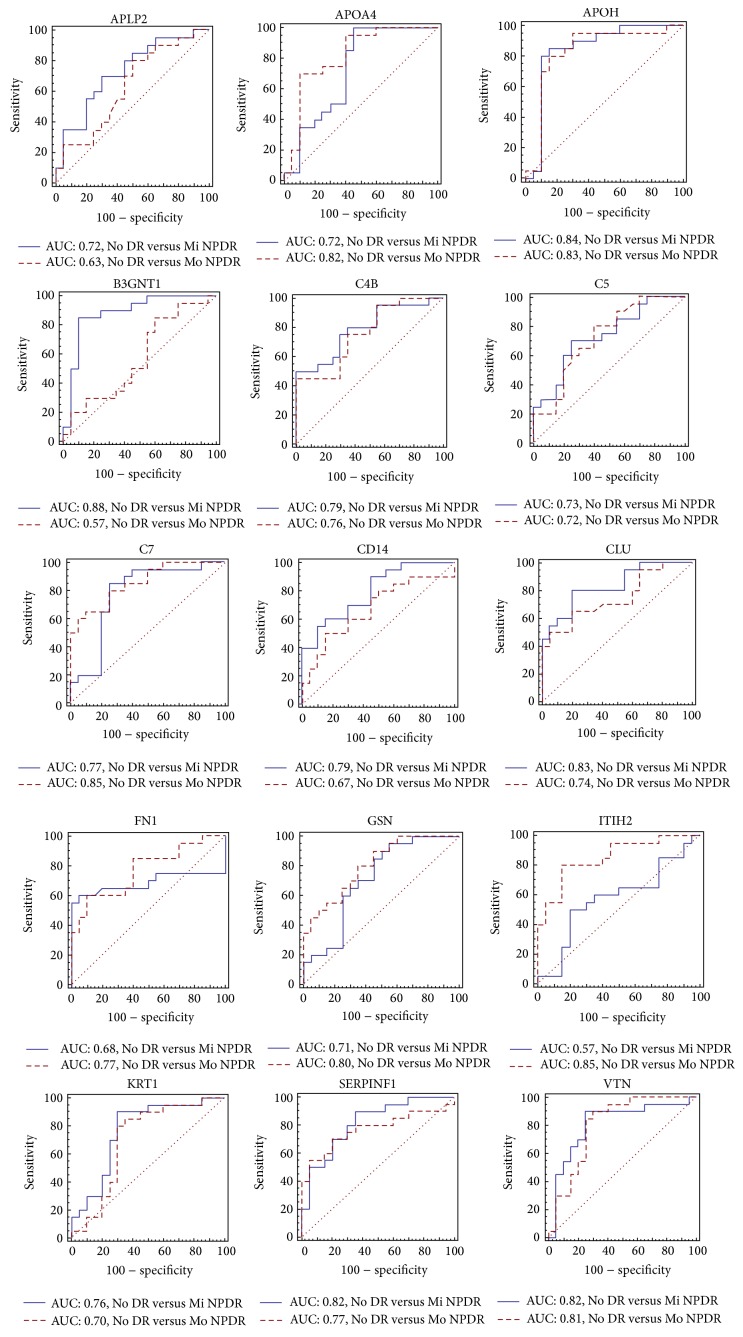
Verification of biomarker candidates using SQ-MRM analysis. To verify the selected biomarker candidates, we measured the relative concentrations of the 39 candidates in the samples from the No DR, Mi NPDR, and Mo NPDR groups. In the SQ-MRM analysis, we confirmed a differential measurement of these proteins among the No DR (*n* = 20), Mi NPDR (*n* = 20), and Mo NPDR (*n* = 20) groups. ROC with AUC values were generated based on the SQ-MRM analysis. In the SQ-MRM analysis, we “spiked” the samples with 50 fmol betaGAL (GDFQFNISR, 542.3/636.3* m/z*).

In our quantification analysis (Figure 3), 15 proteins (amyloid-like protein 2, APLP2; apolipoprotein A-IV, APOA4; beta-2-glycoprotein 1, APOH; N-acetyllactosaminide beta-1,3-N-acetylglucosaminyltransferase, B3GNT1; complements C4-B, C4B; complements C5, C5; complement components C7, C7; monocyte differentiation antigens CD14, CD14; clusterin, CLU; fibronectin, FN1; gelsolin, GSN; inter-alpha-trypsin inhibitor heavy chain H2, ITIH2; keratin type II cytoskeletal 1, KRT1; pigment epithelium-derived factor, SERPINF1; and vitronectin, VTN) were significantly differentially expressed either in the No DR versus Mi NPDR (AUC value > 0.7) or in the No DR versus Mo NPDR (AUC value > 0.7) comparisons. Notably, B3GNT1 (0.88) and both C7 and ITIH2 (0.85) showed the highest AUC values in the No DR versus Mi NPDR and the No DR versus Mo NPDR comparisons, respectively. We performed MRM verification using SID-MRM on the 15 selected candidate proteins associated with an AUC value > 0.7.

### 3.5. Functional Analysis of the 15 Verified Proteins

To examine the biological functions of the 15 proteins from the SQ-MRM analysis, proteins were assigned biological process and molecular function categories using the PANTHER classification program. The largest assigned biological process and molecular function category were “immunity and defense” (33% and 20%, resp.) (Supplementary Figure  1A). Moreover, 5 proteins (APOH, C4B, C5, C7, and CD14), which are involved in biological process subcategories of “immunity and defense,” all showed a downregulated pattern (100%) when the No DR group was compared to the Mo NPDR group. Two proteins (APLP2 and KRT1), which are related to the biological process subcategory “developmental process,” also showed a downregulated pattern (100%) compared to the No DR and Mo NPDR groups.

To investigate the signaling pathways related to the 15 verified proteins, we conducted a signaling pathway analysis. We identified 2 signaling pathways (the actin cytoskeleton and complement cascade pathways) associated with these proteins in the KEGG database (Supplementary Figures  1B and C). CD14, FN1, and GSN play roles in the actin cytoskeleton pathway; CD14 and GSN were downregulated, whereas FN1 was upregulated. C4B, C5, and C7 play roles in the complement cascade pathway; all 3 proteins were downregulated.

### 3.6. The Verification of the 15 Candidate Proteins Using SID-MRM Analysis

Before we synthesized the SIS heavy peptides for SID-MRM analysis, we first examined how many peptides we should synthesize to obtain the reliable quantitated data. As a preliminary SQ-MRM experiment, we analyzed the correlation of peak area values between two peptides for the 22 proteins with 2 peptides and, then, the peak similarities of two peptides were analyzed by Pearson's correlation analysis. The patterns of peak intensity were highly correlated between two peptides in each protein showing the median value of 0.927 (Supplementary Figure  2). Consequently, the peptide, which had the highest intensity (best detectability) of the multiple peptides per protein, was finally selected and synthesized for SID-MRM analysis (>95% purity).

First, the purity of the SIS heavy peptides for SID-MRM was confirmed by MALDI-TOF analysis (Table 1 and Supplementary Figure  3). All of the SIS heavy peptides had equivalent theoretical and experimental molecular weights. For accurate confirmation of spectral similarity between the experimental spectra obtained in our MRM analysis and the spectra derived from a DR spectral library, we constructed a DR-specific MS/MS spectral library (MS/MS spectra for 3067 peptides); to generate DR-specific MS/MS spectra, a DR sample, which consisted of pooled plasma (1 *μ*g/*μ*L) and the 15 SIS peptides mixture (50 fmol/*μ*L), was analyzed by Q-Exactive quadrupole-Orbitrap mass spectrometry (Supplementary Methods).

**Figure 4:**
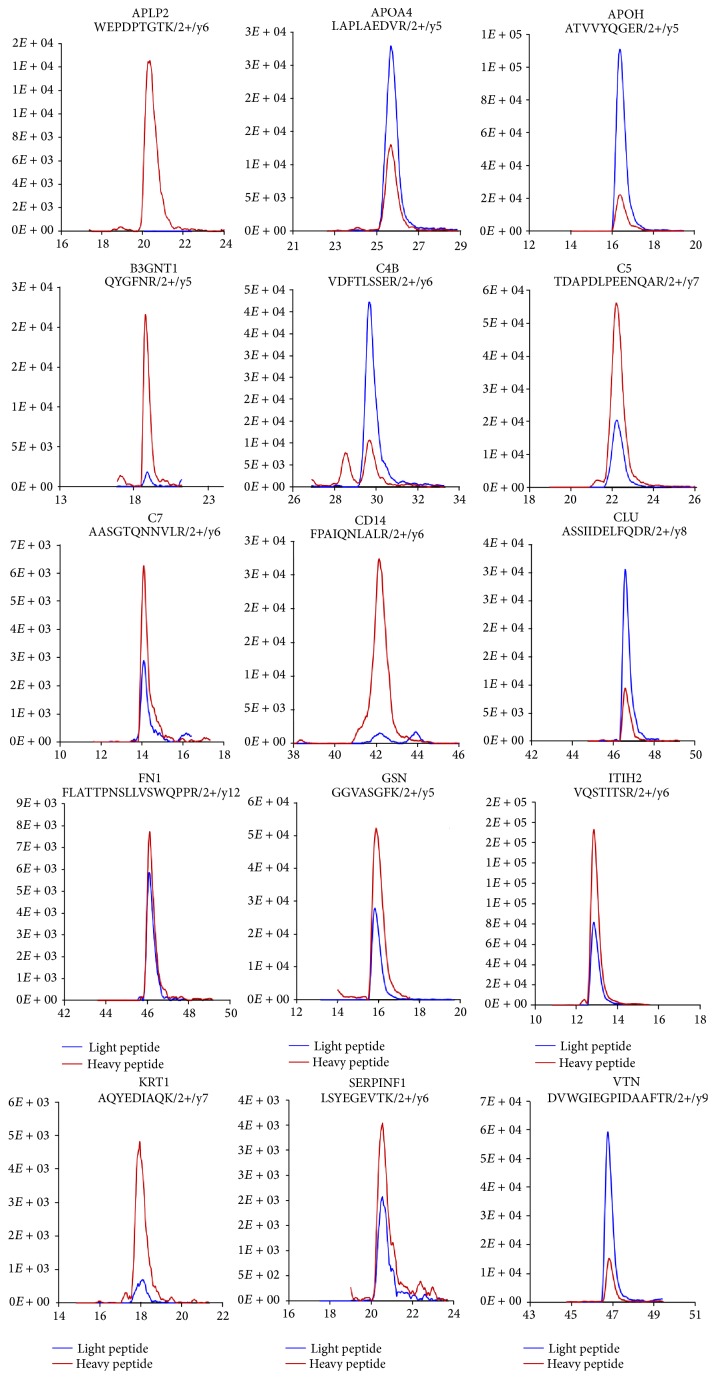
Detection of endogenous light peptide levels in pooled plasma. The endogenous light peptide levels in pooled plasma were measured by analyzing TEST sample #1, which was composed of endogenous light peptides (pooled digested plasma, 1 *μ*g/*μ*L) and heavy peptides (50 fmol of the heavy peptide mixture). From this analysis, low (APLP2, B3GNT1, CD14, and KRT1), middle (C5, C7, FN1, GSN, ITIH2, and SERPINF1), and high (APOA4, APOH, C4B, CLU, and VTN) concentration ranges of proteins were confirmed.

**Figure 5:**
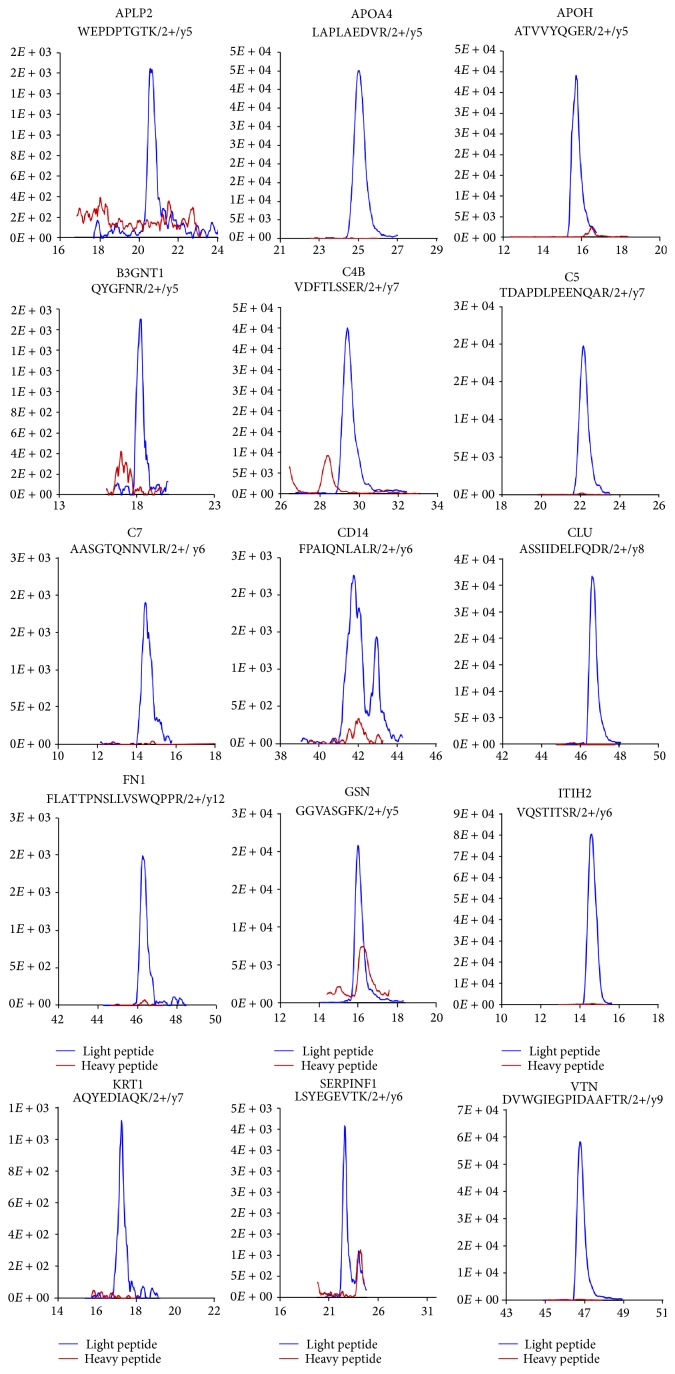
The detection of noise signals for heavy peptides in the pooled plasma. Noise signals of heavy peptides were assessed by analyzing TEST sample #2, which was composed of only endogenous light peptides (pooled plasma, 1 *μ*g without heavy peptide). A range of noise signals was detected for APLP2, B3GNT1, CD14, and GSN, while a noise signal was barely detected for the other proteins.

Prior to performing a second round of MRM verification of the 15 selected candidates, we first checked endogenous light peptide levels by analyzing TEST sample #1, which was composed of endogenous light peptides (pooled digested plasma, 1 *μ*g/*μ*L) and heavy peptides (50 fmol of the 15 heavy peptides mixture). We were able to confirm low (APLP2, B3GNT1, CD14, and KRT1), middle (C5, C7, FN1, GSN, ITIH2, and SERPINF1), and high (APOA4, APOH, C4B, CLU, and VTN) endogenous concentrations of the proteins, wherein the ranges of low, middle, and high concentrations were defined by comparing endogenous peptide with heavy peptide concentrations (<10 fmol, <50 fmol, and >50 fmol, resp.) (Figure 4). Second, we checked the noise signal of the heavy peptide level by analyzing TEST sample #2, which was composed of only endogenous light peptides (pooled plasma, 1 *μ*g without heavy peptide). We found that the detected signal at the heavy peptide position represented noise when we performed MRM analysis using the individual plasma samples (endogenous light peptide). A range of noise signals were detected for APLP2, B3GNT1, CD14, and GSN, while noise signals were barely detected for the other proteins (Figure 5).

To verify the biomarker candidates using SID-MRM analysis, we evaluated the linearity of the 15 proteins by analyzing a standard curve generated by serial dilutions of a known concentration of a heavy peptide. Next, we determined the LLOD and LLOQ for the 15 selected biomarker candidates. The LLOD was calculated based on the variance of the blank sample and standard deviation (S.D.) of the sample with the lowest level of “spiked” in SIS peptides (see Section 2 for details). Finally, using the heavy peptide mixture as an internal standard, individual plasma concentrations were measured using SID-MRM.

**Figure 6:**
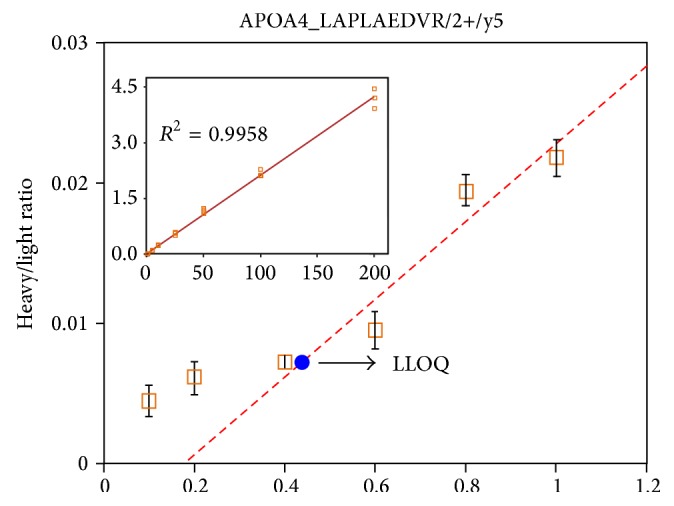
Representative calibration curves. To generate calibration curves for the 15 proteins, heavy peptides were serially diluted (12 concentrations, 0.1–200 fmol) with the light peptide as an internal standard (digested pooled plasma protein, 1 *μ*g) that was added into each serially diluted sample. A representative calibration curve for APOA4_LAPLAEDVR (y2^++^) is shown. Insets show the linear ranges of the curves using *R* ^2^ ≥ 0.99 to determine the LLOQ.

To generate a calibration curve for the 15 proteins, heavy peptides were serially diluted to yield 12 different concentrations (0.1–200 fmol) of the light peptide (digested pooled plasma protein, 1 *μ*g). Each experiment was repeated in triplicate to plot the calibration curve. The area ratio of heavy peptide (12 concentrations from 0.1–200 fmol) to light peptide (internal standard, 1 *μ*g plasma) was determined from the extracted ion chromatogram (XIC) of each selected transition. These calibration curves demonstrated linearity of approximately 3 orders of magnitude for the concentration range, and a strong linear correlation (*R* ^2^ > 0.98) was observed for 13 of the 15 calibration curves, in which APLP2 and B3GNT1 showed lower *R* ^2^ values of 0.96 and 0.94, respectively (Supplementary Figures  4A and 4B and Table  2).  Figure 6 shows the representative calibration curve for the APOA4 peptide (LAPLAEDVR_2^++^).

To determine the concentration range of quantification for each peptide, we determined the LLOD and LLOQ for each of the 15 proteins. The LLOD and LLOQ for 4 proteins (APOA4, CLU, ITIH2, and VTN) were very low (0.06–0.32 fmol/*μ*L and 0.19–0.96 fmol/*μ*L, resp.), among which CLU had the lowest LLOD and LLOQ (0.06 fmol/*μ*L and 0.09 fmol/*μ*L, resp.). By contrast, the LLOD and LLOQ ranges for APLP2, B3GNT1, CD14, and GSN were higher (2.57–5.96 fmol/*μ*L and 7.71–17.88 fmol/*μ*L, resp.), and CD14 had the highest LLOD and LLOQ (5.96 fmol/*μ*L and 17.88 fmol/*μ*L, resp.) (Table 1 and Supplementary Figures  4A and 4B).

**Figure 7:**
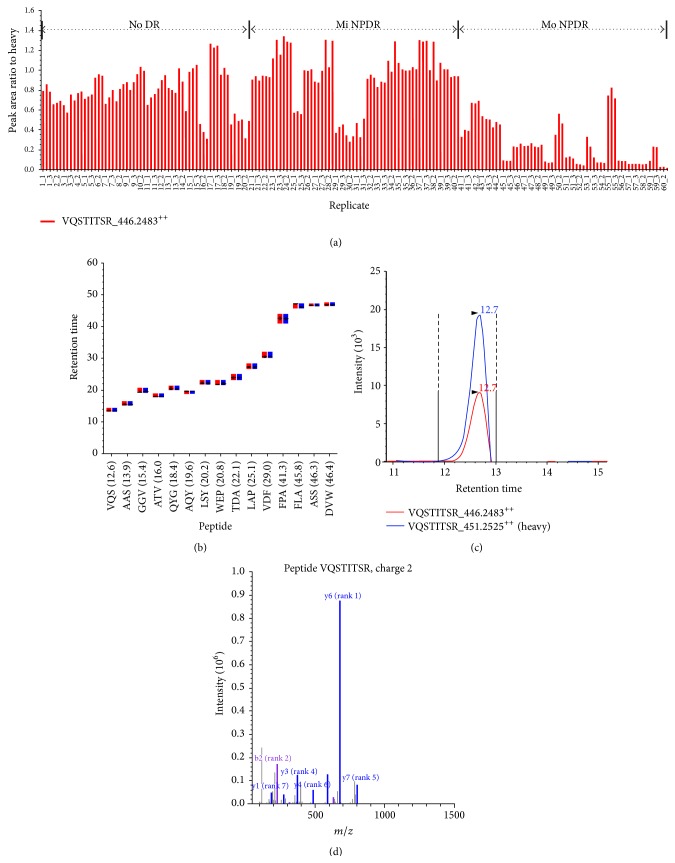
Representative MRM results of the ITIH2 analysis in the No DR, Mi NPDR, and Mo NPDR groups. A representative MRM result for ITIH2-peptide (VQSTITSR_2^++^) is shown. The differential concentration range in 60 individual plasmas (a), elution time points for each peptide (b), XIC for the heavy and light peptides of VQSTITSR_2^++^ (c), and the MS/MS spectrum for fragmented peptides (d) are indicated. In our individual SID-MRM analysis, the CV% for retention time (RT) in the No DR, Mi NPDR, and Mo NPDR groups showed ranges of 0.87–8.35, 0.23–4.81, and 0.11–4.92, respectively. All images were extracted using the Skyline program.

Using the heavy peptide mixture (50 fmol/*μ*L) of the 15 proteins as an internal standard, individual plasma samples were validated using SID-MRM analysis. In this analysis of individual samples, we could confirm a differential concentration of these proteins among the No DR (*n* = 20), Mi NPDR (*n* = 20), and Mo NPDR (*n* = 20) groups.  Figure 7 shows a representative result of the individual SID-MRM analysis for the ITIH2_peptide (VQSTITSR_2^++^), depicting a differential concentration range in 60 individual plasma samples, the average elution time for 15 peptides, an XIC for heavy and light peptides of a peptide (VQSTITSR_2^++^), and a MS/MS spectrum for a fragmented peptide (VQSTITSR_2^++^). In our individual SID-MRM analysis, the CV% for retention time (RT) in the No DR, Mi NPDR, and Mo NPDR groups showed ranges of 0.87–8.35, 0.23–4.81, and 0.11–4.92 min, respectively (Table 1 and Supplementary Figure  5).

**Figure 8:**
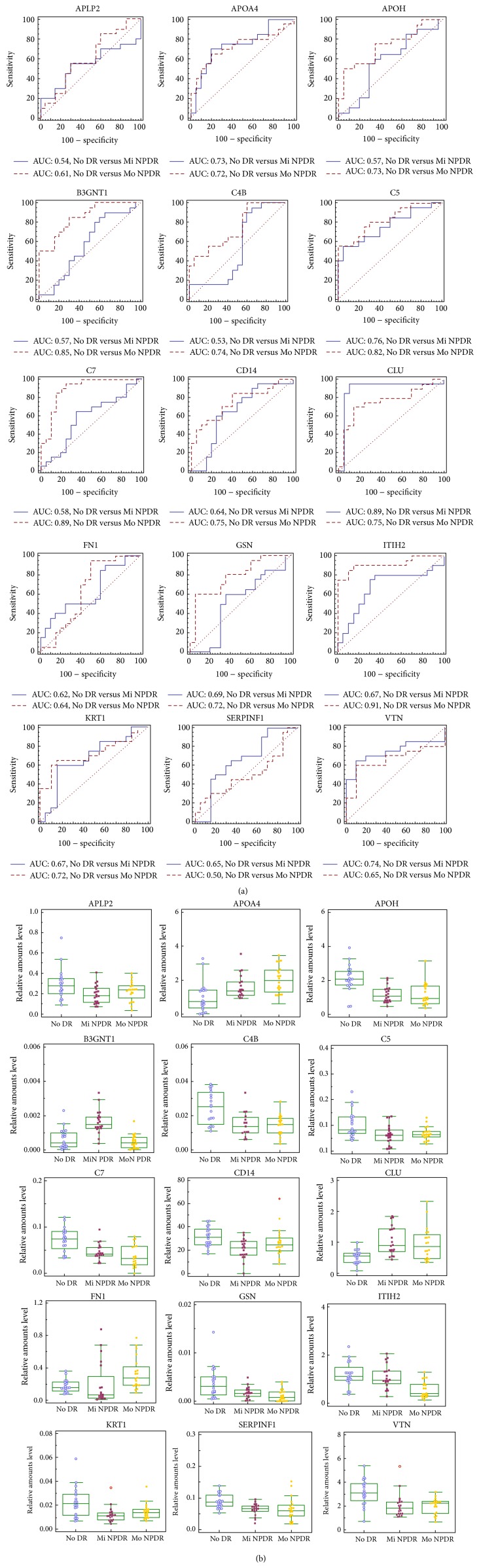
Verification of biomarker candidates using SID-MRM analysis. The 15 selected proteins from our SQ-MRM analysis underwent a second round of verification using SID-MRM analysis in the No DR (*N* = 20), Mi NPDR (*N* = 20), and Mo NPDR (*N* = 20) plasma samples. Box plots (a) and ROC with AUC values (b) were generated based on the SID-MRM analysis.

In this quantitative analysis (Figures 8(a) and 8(b)), the endogenous concentrations of 12 proteins (APOA4, APOH, B3GNT1, C4B, C5, C7, CD14, CLU, GSN, ITIH2, KRT1, and VTN) were significantly different between the No DR and Mi NPDR (AUC value > 0.7) and the No DR and Mo NPDR (AUC value > 0.7) groups. By contrast, no significant difference in the concentrations of 3 proteins (APLP2, FN1, and SERPINF1) was observed between the No DR and NPDR groups.

### 3.7. Construction of a Multimarker Panel Based on the SID-MRM Results

Statistical analyses was carried out using the SID-MRM data for the 15 proteins, which were obtained from patients with No DR (*n* = 20), Mi NPDR (*n* = 20), and Mo NPDR (*n* = 20). First, we chose a multimarker panel that showed the best discriminatory power between the No DR and Mo NPDR groups. We then applied the multimarker panel to evaluate its discriminatory power between the No DR and Mi + Mo NPDR groups. Because Mi NPDR represents an early stage of NPDR, it was not easy to observe differences between the No DR and Mi NPDR groups. To screen NPDR, it may be more reliable to screen Mo NPDR than Mi NPDR. Therefore, to determine the performance of the multimarker panel, we compared the No DR and Mo NPDR samples to measure its classification power.

Student's* t*-test was performed to compare the No DR and Mo NPDR groups, and 11 proteins showed significant differences (APOA4, APOH, B3GNT1, C4B, C5, C7, CD14, CLU, GSN, ITIH2, and KRT1). Before constructing the multimarker panel, we conducted a correlation analysis between all of the markers to test for multicollinearity among the 11 candidates that we verified by SID-MRM analysis using linear regression analysis. Multicollinearity generally indicates that there is a high correlation of at least one independent variable with a combination of the other independent variables. The variation inflation factor (VIF) and tolerance of the 11 candidate ranged from 1.5 to 7.2 and 0.13 to 0.65, respectively (Supplementary Figure  6A). Generally, multicollinearity becomes problematic when the VIF value is greater than 10 and the tolerance is close to 0.

To construct prediction models using the variables that contribute to the discrimination power, we performed stepwise MANOVA to determine whether each candidate should be included in a multimarker panel. The MANOVA test not only could reveal the significant differences between 2 groups but also showed relationships between 2 dependent variables and independent or dependent variables. From the stepwise MANOVA (Supplementary Figure  6B), 4 verified markers (ITIH2, APOA4, C7, and CLU) were selected for inclusion in a multimarker panel.

### 3.8. Evaluation of a 4-Protein Multimarker Panel in the No DR versus Mo NPDR Comparison

**Figure 9:**
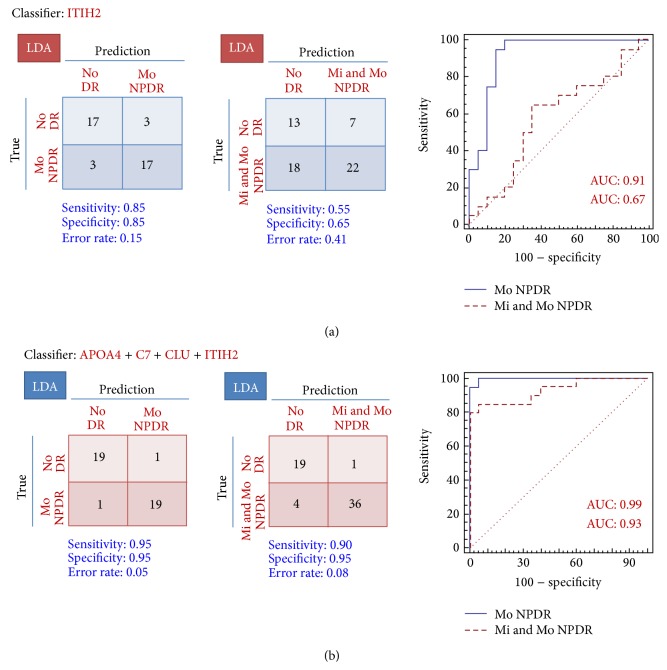
A comparison of the discriminatory power of the 4-marker panel with the best single marker among the No DR, Mo NPDR, and Mi + Mo NPDR groups. We selected 4 proteins from* t*-test and stepwise MANOVA analyses and used them to construct a 4-marker panel. For comprehensive statistical analysis, LDA algorithms were employed where leave-one-out cross validation (LOOCV) was used to evaluate the discriminatory power among the No DR, Mo NPDR, and Mi + Mo NPDR groups. For comparisons between the best single marker and 4-marker panel, results are presented as confusion matrices. Sensitivity, specificity, error rates, and ROC curves are also represented with AUC values.

The 4 verified markers were used to construct a 4-marker panel, which was evaluated for its discriminatory performance. An algorithm of LDA was used to examine the discriminatory power in the No DR versus Mo NPDR comparison; subsequently, LOOCV was used to avoid overfitting. In the LDA model of the 4-marker panel, 19 of 20 patients in both the No DR and the Mo NPDR groups were correctly classified with an error rate of 5% (Figure 9(b)). To compare the discriminatory power of the 4-marker panel with the single best marker (ITIH2), a model coupled with LDA for the single best marker (ITIH2) was also built in which LOOCV was performed in the No DR versus Mo NPDR comparison (Figures 9(a) and 9(b)). As indicated by the SID-MRM results (Figure 8(a)), ITIH2 showed the highest AUC value among the 15 verified candidate proteins and was therefore selected as the best single marker.

In the LDA model, for the parameters of sensitivity (0.95), specificity (0.95), error rate (5%), and AUC value (0.99), the 4-marker panel was superior to the best single marker (ITIH2) (Figure 9(b)). The best single marker model showed a lower sensitivity (0.85), specificity (0.85), AUC value (0.91), and a higher error rate (15%) when compared to the 4-marker panel.

### 3.9. Evaluation of the 4-Marker Panel in the No DR versus Mi + Mo NPDR Comparison

Next, we used the 4-marker panel to evaluate its discriminatory power in the No DR versus Mi + Mo NPDR comparison. In the LDA model, the 4-marker panel showed that 19 of 20 No DR patients and 36 of 40 Mi + Mo NPDR patients were correctly classified, while the best single marker showed that 13 of 20 No DR patients and 22 of 40 Mi + Mo NPDR patients were correctly classified. The 4-marker panel (sensitivity, 0.90; specificity, 0.95; error rate, 8%; and AUC, 0.93) showed better performance than the best single marker (sensitivity, 0.55; specificity, 0.65; error rate, 41%; and AUC, 0.67) (Figures 9(a) and 9(b)).

## 4. Discussion

### 4.1. Selection of DR Biomarker Candidates from the First MRM Analysis

To select biomarker candidates, we performed data-mining on previously published DR-related studies and our experimental data. We included human, mouse, and rat datasets and selected 1202 proteins (data not shown).

However, considering the low number of common proteins between human vitreous fluid and the retina of animal models, along with the use of human plasma in the biomarker validation step, we decided to only use the human data-mining data sets for the selection of biomarker candidates. From the human data-mining analysis, a total of 490 proteins were identified. To confirm the MRM transition, 96 proteins (387 peptides and 1870 transitions) were ultimately selected.

Based on a MASCOT search to confirm each transition in pooled plasma, 39 proteins were selected, including 22 proteins that had 2 peptides and 17 proteins that had 1 peptide (Supplementary Table  6). Briefly, 1870 transitions (387 peptides) for 96 proteins that were found in pooled plasma were selected. Based on the MASCOT search results, 296 transitions (61 peptides) of 1870 transitions (387 peptides) were finally identified as peptides derived from the original protein. The data indicated that 319 (82.4%) of 387 peptides were coeluted with other peptides, while 61 (15.8%) of 387 peptides were true peptides derived from the original proteins, which is a very low recovery efficiency. According to Addona et al. [34], the most common sample-related source of quantification errors in MRM is ion interference from other coeluting peptides.

The 39 candidates were then examined in the SQ-MRM quantification analysis, and we found 15 proteins that were significantly differentially expressed between the No DR, Mi NPDR, and Mo NPDR groups.

### 4.2. A Comparison of the Mean Concentrations in the No DR, Mi NPDR, and Mo NPDR Groups

To measure the concentration of the 15 proteins in the No DR (*n* = 20), Mi NPDR (*n* = 20), and Mo NPDR (*n* = 20) groups, we first determined the LLOQ range using the pooled plasma samples. For the 4 proteins (APOA4, CLU, ITIH2, and VTN) that had a very low LLOQ range (0.19–0.96 fmol/*μ*L), all of the measured average concentrations had a higher LLOQ range in the No DR, Mi NPDR, and Mo NPDR groups (Table 2 and Supplementary Figures  4B and  7). To compare the concentration ranges between the normal and NPDR groups, we identified the normal plasma concentrations reported in the literature [9, 35–38]. The previously reported concentrations of APOA4 [35] and ITIH2 [36] were higher or similar when compared to the No DR, Mi NPDR, and Mo NPDR groups, while the reported concentrations of CLU [35, 36] and VTN [35, 36] were lower than in the DR groups reported here (Table 2 and Supplementary Figure  7).

By contrast, APLP2, B3GNT1, CD14, and GSN had a higher LLOQ range (7.71–17.88 fmol/*μ*L). The average concentrations of CD14 and APLP2 in the No DR, Mi NPDR, and Mo NPDR groups were higher than the LLOQ concentration range, whereas the average concentrations of B3GNT1 and GSN were lower than the LLOQ concentration range. For APLP2 and B3GNT1, we assumed that the high LLOD levels resulted from low endogenous light peptide concentrations and high levels of noise signals. The concentrations of CD14 [38] and GSN [36] in normal plasma were higher than the average concentrations in the No DR, Mi NPDR, and Mo NPDR groups (Table 2 and Supplementary Figure  7). However, there were no values available in the literature for the normal plasma concentration ranges of APLP2 and B3GNT1, so we could not make a comparison between the reported and measured concentrations.

Among the 15 selected proteins, the other 7 (APOH, C4B, C5, C7, FN1, KRT1, and SERPINF1) had a mid-LLOQ range (1.01–3.93 fmol/*μ*L) in this study. The average measured concentrations for all 7 proteins in the No DR, Mi NPDR, and Mo NPDR groups were higher than the LLOQ range (Table 2 and Supplementary Figure  7). The previously reported concentration ranges of C7 [9, 36] and FN1 [9, 35–37] were higher in normal plasma, whereas those of C4B [36] and SERPINF1 [36] were lower than the average concentration in the No DR, Mi NPDR, and Mo NPDR groups (Table 2 and Supplementary Figure  7). The previously published concentration of APOH [36] in normal plasma was similar to the measured average concentrations in the No DR, Mi NPDR, and Mo NPDR groups (Table 2 and Supplementary Figure  7). For C5 [36], its concentration in normal plasma was lower in the No DR and Mi NPDR groups, while its level was higher in the Mo NPDR group (Table 2 and Supplementary Figure  7). The normal plasma concentration range of KRT1 has not been previously reported.

In the quantitative analysis using 60 individual samples (Figures 8(a) and 8(b)), the concentrations of 12 proteins (APOA4, APOH, B3GNT1, C4B, C5, C7, CD14, CLU, GSN, ITIH2, KRT1, and VTN) were significantly different in the No DR versus Mi NPDR (AUC value > 0.7) and No DR versus Mo NPDR (AUC value > 0.7) comparisons. By contrast, no significant difference in the concentration of 3 proteins (APLP2, FN1, and SERPINF1) was detected among the No DR, Mi NPDR, and Mo NPDR groups.

### 4.3. Evaluation of the 4-Marker Panel among the No DR, Mi NPDR, and Mo NPDR Groups

To improve the classification discriminating power between the No DR and NPDR groups, we constructed a multimarker panel and subjected it to statistical evaluation. A similar approach has been carried out to identify a novel biomarker that can distinguish disease status between affected and healthy groups; a multimarker panel that included more than 1 protein showed better performance than a single marker [33, 39–42].

Before we selected variables for the multimarker panel, we first considered which combination of sample groups (No DR, Mi NPDR, and Mo NPDR) would show the best discriminating power. Mi NPDR is a very early stage of NPDR and it was not easy to observe differences in the No DR group versus the Mi NPDR group. However, Mo NPDR is a more advanced stage of NPDR and may be more representative of a NPDR diagnosis than Mi NPDR. Thus, the detection of Mo NPDR might be more suitable for NPDR screening. Therefore, we first selected a multimarker panel that showed the best discriminatory power between the No DR and Mo NPDR groups. We then applied this multimarker panel to evaluate its discriminatory power in No DR versus the Mi + Mo NPDR.

We first determined the correlation and multicollinearity among the 15 potential markers as variables to evaluate performance. Thereafter, we selected 4 potential markers that showed significantly different patterns in the No DR and Mo NPDR groups using Student's* t*-test and stepwise MANOVA. Using the 4 significantly different marker proteins (ITIH2, APOA4, C7, and CLU), the multimarker panel was constructed and statistical validation was performed using LOOCV. The LDA method was employed for statistical analysis of the model.

In a comparison of the No DR group with the Mo NPDR group, the 4-marker panel showed better sensitivity (0.95), specificity (0.95), error rate (5%), and AUC value (0.99) than the best single marker (ITIH2). Indeed, the single best candidate model showed lower sensitivity (0.85), specificity (0.85), AUC value (0.91), and a higher error rate (15%). Moreover, for the No DR versus Mi + Mo NPDR comparison, the 4-marker panel (sensitivity, 0.90; specificity, 0.95; error rate, 8%; and AUC, 0.93) also showed better performance than the best single marker (sensitivity, 0.55; specificity, 0.65; error rate, 41%; and AUC, 0.67). These data demonstrate that the discriminatory power of the 4-marker marker panel was higher than the best single marker model.

Finally, we suggest that the multimarker panel (ITIH2, APOA4, C7, and CLU) is able to distinguish DR status between the patient and normal groups. However, more precise validation with a larger sample size is needed. Further validation in a larger sample size might yield a better understanding of statistical analysis and correlated variables, facilitating the development of better biomarkers for DR.

## 5. Conclusion

We have performed a comprehensive proteomics study to identify biomarkers for DR. We first performed data-mining on the previously published DR-related studies and our experimental data; 96 proteins were then selected. To verify the selected biomarker candidates in plasma, candidates were selected, confirmed, and validated in the plasma of patients in the No DR, Mi NPDR, and Mo NPDR groups using SQ-MRM and SID-MRM analyses. In the final stage, we constructed a model for a multimarker panel using the 11 verified markers derived from our SID-MRM analysis. Our multimarker panel showed merged AUC values of 0.99 (No DR versus Mo NPDR) and 0.93 (No DR versus Mi + Mo NPDR). The 4-protein marker panel (APO4, C7, CLU, and ITIH2) can be used as baseline data for the discovery of novel biomarkers for detecting early stage DR and for further validation using a larger panel of proteins.

## Supplementary Material

For identification of protein, an enhanced mass scan (EMS) ranging from 400 to 1200 m/z (scan speed, 4000 Da/s) was performed as an first MRM step, and an enhanced product ion (EPI) scan was then carried out to obtain MS/MS spectra. A MASCOT search was performed using the MS/MS spectra to identify the protein.
Mascot.dll 1.6b23 and ABSciex.DataAccess.Wiff File DataReader.dll were used for importing data to Mascot and SwissProt 57.12 was used for database (513877 entries). The other database search parameters used for searching were the following: enzyme trypsin, 1 missed cleavage, variable carbamidomethylation modifications (C), oxidation (M), peptide tolerance 0.15 Da, MS/MS tolerance 0.1 Da, peptide charge 1+, and monoisotopic. Only significant hits at a *p*<0.05, as defined by MASCOT probability analysis, were accepted.

## Figures and Tables

**Table 1:** Experimental MRM conditions for 15 selected proteins.

*N*	Protein	Accession number	Gene	Peptide sequence		Peptide		Charge stage		Ions^c^	DP^d^	CE^e^ (V)	RT^f^ (min)
				(light^a^ and heavy^b^)		Q1	Q3	Q1	Q3				
1	Amyloid-like protein 2	Q06481	APLP2	Light	WEPDPTGTK	515.74	503.28	2	1	**y5**, y6, y8	123	28	20.8
				Heavy	WEPDPTGT**[13C-15N-K]**	519.75	511.29	2	1				

2	Apolipoprotein A-IV	P06727	APOA4	Light	LAPLAEDVR	492.28	589.29	2	1	**y5**, y6, y7	123	27	25.1
				Heavy	LAPLAEDV**[13C-15N-R]**	497.28	599.30	2	1				

3	Beta-2-glycoprotein 1	P02749	APOH	Light	ATVVYQGER	511.80	652.30	2	1	**y5**, y6, y7	123	30.52	16
				Heavy	ATVVYQGE**[13C-15N-R]**	516.77	662.31	2	1				

4	N-Acetyllactosaminide beta-1,3-N-Acetylglucosaminyltransferase	O43505	B3GNT1	Light	QYGFNR	392.69	656.31	2	1	y3, y4, **y5**	123	22	18.4
				Heavy	QYGFN**[13C-15N-R]**	397.69	666.32	2	1				

5	Complement C4-B	P0C0l5	C4B	Light	VDFTLSSER	527.26	839.43	2	1	y5, y6,** y7**	69.6	25.8	29
				Heavy	VDFTLSSE**[13C-15N-R]**	532.27	849.43	2	1				

6	Complement C5	P01031	C5	Light	TDAPDLPEENQAR	728.34	843.39	2	1	**y7**, y9, y10	84.2	37.3	22.1
				Heavy	TDAPDLPEENQA**[13C-15N-R]**	733.34	853.40	2	1				

7	Complement component C7	P10643	C7	Light	AASGTQNNVLR	565.79	743.41	2	1	y5, **y6**, y9	72.4	28	13.9
				Heavy	AASGTQNNVL**[13C-15N-R]**	570.80	753.42	2	1				

8	Monocyte differentiation antigen CD14	P08571	CD14	Light	FPAIQNLALR	571.83	714.42	2	1	**y6**, y7, y8	72.8	28.3	41.3
				Heavy	FPAIQNLAL**[13C-15N-R]**	576.84	724.43	2	1				

9	Clusterin	P10909	CLU	Light	ASSIIDELFQDR	697.35	1035.51	2	1	y6, y7, **y8**	123	38.69	46.3
				Heavy	ASSIIDELFQD**[13C-15N-R]**	702.35	1045.52	2	1				

10	Fibronectin	P02751	FN1	Light	FLATTPNSLLVSWQPPR	964.02	1393.75	2	1	y3, y8,** y12**	101.4	50.7	45.8
				Heavy	FLATTPNSLLVSWQPP**[13C-15N-R]**	969.02	1403.77	2	1				

11	Gelsolin	P06396	GSN	Light	GGVASGFK	361.70	509.27	2	1	y4,** y5**, y6	123	21	15.4
				Heavy	GGVASGF**[13C-15N-K]**	365.70	517.29	2	1				

12	Inter-alpha-trypsin inhibitor heavy chain H2	P19823	ITIH2	Light	VQSTITSR	446.24	664.36	2	1	y4, y5, **y6**	63.6	21.2	9.41
				Heavy	VQSTITS**[13C-15N-R]**	451.25	674.37	2	1				

13	Keratin, type II cytoskeletal 1	P04264	KRT1	Light	AQYEDIAQK	533.26	866.42	2	1	y5, y6, **y7**	123	28	19.6
				Heavy	AQYEDIAQ**[13C-15N-K]**	537.27	874.44	2	1				

14	Pigment epithelium-derived factor	P36955	SERPINF1	Light	LSYEGEVTK	513.26	662.35	2	1	y5, **y6**, y7	123	28	20.2
				Heavy	LSYEGEVT**[13C-15N-K]**	517.27	670.35	2	1				

15	Vitronectin	P04004	VTN	Light	DVWGIEGPIDAAFTR	823.91	947.49	2	1	y8, **y9**, y13	123	41	46.4
				Heavy	DVWGIEGPIDAAFT**[13C-15N-R]**	828.91	957.50	2	1				

^a^Light peptides represent endogenous peptides in plasma. ^b^heavy peptides represent SIS peptides (>95% purity). ^c^Ions represent the top 3 ions, where bold type indicates the ions used in quantitation. ^d^DP, ^e^CE, and ^f^RT represent declustering potential, collisional energy, and average retention time, respectively.

**Table 2:** The linearity, sensitivity, and analytical reproducibility of the MRM assay.

*N*	Name	Peptide sequence		Average relative response (L/H)^a^	Correlation coefficient (*R* ^2^)^b^	Dynamic range	RT (min)^c^	RT CV%^d^			LLOD (fmol/*µ*L)^e^	LLOQ (fmol/*µ*L)^f^	Normal con. (ng/mL)^g^	Average con. ng/*µ*L			Conc. reference
		(heavy and light)						No DR	Mi NPDR	Mo NPDR				No DR	Mi NPDR	Mo NPDR	
1	APLP2	Light	WEPDPTGTK	0.01	0.967	1,000	20.8	7	3.84	3.8	5.88	17.66	·	0.61	0.73	0.45	
		Heavy	WEPDPTGT**[13C-15N-K]**					7.28	3.8	2.44							

2	APOA4	Light	LAPLAEDVR	2.99	0.995	2,000	25.1	5.3	3.82	2.47	0.13	0.41	160,000	125.28	149.92	166.04	[29]
		Heavy	LAPLAEDV**[13C-15N-R]**					5.38	3.87	2.46							

3	APOH	Light	ATVVYQGER	5.39	0.999	2,000	16	7.58	4.39	4.3	1.1	3.57	258,000	254.86	269.92	190.82	[30]
		Heavy	ATVVYQGE**[13C-15N-R]**					7.53	4.47	4.21							

4	B3GNT1	Light	QYGFNR	0.95	0.941	1,000	18.4	6.77	4.14	3.72	2.57	7.71	·	51.98	47.74	34.24	
		Heavy	QYGFN**[13C-15N-R]**					6.72	4.03	3.78							

5	C4B	Light	VDFTLSSER	4.78	0.999	2,000	29	4.86	2.99	1.73	0.32	1.01	95,000	266.04	239.1	193.78	[30]
		Heavy	VDFTLSSE**[13C-15N-R]**					4.85	2.91	1.7							

6	C5	Light	TDAPDLPEENQAR	2.49	0.998	2,000	22.1	7.03	3.62	1.71	0.38	1.14	69,000	189.66	124.84	15.74	[30]
		Heavy	TDAPDLPEENQA**[13C-15N-R]**					7.04	3.56	1.77							

7	C7	Light	AASGTQNNVLR	0.44	0.989	2,000	13.9	8.24	4.8	4.92	0.52	1.57	60,000	24.27	22.49	13.76	[9, 30]
		Heavy	AASGTQNNVL**[13C-15N-R]**					8.25	4.81	4.84							

8	CD14	Light	FPAIQNLALR	0.085	0.993	1,000	41.3	2.81	1.72	1.27	5.96	17.88	5,000	4.26	4.28	3.03	[32]
		Heavy	FPAIQNLAL**[13C-15N-R]**					2.91	1.72	1.26							

9	CLU	Light	ASSIIDELFQDR	3.3	0.998	1,000	46.3	0.94	0.23	0.11	0.06	0.19	100,000	115.98	165.18	143.37	[30, 33]
		Heavy	ASSIIDELFQD**[13C-15N-R]**					0.95	0.26	0.12							

10	FN1	Light	FLATTPNSLLVSWQPPR	0.3	0.994	2,000	45.8	0.95	0.25	0.13	1.31	3.93	275,000	19.75	15.16	22.93	[9, 29–31]
		Heavy	FLATTPNSLLVSWQPP**[13C-15N-R]**					0.94	0.28	0.12							

11	GSN	Light	GGVASGFK	0.59	0.998	2,000	15.4	7.78	4.43	4.46	4.15	12.46	240,000	29.64	29.64	19.87	[30]
		Heavy	GGVASGF**[13C-15N-K]**					7.94	4.34	4.55							

12	ITIH2	Light	VQSTITSR	0.9	0.996	2,000	9.41	7.2	3.07	3.91	0.19	0.59	140,000	39.32	45.44	17.41	[30]
		Heavy	VQSTITS**[13C-15N-R]**					7.25	3.09	3.96							

13	KRT1	Light	AQYEDIAQK	0.26	0.987	2,000	19.6	8.19	4.14	3.37	1.28	3.85	·	11.56	13.24	8.04	
		Heavy	AQYEDIAQ**[13C-15N-K]**					8.35	4.35	3.35							

14	SERPINF1	Light	LSYEGEVTK	0.87	0.996	2,000	20.2	6.88	3.79	2.44	0.8	2.41	4,600	47.11	43.8	41.28	[30]
		Heavy	LSYEGEVT**[13C-15N-K]**					6.95	4.52	3.47							

15	VTN	Light	DVWGIEGPIDAAFTR	5.67	0.987	1,000	46.4	0.87	0.23	0.17	0.32	0.96	100,000	223.49	283.84	257.51	[30]
		Heavy	DVWGIEGPIDAAFT**[13C-15N-R]**					0.89	0.82	0.58							

^a^Average relative response (L.H) represents the peak area ratio of light to heavy signal. ^b^The correlation coefficient for each protein (*R* ^2^) represents the linearity of the MRM analysis. ^c^Retention time (RT) represents the average retention time in 60 individual samples. ^d^RT CV% represents the retention time variation in the No DR, Mi NPDR, and Mo NPDR groups. ^e^Lowest limit of detection (LLOD) is calculated based on the variance of the blank sample and the standard deviation (S.D.) of the sample with the lowest “spiked” in concentration. ^f^The lowest limit of quantitation (LLOQ) was calculated as follows: LLOQ = LLOD × 3. ^g^Normal protein concentrations in plasma were obtained by literature searches as described in the Results section.
