# Supplementary material for: Development of Diagnostic Biomarkers for Detecting Diabetic Retinopathy at Early Stages Using Quantitative Proteomics
Source: J Diabetes Res. 2015 Nov 9;2016:6571976. doi: 10.1155/2016/6571976 (PMC4657408; doi:10.1155/2016/6571976)
Supplement: Supplementary file 1 — For identification of protein, an enhanced mass scan (EMS) ranging from 400 to 1200 m/z (scan speed, 4000 Da/s) was performed as an first MRM step, and an enhanced product ion (EPI) scan was then carried out to obtain MS/MS spectra. A MASCOT search was performed using the MS/MS spectra to identify the protein. Mascot.dll 1.6b23 and ABSciex.DataAccess.Wiff File DataReader.dll were used for importing data to Mascot and SwissProt 57.12 was used for database (513877 entries). The other database search parameters used for searching were the following: enzyme trypsin, 1 missed cleavage, variable carbamidomethylation modifications (C), oxidation (M), peptide tolerance 0.15 Da, MS/MS tolerance 0.1 Da, peptide charge 1+, and monoisotopic. Only significant hits at a p<0.05, as defined by MASCOT probability analysis, were accepted. [file 6571976.f1.zip › Supplementary Figures.pdf]

Supplementary Figures

Supplementary Fig. 1. Functional analysis of 15 verified proteins from preliminary MRM analysis

The 15 verified proteins from the preliminary MRM analysis were classified under the ʺbiological processʺ and ʺmolecular functionʺ categories using the PANTHER classification program (A). The largest components in ʺbiological processʺ were ʺimmunity and defenseʺ (33%) and ʺmolecular functionʺ (20%), respectively. To investigate the signaling pathway related to the 15 verified proteins, we conducted signaling pathway analysis. Consequently, 2 signaling pathways (actin cytoskeleton and complement cascade pathway) were selected from the KEGG database (B and C).

Supplementary Fig. 2. Correlation of peak area values between two peptides per protein

The peak area values between two peptides per protein were compared in SQ-MRM experiments. The peak similarities of two peptides were analyzed by Pearsonʹs correlation analysis. The patterns of peak intensity were highly correlated between two peptides in each protein showing the median value of 0.927.

Supplementary Fig. 3. Confirmation of synthesized heavy peptide using the MILDI-TOF

To verify the 15 selected candidates, we first synthesized SIS peptides (>95% purity) for each protein and verified the purity by MALDI TOF analysis. All the SIS heavy peptides had the same theoretical and experimental molecular weight values.

Supplementary Fig. 4. Generation of calibration curve for 15 proteins

To generate a calibration curve for the 15 proteins, heavy peptides were serially diluted (12 concentration points: 0. 1–200 fmol) with the light peptide as an internal standard (digested pooled plasma protein: 1 μg) added into each serially diluted samples. Each experiment was repeated in triplicate to generate the calibration curve. The area ratio of heavy peptide (12 concentration points: 0.1–200 fmol) to light peptide (internal standard: plasma l μg) was determined from the extracted ion chromatogram (XIC) of each selected transition.

Supplementary Fig. 5. Evaluation of retention time stability in No DR, Mi NPDR, and Mo NPDR groups

The CV% of retention time (RT) for 15 peptides are observed in NO DR (N: 20), Mi NPDR (N: 20), and Mo NPDR groups (N: 20). The CV% for RT in three groups is shown in a range of 0.87-8.35, 0.23-4.81, and 0.11-4.92 min, respectively. The averaged RT for 15 peptides (D) represent in a range of 12.6-46.4 min. The peptide of ITIH2_VQSTITSR_y6++ is a first eluted peptide in 15 peptides (12.6 min), while the peptide of APLP2_WEPDPTGTK_y7++ is a last eluted peptide (46.4 min). All images were extracted by Skyline program.

Supplementary Fig. 6. Selection of multi-marker panel by statistical analysis

Multicolinearity among the 11 candidates was investigated using a linear regression analysis (A). To build multi-marker panel, stepwise MANOVA was employed to determine the suitability of candidates for inclusion in a multi-marker panel (B). From the stepwise MANOVA, 4 verified markers (ITIH2, APOA4, C7, and CLU) were selected and used to construct a multi-marker panel. Statistical validation was then performed using LOOCV.

Supplementary Fig. 7. Comparison of mean concentration in No DR, Mi NPDR, and Mo NPDR groups versus normal plasma concentration

To assess the measured concentration from SID-MRM analysis in No DR (n=20), Mi NPDR (n=20), and Mo NPDR (n=20) groups, the each mean concentration level was compared to normal plasma concentration reported in the literatures. 15 proteins were ordered from highest-to-lowest abundance with the LLOQ of each corresponding assay.

Supplementary Table Legends

Supplementary Table 1. Characteristics of clinical plasma samples in the No DR, Mi NPDR, and Mo NPDR groups

Supplementary Table 2. Statistics for clinical plasma sample in the No DR, Mi NPDR, and Mo NPDR groups

Supplementary Table 3. Identified human vitreous proteome in MH, NPDR, and PDR groups

Supplementary Table 4. Differentially expressed proteins in human vitreous

Supplementary Table 5. Data-mining for MRM target selection in human vitreous

Supplementary Table 6. Selection of reliable transitions for MRM analysis

Supplementary Fig. 1

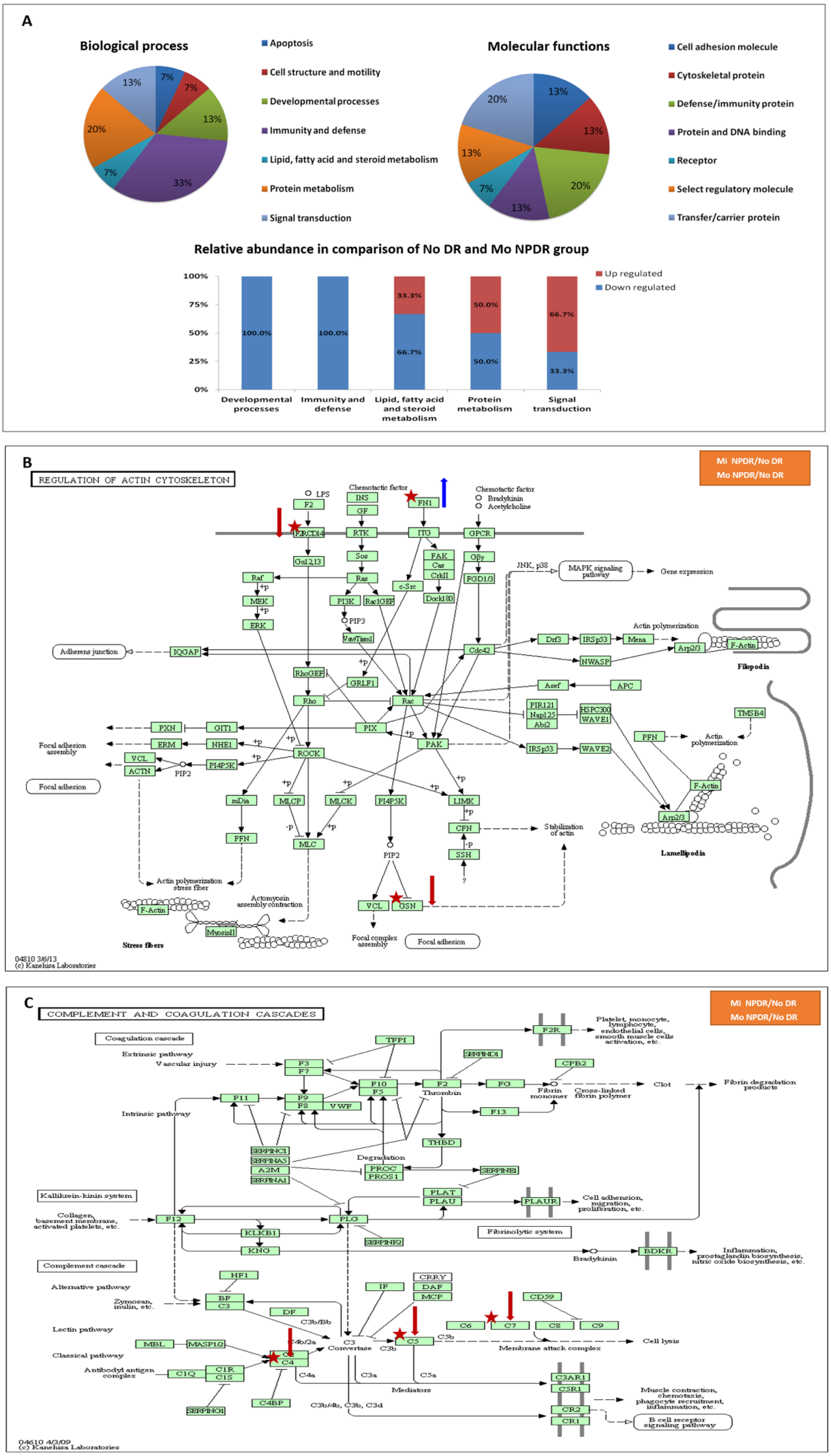
A
Biological process
Apoptosis
Cell structure and motility
Developmental processes
Immunity and defense
Lipid, fatty acid and steroid metabolism
Protein metabolism
Signal transduction
7%
7%
13%
33%
7%
20%
13%
Molecular functions
Cell adhesion molecule
Cytoskeletal protein
Defense/immunity protein
Protein and DNA binding
Receptor
Select regulatory molecule
Transfer/carrier protein
13%
13%
20%
13%
7%
13%
20%
Relative abundance in comparison of No DR and Mo NPDR group
Up regulated
Down regulated
100%
75%
50%
25%
0%
100.0%
100.0%
33.3%
66.7%
50.0%
50.0%
66.7%
33.3%
Developmental processes
Immunity and defense
Lipid, fatty acid and steroid metabolism
Protein metabolism
Signal transduction
B
REGULATION OF ACTIN CYTOSKELETON
Mi NPDR/No DR
Mo NPDR/No DR
Chemotactic factor
Bradykinin
Acetylcholine
LPS
F2
INS
GF
FN1
F2RCD14
RTK
ITG
GPCR
MAPK signaling pathway
Gene expression
Adherens junction
IQGAP
Focal adhesion assembly
Focal adhesion
Actin polymerization
Filopodia
Lamellipodia
Stress fibers
Actin polymerization stress fiber
Actomyosin assembly contraction
Stabilization of actin
Focal complex assembly
VCL
GSN
04810 3/6/13
(c) Kanehisa Laboratories
C
COMPLEMENT AND COAGULATION CASCADES
Mi NPDR/No DR
Mo NPDR/No DR
Coagulation cascade
Extrinsic pathway
Vascular injury
Intrinsic pathway
Kallikrein-kinin system
Fibrinolytic system
Complement cascade
Alternative pathway
Lectin pathway
Classical pathway
Membrane attack complex
Cell lysis
Clot
Fibrin degradation products
Mediators
B cell receptor signaling pathway
C4
C5
C7
04610 4/3/09
(c) Kanehisa Laboratories

Supplementary Fig. 2

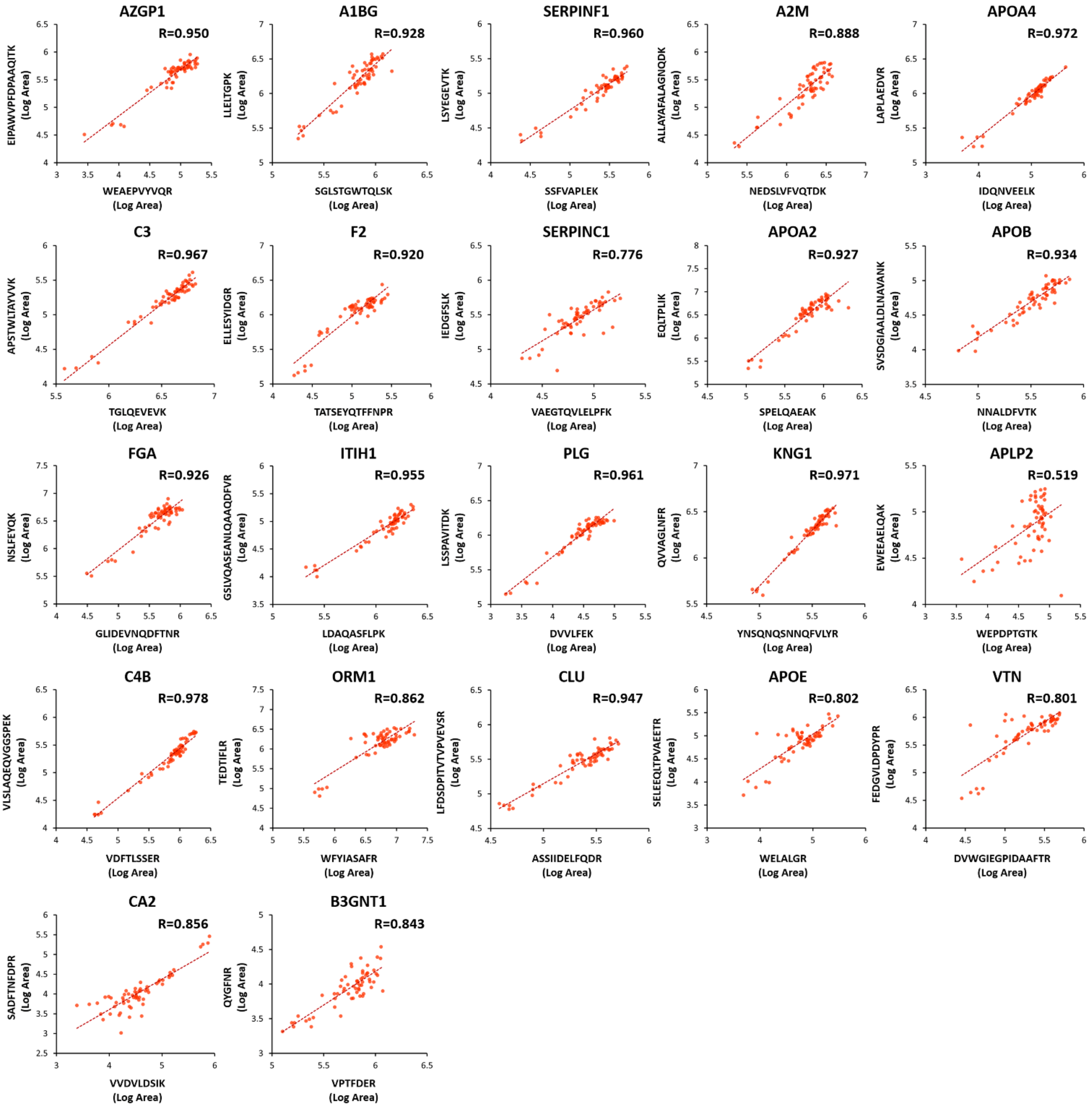

Supplementary Fig. 3

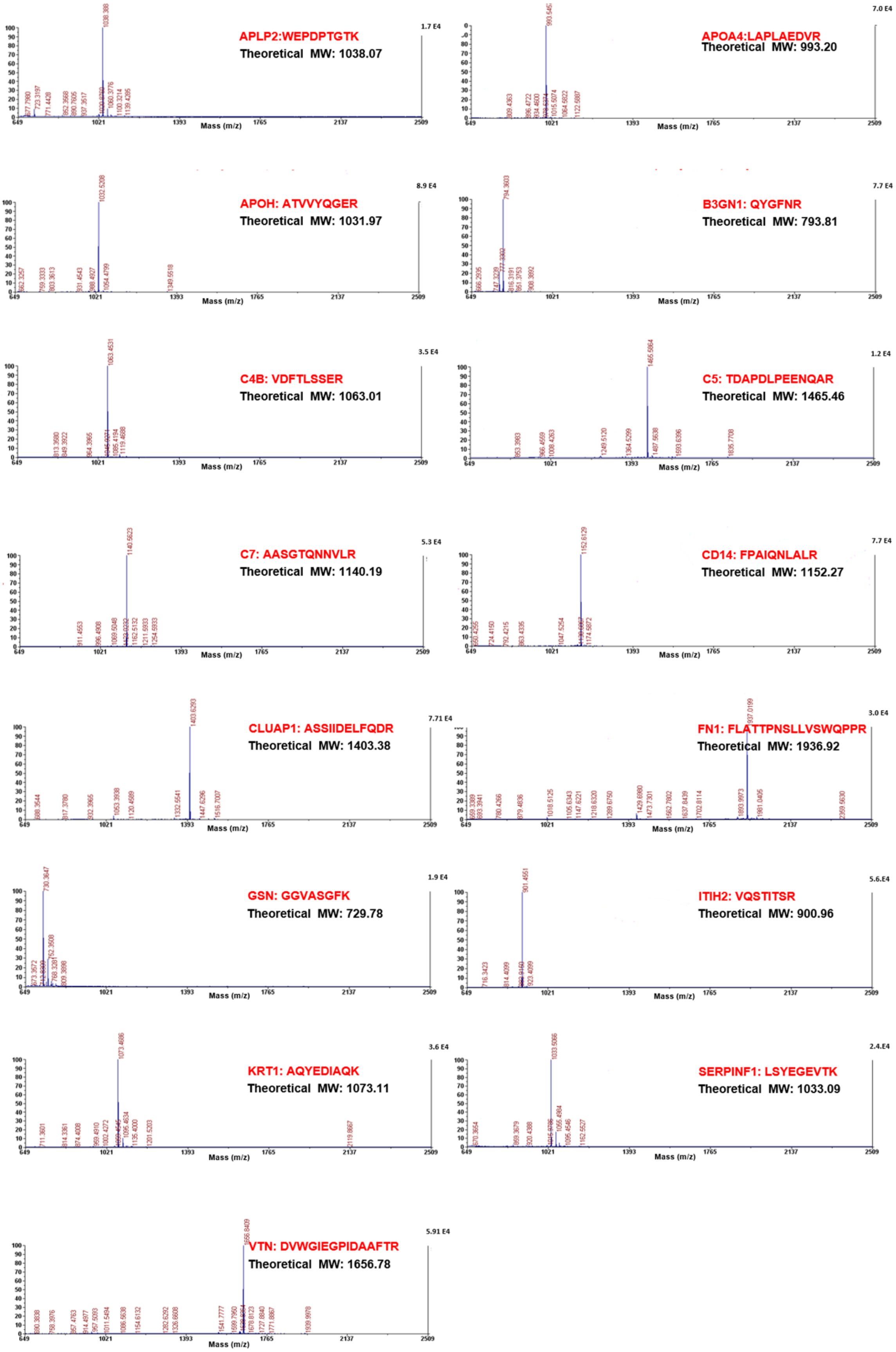
APLP2:WEPDPTGTK
Theoretical MW: 1038.07
APOA4:LAPLAEDVR
Theoretical MW: 993.20
APOH: ATVVYQGER
Theoretical MW: 1031.97
B3GN1: QYGFNR
Theoretical MW: 793.81
C4B: VDFTLSSER
Theoretical MW: 1063.01
C5: TDAPDLPEENQAR
Theoretical MW: 1465.46
C7: AASGTQNNVLR
Theoretical MW: 1140.19
CD14: FPAIQNLALR
Theoretical MW: 1152.27
CLUAP1: ASSIIDELFQDR
Theoretical MW: 1403.38
FN1: FLATTPNSLLVSWQPPR
Theoretical MW: 1936.92
GSN: GGVASGFK
Theoretical MW: 729.78
ITIH2: VQSTITSR
Theoretical MW: 900.96
KRT1: AQYEDIAQK
Theoretical MW: 1073.11
SERPINF1: LSYEGEVTK
Theoretical MW: 1033.09
VTN: DVWGIEGPIDAAFTR
Theoretical MW: 1656.78
Mass (m/z)

Supplementary Fig. 4

A

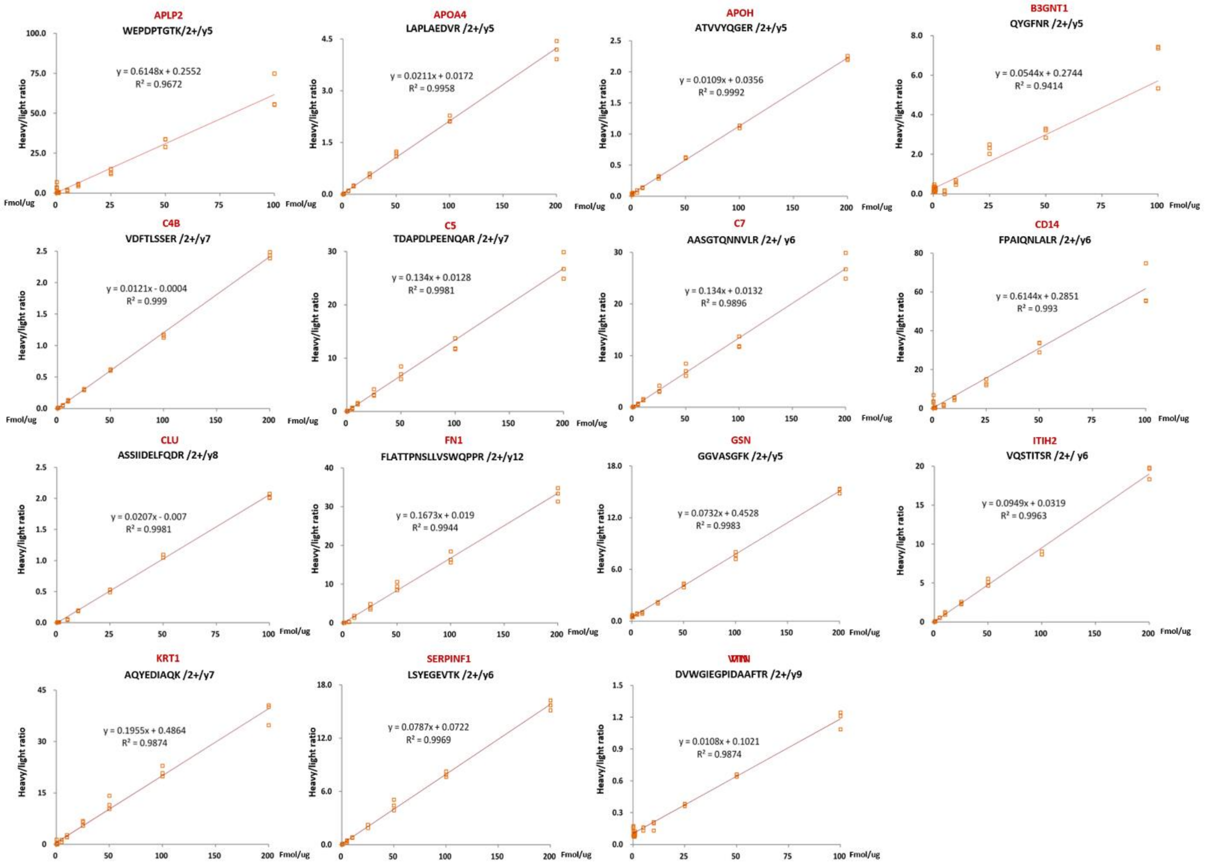

APLP2
WEPDPTGTK/2+/y5
y = 0.6148x + 0.2552
R² = 0.9672
APOA4
LAPLAEDVR /2+/y5
y = 0.0211x + 0.0172
R² = 0.9958
APOH
ATVVYQGER /2+/y5
y = 0.0109x + 0.0356
R² = 0.9992
B3GNT1
QYGFNR /2+/y5
y = 0.0544x + 0.2744
R² = 0.9414
C4B
VDFTLSSER /2+/y7
y = 0.0121x - 0.0004
R² = 0.999
C5
TDAPDLPEENQAR /2+/y7
y = 0.134x + 0.0128
R² = 0.9981
C7
AASGTQNNVLR /2+/ y6
y = 0.134x + 0.0132
R² = 0.9896
CD14
FPAIQNLALR /2+/y6
y = 0.6144x + 0.2851
R² = 0.993
CLU
ASSIIDELFQDR /2+/y8
y = 0.0207x - 0.007
R² = 0.9981
FN1
FLATTPNSLLVSWQPPR /2+/y12
y = 0.1673x + 0.019
R² = 0.9944
GSN
GGVASGFK /2+/y5
y = 0.0732x + 0.4528
R² = 0.9983
ITIH2
VQSTITSR /2+/ y6
y = 0.0949x + 0.0319
R² = 0.9963
KRT1
AQYEDIAQK /2+/y7
y = 0.1955x + 0.4864
R² = 0.9874
SERPINF1
LSYEGEVTK /2+/y6
y = 0.0787x + 0.0722
R² = 0.9969
VTN
DVWGIEGPIDAAFTR /2+/y9
y = 0.0108x + 0.1021
R² = 0.9874
Heavy/light ratio
Fmol/ug

B

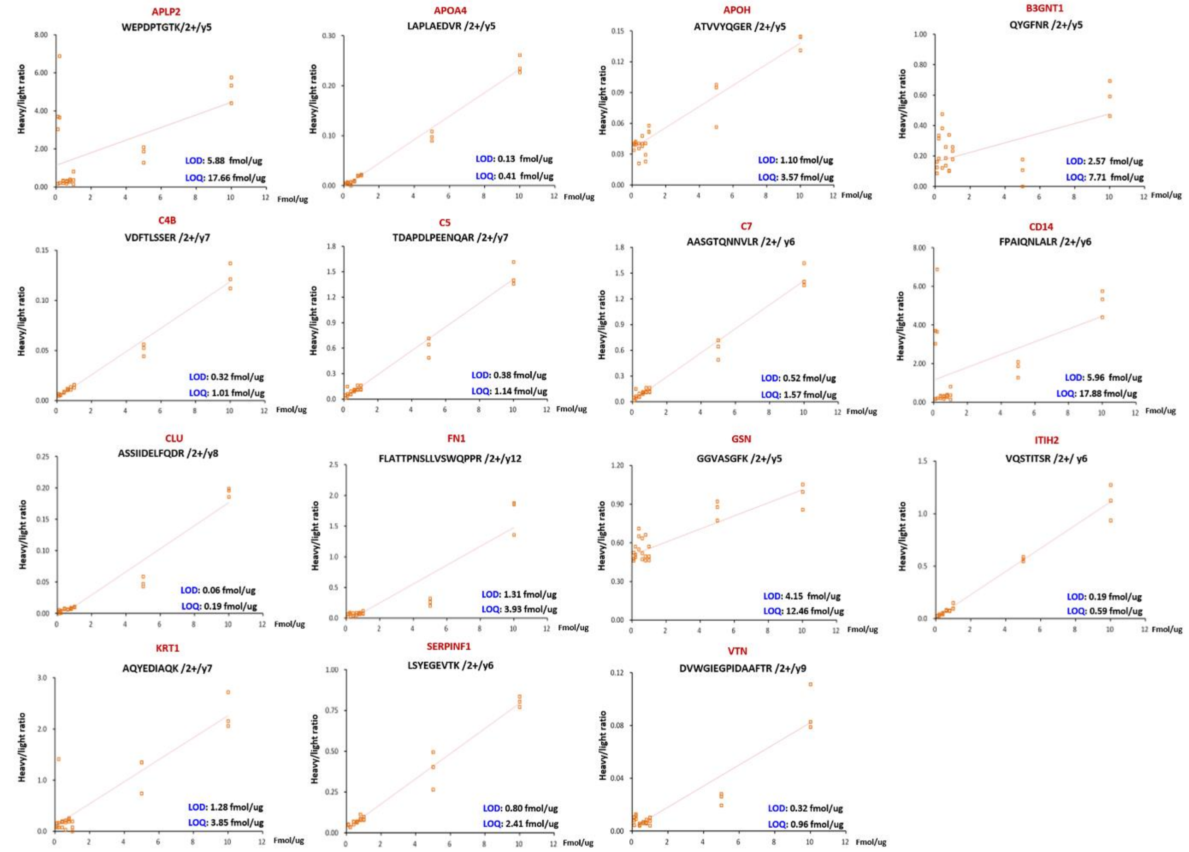

APLP2
WEPDPTGTK/2+/y5
LOD: 5.88 fmol/ug
LOQ: 17.66 fmol/ug
APOA4
LAPLAEDVR /2+/y5
LOD: 0.13 fmol/ug
LOQ: 0.41 fmol/ug
APOH
ATVVYQGER /2+/y5
LOD: 1.10 fmol/ug
LOQ: 3.57 fmol/ug
B3GNT1
QYGFNR /2+/y5
LOD: 2.57 fmol/ug
LOQ: 7.71 fmol/ug
C4B
VDFTLSSER /2+/y7
LOD: 0.32 fmol/ug
LOQ: 1.01 fmol/ug
C5
TDAPDLPEENQAR /2+/y7
LOD: 0.38 fmol/ug
LOQ: 1.14 fmol/ug
C7
AASGTQNNVLR /2+/ y6
LOD: 0.52 fmol/ug
LOQ: 1.57 fmol/ug
CD14
FPAIQNLALR /2+/y6
LOD: 5.96 fmol/ug
LOQ: 17.88 fmol/ug
CLU
ASSIIDELFQDR /2+/y8
LOD: 0.06 fmol/ug
LOQ: 0.19 fmol/ug
FN1
FLATTPNSLLVSWQPPR /2+/y12
LOD: 1.31 fmol/ug
LOQ: 3.93 fmol/ug
GSN
GGVASGFK /2+/y5
LOD: 4.15 fmol/ug
LOQ: 12.46 fmol/ug
ITIH2
VQSTITSR /2+/ y6
LOD: 0.19 fmol/ug
LOQ: 0.59 fmol/ug
KRT1
AQYEDIAQK /2+/y7
LOD: 1.28 fmol/ug
LOQ: 3.85 fmol/ug
SERPINF1
LSYEGEVTK /2+/y6
LOD: 0.80 fmol/ug
LOQ: 2.41 fmol/ug
VTN
DVWGIEGPIDAAFTR /2+/y9
LOD: 0.32 fmol/ug
LOQ: 0.96 fmol/ug
Heavy/light ratio
Fmol/ug

Supplementary Fig. 5

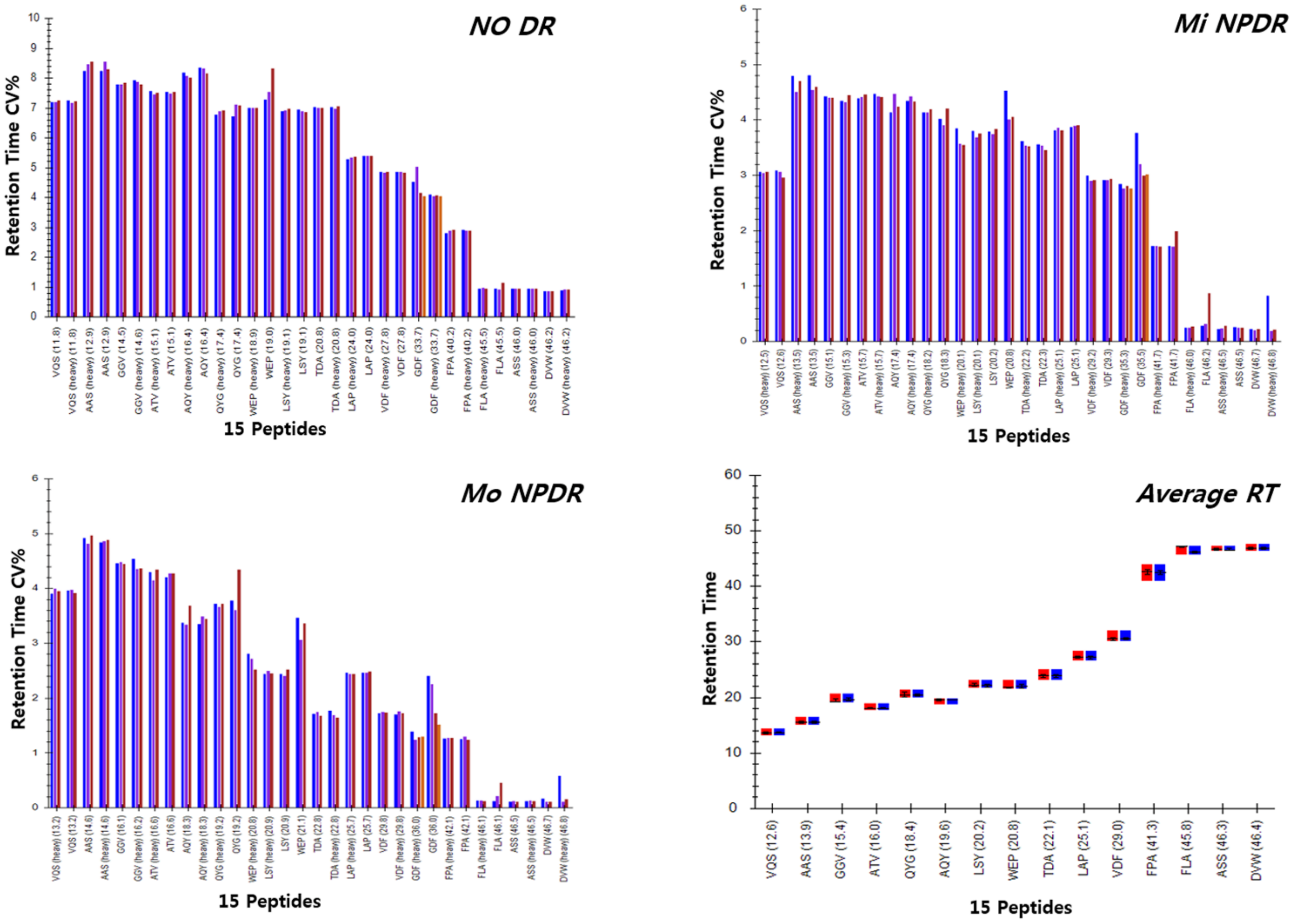

Supplementary Fig. 6

A

Coefficients[a]

Model		Unstandardized Coefficients		Standardized Coefficients	t	Sig.	Collinearity Statistics	
		B	Std. Error	Beta			Tolerance	VIF
1	(Constant)	.449	.212		2.121	.043		
	APOA4	.003	.001	.251	1.946	.062	.307	3.261
	APOH	.002	.001	.313	1.626	.115	.138	7.270
	B3GNT1	-.002	.005	-.073	-.482	.634	.224	4.466
	C4B	.000	.001	.040	.321	.751	.324	3.089
	C5	-.001	.001	-.089	-.723	.475	.338	2.962
	C7	-.031	.012	-.482	-2.666	.013	.156	6.394
	CD14	-.048	.038	-.146	-1.272	.214	.387	2.586
	CLU	.006	.002	.330	2.841	.008	.379	2.642
	GSN	-.012	.007	-.236	-1.622	.116	.242	4.134
	ITIH2	-.007	.005	-.219	-1.475	.151	.233	4.300
	KRT1	-.010	.009	-.103	-1.159	.256	.651	1.537

a. Dependent Variable: Group

B

Variables in the Analysis

Step		Tolerance	F to Remove	Wilks' Lambda
1	ITIH2	1.000	40.152	
2	ITIH2	.857	51.548	.828
	APOA4	.857	15.026	.486
3	ITIH2	.727	9.275	.347
	APOA4	.676	24.066	.460
	C7	.583	9.130	.346
4	ITIH2	.727	6.326	.233
	APOA4	.626	5.597	.229
	C7	.406	20.886	.315
	CLU	.452	13.928	.276

Variables Not in the Analysis

Step		Tolerance	Min. Tolerance	F to Enter	Wilks' Lambda
0	APOA4	1.000	1.000	7.918	.828
	APOH	1.000	1.000	6.952	.845
	B3GNT1	1.000	1.000	16.692	.695
	C4B	1.000	1.000	8.642	.815
	C5	1.000	1.000	16.639	.695
	C7	1.000	1.000	25.408	.599
	CD14	1.000	1.000	7.586	.834
	CLU	1.000	1.000	6.846	.847
	GSN	1.000	1.000	12.836	.748
	ITIH2	1.000	1.000	40.152	.486
	KRT1	1.000	1.000	5.106	.882
1	APOA4	.857	.857	15.026	.346
	APOH	.537	.537	2.470	.456
	B3GNT1	.755	.755	.563	.479
	C4B	.927	.927	.769	.476
	C5	.853	.853	1.504	.467
	C7	.739	.739	2.089	.460
	CD14	.942	.942	.759	.476
	CLU	.843	.843	14.740	.348
	GSN	.716	.716	.028	.486
	KRT1	.967	.967	.599	.478
2	APOH	.357	.357	.477	.341
	B3GNT1	.639	.639	4.199	.310
	C4B	.890	.822	1.964	.328
	C5	.740	.740	5.547	.300
	C7	.583	.583	9.130	.276
	CD14	.785	.714	4.988	.304
	CLU	.648	.648	3.511	.315
	GSN	.592	.592	2.654	.322
	KRT1	.889	.788	2.637	.322
3	APOH	.220	.220	1.735	.263
	B3GNT1	.428	.391	.114	.275
	C4B	.591	.388	.142	.275
	C5	.687	.542	1.997	.261
	CD14	.751	.539	6.682	.232
	CLU	.452	.406	13.928	.197
	GSN	.507	.499	.209	.274
	KRT1	.851	.559	.812	.270
4	APOH	.220	.220	1.670	.188
	B3GNT1	.396	.338	1.408	.189
	C4B	.549	.335	.285	.196
	C5	.670	.398	2.845	.182
	CD14	.739	.402	3.159	.181
	GSN	.504	.373	.386	.195
	KRT1	.829	.403	1.612	.188

IBM SPSS Statistics (IBM Inc., USA, version 20)

- We have performed stepwise MANOVA to determine more reliable candidates for constructing a multi-marker panel.

- **ITIH2**, **APOA4**, **C7**, and **CLU** are finally selected in analysis of stepwise MANOVA

Supplementary Fig. 7

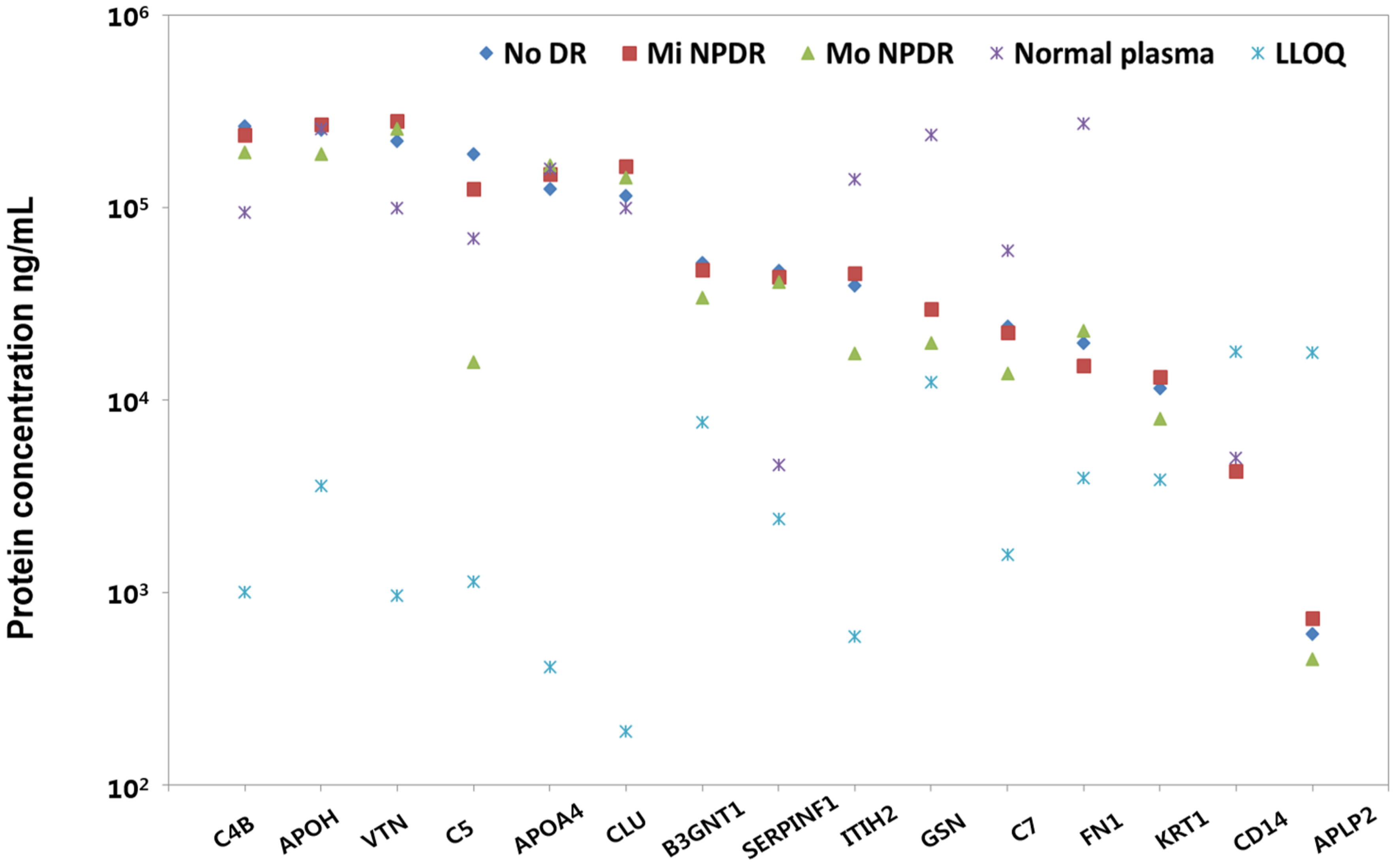

No DR
Mi NPDR
Mo NPDR
Normal plasma
LLOQ
Protein concentration ng/mL
10^6
10^5
10^4
10^3
10^2
C4B
APOH
VTN
C5
APOA4
CLU
B3GNT1
SERPINF1
ITIH2
GSN
C7
FN1
KRT1
CD14
APLP2
