# Supplementary material for: Development of Diagnostic Biomarkers for Detecting Diabetic Retinopathy at Early Stages Using Quantitative Proteomics
Source: J Diabetes Res. 2015 Nov 9;2016:6571976. doi: 10.1155/2016/6571976 (PMC4657408; doi:10.1155/2016/6571976)
Supplement: Supplementary file 1 — For identification of protein, an enhanced mass scan (EMS) ranging from 400 to 1200 m/z (scan speed, 4000 Da/s) was performed as an first MRM step, and an enhanced product ion (EPI) scan was then carried out to obtain MS/MS spectra. A MASCOT search was performed using the MS/MS spectra to identify the protein. Mascot.dll 1.6b23 and ABSciex.DataAccess.Wiff File DataReader.dll were used for importing data to Mascot and SwissProt 57.12 was used for database (513877 entries). The other database search parameters used for searching were the following: enzyme trypsin, 1 missed cleavage, variable carbamidomethylation modifications (C), oxidation (M), peptide tolerance 0.15 Da, MS/MS tolerance 0.1 Da, peptide charge 1+, and monoisotopic. Only significant hits at a p<0.05, as defined by MASCOT probability analysis, were accepted. [file 6571976.f1.zip › Supplementary Methods.pdf]

Supplementary Methods

Vitreous collection

For the diagnosis of PDR and NPDR, full ophthalmological evaluation which includes slit lamp biomicrosopic exam, fundus photographs, and fluorescein angiographs was performed. 0.5-0.8ml undiluted vitreous samples were collected by vitreous cutter into a 1.0-mL syringe and moved into tubes and then stored at -70˚C. For profiling the proteome of DR vitreous, we have collected undiluted vitreous of 29 eyes of 29 NPDR patients and 42 eyes of 42 PDR patients during vitrectomy as mentioned previously in our study [1]. The vitreous of eyes with idiopathic macular hole (MH) were used as non-diabetic control sample where we have collected vitreous of 32 eyes of non-diabetic MH patients. All patients provided informed consent before being enrolled in this study, in accordance with the protocol that was approved by the Institutional Review Board at Seoul National University Hospital (IRB No: H-0501-141-002).

MH, NPDR, and PDR samples were filtered/centrifuged at 15,000 *g* using 0.22 mm GV Durapore filter (Millipore, Carrigtwohill, Cork, Ireland) until all sample loaded passed completely through the filter. Protein concentrations were measured with the Pierce BCA reagent. After collecting these clarified (filtered/centrifuged) vitreous samples from MH, NPDR and PDR patients, 200 μl of individual samples from MH, NPDR and PDR patients were respectively pooled for LC-MS/MS experiments.

Strong cation exchange chromatographic fractionation

Digested vitreous samples were fractionated using strong cation exchange (SCX) chromatography, as follows. Digested and dried samples were reconstituted in 1ml of buffer A (25%

v/v acetonitrile (ACN) and 5 mM ammonium formate, adjusted to pH 2.7 with formic acid) and loaded onto a PolySULFOETHYL A column (PolyLC, Columbia, MD; 4.6 × 200 mm, 5-μm particle size, 200-Å pore size) on a LD 20AD system (Shimadzu, Japan). The column was equilibrated for 10 min in buffer A, and the peptides were eluted using a gradient of 0% to 30% buffer B [25% v/v ACN and 1 M ammonium formate (pH 3) with formic acid] over 60 min and 30% to 90% buffer B for 20 min at a flow rate of 0.7 ml/min. Absorbance was monitored at 280 nm, and the fractions were collected every 2 min after injection.

LC-ESI-MS/MS analysis

Vitreous proteins were analyzed by LC-MS/MS on an EasyLC (Proxeon Biosystems, Odense, Denmark, part of Thermo Fisher Scientific) that was coupled with a high-throughput tandem mass spectrometer (LTQ velos, Thermo, Waltham, MA), equipped with a nanoelectrospray device and fitted with a 10-μm fused silica emitter tip (New Objective, Woburn, MA, USA). In order to generate the Nanoflow LC gradient, solvent A (0.1% formic acid and 2% acetonitrile in ddH_2O) and solvent B (98% acetonitrile with 0.1% formic acid) were used (0% to 40% solvent B over 70 min, followed by a gradient of 40% to 90% for 10 min and 90% to 100% over 5 min) and 300 nL/min was used as the flow rate.

The spray voltage was set by 1.8 kV in the positive ion mode, and the temperature of heated capillary was 200°C. For MS2, Top 10 was selected. A cycle of 1 full-scan MS survey spectra (m/z 300–1800) was acquired in the profile mode. Fragmentation of the precursor and detection of the product ions occurred in the linear trap in a data-dependent manner for the top 10. For the MS/MS fragmentation, Only MS precursor that exceeded a threshold of 1000 ion counts was allowed to trigger.

All MS/MS spectra were acquired using the following parameters: normalized collision energy, 35; ion selection threshold, 1000 counts; activation time, 10 ms; and activation Q, 0.25. Dynamic exclusion was used with a repeat count of 1, a 15-s repeat duration, an exclusion list size of 50, an exclusion duration of 30 s, and ±1.5 m/z exclusion mass width. Instruments were controlled by Tune 2.6.0 and Xcalibur 2.1.

Criteria for protein identification and label free quantitation

All MS/MS spectra were analyzed using SEQUEST algorithm and Uniprot human 2013.12 fasta as a human database was used. Sequest parameters for identification and quantitation were set by fragment ion mass tolerance of 1.00 Da, a parent ion tolerance of 1.5 Da, carbamidomethyl of cystein as a fixed modification, and Oxidation of methionine as a variable modification.

Database searches were performed using Proteome Discoverer software (Thermo Fischer Scientific, ver 1.3). The MS/MS data were queried against the human protein database (Uniprot human. 2013.12). The searches were performed using the following parameters: MS accuracy, 1.5 Da ; MS/MS accuracy, 1.0 Da for CID; trypsin digestion with two missed cleavages, allowed; fixed carbamidomethyl modification of cysteine, +57.0215 Da; and variable modification of oxidized methionine, +15.9949 Da. The number of peptides and proteins in the protein groups were estimated using Proteome Discoverer. False discovery rates was set at < 1% , where PepDistiller was used to calculate the probability value (q-value) to maintain the FDR measured by the decoy hits at <1% for every peptide-spectrum match (PSM).

For the label free quantitation, summation of the spectral counts, validation of MS/MS-based peptide and protein identification, and annotation of peptide into proteins were carried out using the Proteome Discoverer software. Using the selection criteria of more than two unique peptides (>99%) contained, and SEQUEST parameter (deltaCn scores is greater than 0.10) in MH versus NPDR, and MH versus PDR, proteins were quantitated in the three replicated experiments (Protein-level FDR was

calculated at 1%). The spectral count result was reported by Proteome Discoverer software which provide an estimate of relative values of protein abundance in MH versus NPDR, and MH versus PDR.

For the reliable normalization among sample runs, a normalized spectral abundance factor (NSAF) method was used [2]. NSAF is defined as follows; NSAF = (SpC/Mw)/Σ(SpC/Mw)n, where ″SpC″ represent Spectral Counts, ″Mw″ represent protein molecular weight in kDa, and ″N″ represent Total number of proteins. Also, high-confidence proteins for label-free quantitation were selected with sum spectral count ≥ 10 in datasets. Through the NSAF method, we were able to achieve the comparison of abundance of individual proteins in multiple independent samples and have been applied to quantify the expression changes in various complexes

Q-Exactive analysis

To collect DR-specific MS/MS spectra to build a DR spectral library, a DR sample, which consisted of pooled plasma (1 μg/μL) and a 15 SIS peptide mixture (50 fmol/μL), was analyzed by online nanoflow liquid chromatography tandem mass spectrometry (LC− MS/MS) on a Q-Exactive quadrupole orbitrap mass spectrometer. Briefly, nanoLC− MS/MS-experiments were performed on an EASY-nLC 1000 system (Thermo Scientific, Odense, Denmark) connected to a Q-Exactive spectrometer (Thermo Scientific, Bremen, Germany) through a nanoelectrospray ion source. Peptides were loaded by an autosampler onto a precolumn (2 cm long; ID, 75 μm; particle size, 3 μm) and an analytical column (15 cm long; ID, 50 μm; particle size, 2 μm) packed with reversed-phase C18. The sample (plasma peptides and 15 SIS peptides) were loaded at a flow rate of 300 nL/min. Peptides were

separated on a linear ACN gradient from 3% to 40% for 20 min and 40% to 90% for 2 min. The total duration of acquisition was 65 min. However, peptides were only eluted between 3 and 35 min.

The Q-Exactive instrument was operated in the data dependent mode (DDA) to automatically switch between full scan MS and MS/MS modes of acquisition. A survey of full scan MS spectra (350– 1600 m/z) were acquired in the Orbitrap with 70,000 resolution (200 m/z) after the accumulation of ions to a 3×10^6 target value based on predictive AGC from the previous full scan. Dynamic exclusion was set to 30 s. The 10 most intense multiply charged ions ($z \geq 2$) were sequentially isolated and fragmented by higher-energy collisional dissociation (HCD) in an octopole collision cell with a fixed injection time of 60 ms, a AGC target value of 5×10^4 and a revolution of 17,500. Typical mass spectrometric conditions were as follows: S-lens RF level, 65; spray voltage, 2 kV; heated capillary temperature, 320°C; and normalized HCD collision energy, 30%. The under fill ratio, MS/MS ion selection threshold, and isolation width were set to 1%, 8.3×10^3 counts, and 2 m/z, respectively. The fixed first m/z was set to 100.

Database searches and the construction of a spectral library

Database searches were performed using Proteome Discoverer software (Thermo Fischer Scientific, ver 1.3). The MS/MS data were queried against the human protein database (Uniprot human. 2013.12). The searches were performed using the following parameters: MS accuracy, 10 ppm; MS/MS accuracy, 0.6 Da for HCD; trypsin digestion with two missed cleavages, allowed; fixed carbamidomethyl modification of cysteine, +57.0215 Da; and variable modification of oxidized methionine, +15.9949 Da, Lys ($6C^{13}$; $2N^{15}$), and Arg ($6C^{13}$; $4N^{15}$). The number of peptides and proteins in the protein groups were estimated using Proteome Discoverer. False discovery rates was

set at < 1% , where PepDistiller was used to calculate the probability value (q-value) to maintain the FDR measured by the decoy hits at <1% for every peptide-spectrum match (PSM).

A spectral library was built using Skyline software (ver 2.1) from the spectral data files that were searched with Sequest followed by PeptideProphet as part of the trans-proteomic pipeline (TPP). The mzXML files (MRM-triggered MS2 file) and pepXML files (the search engine out file) were imported into the Skyline tool and then we built the DM-specific spectral library, where the PeptideProphet cut-off score was set at 0.95.

Supplementary References

1. K. Kim, S.J. Kim, H.G. Yu, et al. "Verification of biomarkers for diabetic retinopathy by multiple reaction monitoring," *J Proteome Res, vol.* 9, no. 2, pp. 689-699.

2. H. Min, D. Han, Y. Kim, J.Y. Cho, J. Jin, and Y. Kim. "Label-free quantitative proteomics and N-terminal analysis of human metastatic lung cancer cells," *Mol Cells, vol.* 37, no. 6, pp. 457-466.
