# Supplementary material for: Development of Diagnostic Biomarkers for Detecting Diabetic Retinopathy at Early Stages Using Quantitative Proteomics
Source: J Diabetes Res. 2015 Nov 9;2016:6571976. doi: 10.1155/2016/6571976 (PMC4657408; doi:10.1155/2016/6571976)
Supplement: Supplementary file 1 — For identification of protein, an enhanced mass scan (EMS) ranging from 400 to 1200 m/z (scan speed, 4000 Da/s) was performed as an first MRM step, and an enhanced product ion (EPI) scan was then carried out to obtain MS/MS spectra. A MASCOT search was performed using the MS/MS spectra to identify the protein. Mascot.dll 1.6b23 and ABSciex.DataAccess.Wiff File DataReader.dll were used for importing data to Mascot and SwissProt 57.12 was used for database (513877 entries). The other database search parameters used for searching were the following: enzyme trypsin, 1 missed cleavage, variable carbamidomethylation modifications (C), oxidation (M), peptide tolerance 0.15 Da, MS/MS tolerance 0.1 Da, peptide charge 1+, and monoisotopic. Only significant hits at a p<0.05, as defined by MASCOT probability analysis, were accepted. [file 6571976.f1.zip › Supplementary Tables.pdf]

Supplementary Table 6. Selection of reliable transitions for MRM analysis

a) Uniprot human database (Uniprot Human.2013.12 fasta)

b) Ion charge state and character

c) Precusor ion m/z

d) Product ion m/z

e) declustering potential parameter value

f) collision energy parameter value

g) MASCOT search Identification from LC-MRM/MS (IDA mode)

N	Gene name	Accession Number [a)]	Peptide Sequence	Ion [b)]	Q1 [c)]	Q3 [d)]	DP [e)]
1	AZGP1	P25311	YSLTYIYTGLSK	+2y9	704.87	1045.56	82.5
2	AZGP1	P25311	YSLTYIYTGLSK	+2y8	704.87	944.51	82.5
3	AZGP1	P25311	YSLTYIYTGLSK	+2y7	704.87	781.45	82.5
4	AZGP1	P25311	YSLTYIYTGLSK	+2y6	704.87	668.36	82.5
5	AZGP1	P25311	YSLTYIYTGLSK	+2y5	704.87	505.30	82.5
6	AZGP1	P25311	DYIEFNK	+2y5	464.72	650.35	65
7	AZGP1	P25311	DYIEFNK	+2y4	464.72	537.27	65
8	AZGP1	P25311	DYIEFNK	+2y3	464.72	408.22	65
9	AZGP1	P25311	EIPAWVPFDPAAQITK	+2y11	891.97	1186.65	96.1
10	AZGP1	P25311	EIPAWVPFDPAAQITK	+2y10	891.97	1087.58	96.1
11	AZGP1	P25311	EIPAWVPFDPAAQITK	+2y7	891.97	728.43	96.1
12	AZGP1	P25311	EIPAWVPFDPAAQITK	+2y5	891.97	560.34	96.1
13	AZGP1	P25311	EIPAWVPFDPAAQITK	+2y4	891.97	489.30	96.1
14	AZGP1	P25311	WEAEPVYVQR	+2y9	638.82	1090.55	77.7
15	AZGP1	P25311	WEAEPVYVQR	+2y8	638.82	961.51	77.7
16	AZGP1	P25311	WEAEPVYVQR	+2y7	638.82	890.47	77.7
17	AZGP1	P25311	WEAEPVYVQR	+2y6	638.82	761.43	77.7
18	AZGP1	P25311	WEAEPVYVQR	+2y4	638.82	565.31	77.7
19	AZGP1	P25311	AGEVQEPELR	+2y8	564.29	999.51	72.3
20	AZGP1	P25311	AGEVQEPELR	+2y7	564.29	870.47	72.3
21	AZGP1	P25311	AGEVQEPELR	+2y6	564.29	771.40	72.3
22	AZGP1	P25311	AGEVQEPELR	+2y5	564.29	643.34	72.3
23	AZGP1	P25311	AGEVQEPELR	+2y4	564.29	514.30	72.3
24	APOA1	P02647	LLDNWDSVTSTFSK	+2y10	806.90	1157.55	89.9
25	APOA1	P02647	LLDNWDSVTSTFSK	+2y9	806.90	971.47	89.9
26	APOA1	P02647	LLDNWDSVTSTFSK	+2y8	806.90	856.44	89.9
27	APOA1	P02647	LLDNWDSVTSTFSK	+2y6	806.90	670.34	89.9
28	APOA1	P02647	LLDNWDSVTSTFSK	+2y5	806.90	569.29	89.9
29	APOA1	P02647	EQLGPVTQEFWDNLEK	+2y10	966.97	1309.61	101.6
30	APOA1	P02647	EQLGPVTQEFWDNLEK	+2y7	966.97	951.46	101.6
31	APOA1	P02647	EQLGPVTQEFWDNLEK	+2y6	966.97	804.39	101.6
32	APOA1	P02647	EQLGPVTQEFWDNLEK	+2y5	966.97	618.31	101.6
33	APOA1	P02647	EQLGPVTQEFWDNLEK	+2y4	966.97	503.28	101.6
34	APOA1	P02647	VQPYLDDFQK	+2y8	626.81	1025.49	76.8
35	APOA1	P02647	VQPYLDDFQK	+2y7	626.81	928.44	76.8
36	APOA1	P02647	VQPYLDDFQK	+2y6	626.81	765.38	76.8
37	APOA1	P02647	VQPYLDDFQK	+2y5	626.81	652.29	76.8

38	APOA1	P02647	VQPYLDDFQK	+2y4	626.81	537.27	76.8
39	APOA1	P02647	AELQEGAR	+2y7	437.22	802.41	63
40	APOA1	P02647	AELQEGAR	+2y6	437.22	673.36	63
41	APOA1	P02647	AELQEGAR	+2y5	437.22	560.28	63
42	APOA1	P02647	AELQEGAR	+2y4	437.22	432.22	63
43	APOA1	P02647	AELQEGAR	+2y3	437.22	303.18	63
44	APOA1	P02647	VSFLSALEEYTK	+2y9	693.86	1053.55	81.7
45	APOA1	P02647	VSFLSALEEYTK	+2y8	693.86	940.46	81.7
46	APOA1	P02647	VSFLSALEEYTK	+2y7	693.86	853.43	81.7
47	APOA1	P02647	VSFLSALEEYTK	+2y6	693.86	782.39	81.7
48	APOA1	P02647	VSFLSALEEYTK	+2y4	693.86	540.27	81.7
49	CTSD	P07339	YSQAVPAVTEGPIPEVLK	+2y13	949.52	1349.77	100.3
50	CTSD	P07339	YSQAVPAVTEGPIPEVLK	+2y10	949.52	1082.61	100.3
51	CTSD	P07339	YSQAVPAVTEGPIPEVLK	+2y8	949.52	852.52	100.3
52	CTSD	P07339	YSQAVPAVTEGPIPEVLK	+2y7	949.52	795.50	100.3
53	CTSD	P07339	YSQAVPAVTEGPIPEVLK	+2y5	949.52	585.36	100.3
54	CTSD	P07339	QVFGEATK	+2y7	440.23	751.40	63.2
55	CTSD	P07339	QVFGEATK	+2y6	440.23	652.33	63.2
56	CTSD	P07339	QVFGEATK	+2y5	440.23	505.26	63.2
57	CTSD	P07339	QVFGEATK	+2y4	440.23	448.24	63.2
58	CTSD	P07339	QVFGEATK	+2y3	440.23	319.20	63.2
59	CTSD	P07339	QPGITFIAAK	+2y8	523.31	820.49	69.3
60	CTSD	P07339	QPGITFIAAK	+2y7	523.31	763.47	69.3
61	CTSD	P07339	QPGITFIAAK	+2y6	523.31	650.39	69.3
62	CTSD	P07339	QPGITFIAAK	+2y5	523.31	549.34	69.3
63	CTSD	P07339	QPGITFIAAK	+2y4	523.31	402.27	69.3
64	CTSD	P07339	GSLSYLNVTR	+2y7	555.30	852.46	71.6
65	CTSD	P07339	GSLSYLNVTR	+2y6	555.30	765.43	71.6
66	CTSD	P07339	GSLSYLNVTR	+2y5	555.30	602.36	71.6
67	CTSD	P07339	GSLSYLNVTR	+2y4	555.30	489.28	71.6
68	CTSD	P07339	GSLSYLNVTR	+2y3	555.30	375.24	71.6
69	CTSD	P07339	VGFAEAAR	+2y7	410.72	721.36	61.1
70	CTSD	P07339	VGFAEAAR	+2y6	410.72	664.34	61.1
71	CTSD	P07339	VGFAEAAR	+2y5	410.72	517.27	61.1
72	CTSD	P07339	VGFAEAAR	+2y4	410.72	446.24	61.1
73	CTSD	P07339	VGFAEAAR	+2y3	410.72	317.19	61.1
74	A1BG	P04217	SGLSTGWTQLSK	+2y9	632.83	1007.52	77.3
75	A1BG	P04217	SGLSTGWTQLSK	+2y8	632.83	920.48	77.3
76	A1BG	P04217	SGLSTGWTQLSK	+2y7	632.83	819.44	77.3
77	A1BG	P04217	SGLSTGWTQLSK	+2y6	632.83	762.41	77.3
78	A1BG	P04217	SGLSTGWTQLSK	+2y5	632.83	576.34	77.3
79	A1BG	P04217	LLELTGPK	+2y7	435.77	757.45	62.9
80	A1BG	P04217	LLELTGPK	+2y6	435.77	644.36	62.9
81	A1BG	P04217	LLELTGPK	+2y5	435.77	515.32	62.9
82	A1BG	P04217	LLELTGPK	+2y4	435.77	402.23	62.9
83	A1BG	P04217	LLELTGPK	+2y3	435.77	301.19	62.9
84	A1BG	P04217	GVTFLLR	+2y5	403.25	649.40	60.5
85	A1BG	P04217	GVTFLLR	+2y4	403.25	548.36	60.5
86	A1BG	P04217	GVTFLLR	+2y3	403.25	401.29	60.5
87	A1BG	P04217	ATWSGAVLAGR	+2y9	544.80	916.50	70.8

88	A1BG	P04217	ATWSGAVLAGR	+2y8	544.80	730.42	70.8
89	A1BG	P04217	ATWSGAVLAGR	+2y7	544.80	643.39	70.8
90	A1BG	P04217	ATWSGAVLAGR	+2y6	544.80	586.37	70.8
91	A1BG	P04217	ATWSGAVLAGR	+2y4	544.80	416.26	70.8
92	APOH	P02749	ATVVYQGER	+2y7	511.77	850.44	68.4
93	APOH	P02749	ATVVYQGER	+2y6	511.77	751.37	68.4
94	APOH	P02749	ATVVYQGER	+2y5	511.77	652.30	68.4
95	APOH	P02749	ATVVYQGER	+2y4	511.77	489.24	68.4
96	APOH	P02749	ATVVYQGER	+2y3	511.77	361.18	68.4
97	SERPINF1	P36955	LAAAVSNFGYDLYR	+2y10	780.40	1233.59	88
98	SERPINF1	P36955	LAAAVSNFGYDLYR	+2y9	780.40	1134.52	88
99	SERPINF1	P36955	LAAAVSNFGYDLYR	+2y8	780.40	1047.49	88
100	SERPINF1	P36955	LAAAVSNFGYDLYR	+2y6	780.40	786.38	88
101	SERPINF1	P36955	LAAAVSNFGYDLYR	+2y3	780.40	451.27	88
102	SERPINF1	P36955	ELLDTVTAPQK	+2y9	607.83	972.54	75.4
103	SERPINF1	P36955	ELLDTVTAPQK	+2y8	607.83	859.45	75.4
104	SERPINF1	P36955	ELLDTVTAPQK	+2y7	607.83	744.43	75.4
105	SERPINF1	P36955	ELLDTVTAPQK	+2y5	607.83	544.31	75.4
106	SERPINF1	P36955	ELLDTVTAPQK	+2y3	607.83	372.22	75.4
107	SERPINF1	P36955	SSFVAPLEK	+2y7	489.27	803.47	66.8
108	SERPINF1	P36955	SSFVAPLEK	+2y6	489.27	656.40	66.8
109	SERPINF1	P36955	SSFVAPLEK	+2y5	489.27	557.33	66.8
110	SERPINF1	P36955	SSFVAPLEK	+2y4	489.27	486.29	66.8
111	SERPINF1	P36955	SSFVAPLEK	+2y3	489.27	389.24	66.8
112	SERPINF1	P36955	LSYEGEVTK	+2y8	513.26	912.43	68.5
113	SERPINF1	P36955	LSYEGEVTK	+2y7	513.26	825.40	68.5
114	SERPINF1	P36955	LSYEGEVTK	+2y6	513.26	662.34	68.5
115	SERPINF1	P36955	LSYEGEVTK	+2y5	513.26	533.29	68.5
116	SERPINF1	P36955	LSYEGEVTK	+2y3	513.26	347.23	68.5
117	SERPINF1	P36955	DTDTGALLFIGK	+2y10	625.83	1034.59	76.7
118	SERPINF1	P36955	DTDTGALLFIGK	+2y8	625.83	818.51	76.7
119	SERPINF1	P36955	DTDTGALLFIGK	+2y6	625.83	690.45	76.7
120	SERPINF1	P36955	DTDTGALLFIGK	+2y4	625.83	464.29	76.7
121	SERPINF1	P36955	DTDTGALLFIGK	+2y3	625.83	317.22	76.7
122	A2M	P01023	NEDSLVFVQTDK	+2y7	697.84	836.45	82
123	A2M	P01023	NEDSLVFVQTDK	+2y6	697.84	737.38	82
124	A2M	P01023	NEDSLVFVQTDK	+2y5	697.84	590.31	82
125	A2M	P01023	NEDSLVFVQTDK	+2y4	697.84	491.25	82
126	A2M	P01023	NEDSLVFVQTDK	+2y3	697.84	363.19	82
127	A2M	P01023	IAQWQSFQLEGGLK	+2y9	802.93	978.53	89.7
128	A2M	P01023	IAQWQSFQLEGGLK	+2y7	802.93	744.43	89.7
129	A2M	P01023	IAQWQSFQLEGGLK	+2y6	802.93	616.37	89.7
130	A2M	P01023	IAQWQSFQLEGGLK	+2y5	802.93	503.28	89.7
131	A2M	P01023	IAQWQSFQLEGGLK	+2y4	802.93	374.24	89.7
132	A2M	P01023	QFSFPLSSEPFQGSYK	+2y12	924.94	1339.65	98.5
133	A2M	P01023	QFSFPLSSEPFQGSYK	+2y10	924.94	1129.52	98.5
134	A2M	P01023	QFSFPLSSEPFQGSYK	+2y9	924.94	1042.48	98.5
135	A2M	P01023	QFSFPLSSEPFQGSYK	+2y7	924.94	826.41	98.5
136	A2M	P01023	QFSFPLSSEPFQGSYK	+2y4	924.94	454.23	98.5
137	A2M	P01023	ALLAYAFALAGNQDK	+2y10	783.42	1034.53	88.2

138	A2M	P01023	ALLAYAFALAGNQDK	+2y9	783.42	963.49	88.2
139	A2M	P01023	ALLAYAFALAGNQDK	+2y8	783.42	816.42	88.2
140	A2M	P01023	ALLAYAFALAGNQDK	+2y6	783.42	632.30	88.2
141	A2M	P01023	ALLAYAFALAGNQDK	+2y5	783.42	561.26	88.2
142	A2M	P01023	VSNQTLSLFFTVLQDVPVR	+2y11	1082.09	1320.73	110
143	A2M	P01023	VSNQTLSLFFTVLQDVPVR	+2y10	1082.09	1173.66	110
144	A2M	P01023	VSNQTLSLFFTVLQDVPVR	+2y7	1082.09	826.48	110
145	A2M	P01023	VSNQTLSLFFTVLQDVPVR	+2y6	1082.09	713.39	110
146	A2M	P01023	VSNQTLSLFFTVLQDVPVR	+2y3	1082.09	371.24	110
147	SERPINA3	P01011	EQLSLLDR	+2y6	487.27	716.43	66.6
148	SERPINA3	P01011	EQLSLLDR	+2y5	487.27	603.35	66.6
149	SERPINA3	P01011	EQLSLLDR	+2y4	487.27	516.31	66.6
150	SERPINA3	P01011	EQLSLLDR	+2y3	487.27	403.23	66.6
151	SERPINA3	P01011	EIGELYLPK	+2y7	531.30	819.46	69.8
152	SERPINA3	P01011	EIGELYLPK	+2y6	531.30	762.44	69.8
153	SERPINA3	P01011	EIGELYLPK	+2y5	531.30	633.40	69.8
154	SERPINA3	P01011	EIGELYLPK	+2y4	531.30	520.31	69.8
155	SERPINA3	P01011	EIGELYLPK	+2y3	531.30	357.25	69.8
156	SERPINA3	P01011	DYNLNDILLQLGIEEAFTSK	+2y10	1148.59	1094.57	114.8
157	SERPINA3	P01011	DYNLNDILLQLGIEEAFTSK	+2y9	1148.59	981.49	114.8
158	SERPINA3	P01011	DYNLNDILLQLGIEEAFTSK	+2y5	1148.59	553.30	114.8
159	SERPINA3	P01011	DYNLNDILLQLGIEEAFTSK	+2y4	1148.59	482.26	114.8
160	SERPINA3	P01011	DYNLNDILLQLGIEEAFTSK	+2y3	1148.59	335.19	114.8
161	SERPINA3	P01011	AVLDVFEEGTEASAATAVK	+2y12	954.48	1134.56	100.7
162	SERPINA3	P01011	AVLDVFEEGTEASAATAVK	+2y11	954.48	1005.52	100.7
163	SERPINA3	P01011	AVLDVFEEGTEASAATAVK	+2y6	954.48	560.34	100.7
164	SERPINA3	P01011	AVLDVFEEGTEASAATAVK	+2y5	954.48	489.30	100.7
165	SERPINA3	P01011	AVLDVFEEGTEASAATAVK	+2y4	954.48	418.27	100.7
166	SERPINA3	P01011	ITLLSALVETR	+2y8	608.37	888.51	75.5
167	SERPINA3	P01011	ITLLSALVETR	+2y7	608.37	775.43	75.5
168	SERPINA3	P01011	ITLLSALVETR	+2y6	608.37	688.40	75.5
169	SERPINA3	P01011	ITLLSALVETR	+2y5	608.37	617.36	75.5
170	SERPINA3	P01011	ITLLSALVETR	+2y4	608.37	504.28	75.5
171	APOA4	P06727	SELTQQLNALFQDK	+2y8	817.92	948.51	90.7
172	APOA4	P06727	SELTQQLNALFQDK	+2y7	817.92	835.43	90.7
173	APOA4	P06727	SELTQQLNALFQDK	+2y5	817.92	650.35	90.7
174	APOA4	P06727	SELTQQLNALFQDK	+2y4	817.92	537.27	90.7
175	APOA4	P06727	SELTQQLNALFQDK	+2y3	817.92	390.20	90.7
176	APOA4	P06727	LGEVNTYAGDLQK	+2y9	704.36	1009.49	82.5
177	APOA4	P06727	LGEVNTYAGDLQK	+2y8	704.36	895.45	82.5
178	APOA4	P06727	LGEVNTYAGDLQK	+2y7	704.36	794.40	82.5
179	APOA4	P06727	LGEVNTYAGDLQK	+2y6	704.36	631.34	82.5
180	APOA4	P06727	LGEVNTYAGDLQK	+2y5	704.36	560.30	82.5
181	APOA4	P06727	TQVNTQAEQLR	+2y9	644.34	1058.56	78.1
182	APOA4	P06727	TQVNTQAEQLR	+2y8	644.34	959.49	78.1
183	APOA4	P06727	TQVNTQAEQLR	+2y7	644.34	845.45	78.1
184	APOA4	P06727	TQVNTQAEQLR	+2y6	644.34	744.40	78.1
185	APOA4	P06727	TQVNTQAEQLR	+2y5	644.34	616.34	78.1
186	APOA4	P06727	IDQNVEELK	+2y8	544.29	974.48	70.8
187	APOA4	P06727	IDQNVEELK	+2y7	544.29	859.45	70.8

188	APOA4	P06727	IDQNVEELK	+2y6	544.29	731.39	70.8
189	APOA4	P06727	IDQNVEELK	+2y4	544.29	518.28	70.8
190	APOA4	P06727	IDQNVEELK	+2y3	544.29	389.24	70.8
191	APOA4	P06727	LAPLAEDVR	+2y7	492.28	799.43	67
192	APOA4	P06727	LAPLAEDVR	+2y6	492.28	702.38	67
193	APOA4	P06727	LAPLAEDVR	+2y5	492.28	589.29	67
194	APOA4	P06727	LAPLAEDVR	+2y4	492.28	518.26	67
195	APOA4	P06727	LAPLAEDVR	+2y3	492.28	389.21	67
196	C3	P01024	VVLVSLQSGYLFIQTDK	+2y10	955.54	1171.60	100.8
197	C3	P01024	VVLVSLQSGYLFIQTDK	+2y9	955.54	1084.57	100.8
198	C3	P01024	VVLVSLQSGYLFIQTDK	+2y7	955.54	864.48	100.8
199	C3	P01024	VVLVSLQSGYLFIQTDK	+2y6	955.54	751.40	100.8
200	C3	P01024	VVLVSLQSGYLFIQTDK	+2y5	955.54	604.33	100.8
201	C3	P01024	AYYENSPQQVFSTEFEVK	+2y8	1083.50	986.48	110.1
202	C3	P01024	AYYENSPQQVFSTEFEVK	+2y7	1083.50	839.41	110.1
203	C3	P01024	AYYENSPQQVFSTEFEVK	+2y6	1083.50	752.38	110.1
204	C3	P01024	AYYENSPQQVFSTEFEVK	+2y4	1083.50	522.29	110.1
205	C3	P01024	AYYENSPQQVFSTEFEVK	+2y3	1083.50	375.22	110.1
206	C3	P01024	TGLQEVEVK	+2y8	501.78	901.50	67.7
207	C3	P01024	TGLQEVEVK	+2y6	501.78	731.39	67.7
208	C3	P01024	TGLQEVEVK	+2y5	501.78	603.33	67.7
209	C3	P01024	TGLQEVEVK	+2y4	501.78	474.29	67.7
210	C3	P01024	TGLQEVEVK	+2y3	501.78	375.22	67.7
211	C3	P01024	APSTWLTAYVVK	+2y10	668.37	1167.64	79.8
212	C3	P01024	APSTWLTAYVVK	+2y9	668.37	1080.61	79.8
213	C3	P01024	APSTWLTAYVVK	+2y8	668.37	979.56	79.8
214	C3	P01024	APSTWLTAYVVK	+2y7	668.37	793.48	79.8
215	C3	P01024	APSTWLTAYVVK	+2y6	668.37	680.40	79.8
216	C3	P01024	QLYNVEATSYALLALLQLK	+2y12	1076.11	1333.81	109.6
217	C3	P01024	QLYNVEATSYALLALLQLK	+2y11	1076.11	1232.76	109.6
218	C3	P01024	QLYNVEATSYALLALLQLK	+2y10	1076.11	1145.73	109.6
219	C3	P01024	QLYNVEATSYALLALLQLK	+2y9	1076.11	982.67	109.6
220	C3	P01024	QLYNVEATSYALLALLQLK	+2y6	1076.11	685.46	109.6
221	FGG	P02679	YLQEIYNSNNQK	+2y9	757.37	1109.52	86.3
222	FGG	P02679	YLQEIYNSNNQK	+2y7	757.37	867.40	86.3
223	FGG	P02679	YLQEIYNSNNQK	+2y6	757.37	704.33	86.3
224	FGG	P02679	YLQEIYNSNNQK	+2y5	757.37	590.29	86.3
225	FGG	P02679	YLQEIYNSNNQK	+2y4	757.37	503.26	86.3
226	FGG	P02679	QSGLYFIKPLK	+2y7	647.38	908.56	78.3
227	FGG	P02679	QSGLYFIKPLK	+2y6	647.38	745.50	78.3
228	FGG	P02679	QSGLYFIKPLK	+2y5	647.38	598.43	78.3
229	FGG	P02679	QSGLYFIKPLK	+2y4	647.38	485.34	78.3
230	FGG	P02679	QSGLYFIKPLK	+2y3	647.38	357.25	78.3
231	FGG	P02679	LDGSVDFK	+2y7	440.72	767.36	63.2
232	FGG	P02679	LDGSVDFK	+2y6	440.72	652.33	63.2
233	FGG	P02679	LDGSVDFK	+2y5	440.72	595.31	63.2
234	FGG	P02679	LDGSVDFK	+2y4	440.72	508.28	63.2
235	FGG	P02679	LDGSVDFK	+2y3	440.72	409.21	63.2
236	FGG	P02679	VELEDWNGR	+2y7	559.27	889.42	71.9
237	FGG	P02679	VELEDWNGR	+2y6	559.27	776.33	71.9

238	FGG	P02679	VELEDWNGR	+2y5	559.27	647.29	71.9
239	FGG	P02679	VELEDWNGR	+2y4	559.27	532.26	71.9
240	FGG	P02679	VELEDWNGR	+2y3	559.27	346.18	71.9
241	FGG	P02679	ASTPNGYDNGIIWATWK	+2y6	947.46	804.44	100.2
242	FGG	P02679	ASTPNGYDNGIIWATWK	+2y5	947.46	691.36	100.2
243	FGG	P02679	ASTPNGYDNGIIWATWK	+2y4	947.46	505.28	100.2
244	FGG	P02679	ASTPNGYDNGIIWATWK	+2y3	947.46	434.24	100.2
245	FGG	P02679	ASTPNGYDNGIIWATWK	+2y2	947.46	333.19	100.2
246	HPX	P02790	NFPSPVDAAFR	+2y9	610.81	959.49	75.6
247	HPX	P02790	NFPSPVDAAFR	+2y8	610.81	862.44	75.6
248	HPX	P02790	NFPSPVDAAFR	+2y7	610.81	775.41	75.6
249	HPX	P02790	NFPSPVDAAFR	+2y5	610.81	579.29	75.6
250	HPX	P02790	NFPSPVDAAFR	+2y4	610.81	464.26	75.6
251	HPX	P02790	VWVYPPEK	+2y7	509.27	918.47	68.2
252	HPX	P02790	VWVYPPEK	+2y6	509.27	732.39	68.2
253	HPX	P02790	VWVYPPEK	+2y5	509.27	633.32	68.2
254	HPX	P02790	VWVYPPEK	+2y4	509.27	470.26	68.2
255	HPX	P02790	VWVYPPEK	+2y3	509.27	373.21	68.2
256	HPX	P02790	LYLVQGTQVYVFLTK	+2y12	886.50	1382.77	95.7
257	HPX	P02790	LYLVQGTQVYVFLTK	+2y11	886.50	1283.70	95.7
258	HPX	P02790	LYLVQGTQVYVFLTK	+2y10	886.50	1155.64	95.7
259	HPX	P02790	LYLVQGTQVYVFLTK	+2y6	886.50	770.44	95.7
260	HPX	P02790	LYLVQGTQVYVFLTK	+2y4	886.50	508.31	95.7
261	HPX	P02790	GGYTLVSGYPK	+2y8	571.30	864.48	72.8
262	HPX	P02790	GGYTLVSGYPK	+2y7	571.30	763.43	72.8
263	HPX	P02790	GGYTLVSGYPK	+2y6	571.30	650.35	72.8
264	HPX	P02790	GGYTLVSGYPK	+2y5	571.30	551.28	72.8
265	HPX	P02790	GGYTLVSGYPK	+2y4	571.30	464.25	72.8
266	HPX	P02790	LWWLDLK	+2y6	487.28	860.47	66.6
267	HPX	P02790	LWWLDLK	+2y5	487.28	674.39	66.6
268	HPX	P02790	LWWLDLK	+2y4	487.28	488.31	66.6
269	HPX	P02790	LWWLDLK	+2y3	487.28	375.22	66.6
270	F2	P00734	TATSEYQTFFNPR	+2y8	781.37	1072.52	88.1
271	F2	P00734	TATSEYQTFFNPR	+2y6	781.37	781.40	88.1
272	F2	P00734	TATSEYQTFFNPR	+2y5	781.37	680.35	88.1
273	F2	P00734	TATSEYQTFFNPR	+2y4	781.37	533.28	88.1
274	F2	P00734	TATSEYQTFFNPR	+2y3	781.37	386.21	88.1
275	F2	P00734	ELLESYIDGR	+2y7	597.80	839.39	74.7
276	F2	P00734	ELLESYIDGR	+2y6	597.80	710.35	74.7
277	F2	P00734	ELLESYIDGR	+2y5	597.80	623.31	74.7
278	F2	P00734	ELLESYIDGR	+2y4	597.80	460.25	74.7
279	F2	P00734	ELLESYIDGR	+2y3	597.80	347.17	74.7
280	F2	P00734	ETAASLLQAGYK	+2y8	626.33	879.49	76.8
281	F2	P00734	ETAASLLQAGYK	+2y6	626.33	679.38	76.8
282	F2	P00734	ETAASLLQAGYK	+2y5	626.33	566.29	76.8
283	F2	P00734	ETAASLLQAGYK	+2y4	626.33	438.23	76.8
284	F2	P00734	ETAASLLQAGYK	+2y3	626.33	367.20	76.8
285	F2	P00734	VTGWGNLK	+2y7	437.74	775.41	63
286	F2	P00734	VTGWGNLK	+2y6	437.74	674.36	63
287	F2	P00734	VTGWGNLK	+2y5	437.74	617.34	63

288	F2	P00734	VTGWGNLK	+2y4	437.74	431.26	63
289	F2	P00734	ETWTANVGK	+2y7	503.25	775.41	67.8
290	F2	P00734	ETWTANVGK	+2y6	503.25	589.33	67.8
291	F2	P00734	ETWTANVGK	+2y5	503.25	488.28	67.8
292	F2	P00734	ETWTANVGK	+2y4	503.25	417.25	67.8
293	F2	P00734	ETWTANVGK	+2y3	503.25	303.20	67.8
294	RBP3	P10745	DVVVLTSSQTR	+2y9	602.83	990.56	75.1
295	RBP3	P10745	DVVVLTSSQTR	+2y7	602.83	792.42	75.1
296	RBP3	P10745	DVVVLTSSQTR	+2y6	602.83	679.34	75.1
297	RBP3	P10745	DVVVLTSSQTR	+2y5	602.83	578.29	75.1
298	RBP3	P10745	DVVVLTSSQTR	+2y4	602.83	491.26	75.1
299	RBP3	P10745	LNAGLQAASEDPR	+2y11	671.34	1114.55	80.1
300	RBP3	P10745	LNAGLQAASEDPR	+2y10	671.34	1043.51	80.1
301	RBP3	P10745	LNAGLQAASEDPR	+2y8	671.34	873.41	80.1
302	RBP3	P10745	LNAGLQAASEDPR	+2y7	671.34	745.35	80.1
303	RBP3	P10745	LNAGLQAASEDPR	+2y5	671.34	603.27	80.1
304	PTGDS	P41222	WFSAGLASNSSWLR	+2y12	791.39	1248.63	88.8
305	PTGDS	P41222	WFSAGLASNSSWLR	+2y10	791.39	1090.56	88.8
306	PTGDS	P41222	WFSAGLASNSSWLR	+2y8	791.39	920.46	88.8
307	PTGDS	P41222	WFSAGLASNSSWLR	+2y7	791.39	849.42	88.8
308	PTGDS	P41222	WFSAGLASNSSWLR	+2y6	791.39	762.39	88.8
309	PTGDS	P41222	SVVAPATDGGLNLTSTFLR	+2y12	960.02	1293.68	101.1
310	PTGDS	P41222	SVVAPATDGGLNLTSTFLR	+2y11	960.02	1178.65	101.1
311	PTGDS	P41222	SVVAPATDGGLNLTSTFLR	+2y7	960.02	837.48	101.1
312	PTGDS	P41222	SVVAPATDGGLNLTSTFLR	+2y6	960.02	724.40	101.1
313	PTGDS	P41222	SVVAPATDGGLNLTSTFLR	+2y5	960.02	623.35	101.1
314	PTGDS	P41222	GPGEDFR	+2y5	389.18	623.28	59.5
315	PTGDS	P41222	GPGEDFR	+2y4	389.18	566.26	59.5
316	PTGDS	P41222	GPGEDFR	+2y3	389.18	437.21	59.5
317	PTGDS	P41222	GPGEDFR	+2y2	389.18	322.19	59.5
318	PTGDS	P41222	AQGFTEDTIVFLPQTDK	+2y8	955.48	947.52	100.8
319	PTGDS	P41222	AQGFTEDTIVFLPQTDK	+2y7	955.48	848.45	100.8
320	PTGDS	P41222	AQGFTEDTIVFLPQTDK	+2y6	955.48	701.38	100.8
321	PTGDS	P41222	AQGFTEDTIVFLPQTDK	+2y5	955.48	588.30	100.8
322	GPX3	P22352	QEPGENSEILPTLK	+2y12	777.90	1297.70	87.8
323	GPX3	P22352	QEPGENSEILPTLK	+2y9	777.90	1014.58	87.8
324	GPX3	P22352	QEPGENSEILPTLK	+2y8	777.90	900.54	87.8
325	GPX3	P22352	QEPGENSEILPTLK	+2y5	777.90	571.38	87.8
326	GPX3	P22352	QEPGENSEILPTLK	+2y4	777.90	458.30	87.8
327	GPX3	P22352	YVRPGGGFVPNFQLFEK	+2y8	978.01	1022.53	102.4
328	GPX3	P22352	YVRPGGGFVPNFQLFEK	+2y7	978.01	925.48	102.4
329	GPX3	P22352	YVRPGGGFVPNFQLFEK	+2y6	978.01	811.43	102.4
330	GPX3	P22352	YVRPGGGFVPNFQLFEK	+2y4	978.01	536.31	102.4
331	GPX3	P22352	YVRPGGGFVPNFQLFEK	+2y3	978.01	423.22	102.4
332	ACTB	P60709	AVFPSIVGRPR	+2y8	599.86	881.53	74.8
333	ACTB	P60709	AVFPSIVGRPR	+2y7	599.86	784.48	74.8
334	ACTB	P60709	AVFPSIVGRPR	+2y6	599.86	697.45	74.8
335	ACTB	P60709	AVFPSIVGRPR	+2y5	599.86	584.36	74.8
336	ACTB	P60709	AVFPSIVGRPR	+2y4	599.86	485.29	74.8
337	ACTB	P60709	DSYVGDEAQSK	+2y8	599.76	833.40	74.8

338	ACTB	P60709	DSYVGDEAQSK	+2y7	599.76	734.33	74.8
339	ACTB	P60709	DSYVGDEAQSK	+2y6	599.76	677.31	74.8
340	ACTB	P60709	DSYVGDEAQSK	+2y5	599.76	562.28	74.8
341	ACTB	P60709	DSYVGDEAQSK	+2y4	599.76	433.24	74.8
342	ACTB	P60709	GYSFTTTAER	+2y8	566.77	912.44	72.4
343	ACTB	P60709	GYSFTTTAER	+2y7	566.77	825.41	72.4
344	ACTB	P60709	GYSFTTTAER	+2y6	566.77	678.34	72.4
345	ACTB	P60709	GYSFTTTAER	+2y5	566.77	577.29	72.4
346	ACTB	P60709	GYSFTTTAER	+2y4	566.77	476.25	72.4
347	ACTB	P60709	SYELPDGQVITIGNER	+2y12	895.95	1298.67	96.4
348	ACTB	P60709	SYELPDGQVITIGNER	+2y10	895.95	1086.59	96.4
349	ACTB	P60709	SYELPDGQVITIGNER	+2y7	895.95	802.44	96.4
350	ACTB	P60709	SYELPDGQVITIGNER	+2y6	895.95	689.36	96.4
351	ACTB	P60709	SYELPDGQVITIGNER	+2y4	895.95	475.23	96.4
352	ACTB	P60709	IIAPPER	+2y6	398.24	682.39	60.1
353	ACTB	P60709	IIAPPER	+2y5	398.24	569.30	60.1
354	ACTB	P60709	IIAPPER	+2y4	398.24	498.27	60.1
355	ACTB	P60709	IIAPPER	+2y3	398.24	401.21	60.1
356	ACTB	P60709	IIAPPER	+2y2	398.24	304.16	60.1
357	AFM	P43652	SDVGFLPPFPTLDPEEK	+2y11	944.47	1269.64	100
358	AFM	P43652	SDVGFLPPFPTLDPEEK	+2y8	944.47	928.46	100
359	AFM	P43652	SDVGFLPPFPTLDPEEK	+2y6	944.47	730.36	100
360	AFM	P43652	SDVGFLPPFPTLDPEEK	+2y5	944.47	617.28	100
361	AFM	P43652	SDVGFLPPFPTLDPEEK	+2y4	944.47	502.25	100
362	AFM	P43652	DADPDTFFAK	+2y8	563.76	940.44	72.2
363	AFM	P43652	DADPDTFFAK	+2y7	563.76	825.41	72.2
364	AFM	P43652	DADPDTFFAK	+2y6	563.76	728.36	72.2
365	AFM	P43652	DADPDTFFAK	+2y5	563.76	613.33	72.2
366	AFM	P43652	DADPDTFFAK	+2y3	563.76	365.22	72.2
367	AFM	P43652	FTFEYSR	+2y6	475.22	802.37	65.8
368	AFM	P43652	FTFEYSR	+2y5	475.22	701.33	65.8
369	AFM	P43652	FTFEYSR	+2y4	475.22	554.26	65.8
370	AFM	P43652	FTFEYSR	+2y3	475.22	425.21	65.8
371	AFM	P43652	IAPQLSTEELVSLGEK	+2y12	857.47	1304.69	93.6
372	AFM	P43652	IAPQLSTEELVSLGEK	+2y11	857.47	1191.61	93.6
373	AFM	P43652	IAPQLSTEELVSLGEK	+2y8	857.47	874.49	93.6
374	AFM	P43652	IAPQLSTEELVSLGEK	+2y5	857.47	533.29	93.6
375	AFM	P43652	IAPQLSTEELVSLGEK	+2y4	857.47	446.26	93.6
376	AFM	P43652	FLVNLVK	+2y6	416.77	685.46	61.5
377	AFM	P43652	FLVNLVK	+2y5	416.77	572.38	61.5
378	AFM	P43652	FLVNLVK	+2y4	416.77	473.31	61.5
379	AFM	P43652	FLVNLVK	+2y3	416.77	359.27	61.5
380	SERPINC1	P01008	ATEDEGSEQK	+2y8	547.24	921.38	71
381	SERPINC1	P01008	ATEDEGSEQK	+2y7	547.24	792.34	71
382	SERPINC1	P01008	ATEDEGSEQK	+2y6	547.24	677.31	71
383	SERPINC1	P01008	ATEDEGSEQK	+2y5	547.24	548.27	71
384	SERPINC1	P01008	ATEDEGSEQK	+2y4	547.24	491.25	71
385	SERPINC1	P01008	SLTFNETYQDISELVYGAK	+2y12	1089.53	1385.69	110.5
386	SERPINC1	P01008	SLTFNETYQDISELVYGAK	+2y11	1089.53	1222.63	110.5
387	SERPINC1	P01008	SLTFNETYQDISELVYGAK	+2y8	1089.53	866.46	110.5

388	SERPINC1	P01008	SLTFNETYQDISELVYGAK	+2y6	1089.53	650.39	110.5
389	SERPINC1	P01008	SLTFNETYQDISELVYGAK	+2y4	1089.53	438.23	110.5
390	SERPINC1	P01008	VAEGTQVLELPFK	+2y10	715.90	1131.64	83.3
391	SERPINC1	P01008	VAEGTQVLELPFK	+2y7	715.90	845.51	83.3
392	SERPINC1	P01008	VAEGTQVLELPFK	+2y6	715.90	746.44	83.3
393	SERPINC1	P01008	VAEGTQVLELPFK	+2y4	715.90	504.32	83.3
394	SERPINC1	P01008	VAEGTQVLELPFK	+2y3	715.90	391.23	83.3
395	SERPINC1	P01008	IEDGFSLK	+2y7	454.74	795.39	64.3
396	SERPINC1	P01008	IEDGFSLK	+2y6	454.74	666.35	64.3
397	SERPINC1	P01008	IEDGFSLK	+2y5	454.74	551.32	64.3
398	SERPINC1	P01008	IEDGFSLK	+2y4	454.74	494.30	64.3
399	SERPINC1	P01008	IEDGFSLK	+2y3	454.74	347.23	64.3
400	SERPINC1	P01008	LPGIVAEGR	+2y8	456.27	798.45	64.4
401	SERPINC1	P01008	LPGIVAEGR	+2y7	456.27	701.39	64.4
402	SERPINC1	P01008	LPGIVAEGR	+2y6	456.27	644.37	64.4
403	SERPINC1	P01008	LPGIVAEGR	+2y5	456.27	531.29	64.4
404	SERPINC1	P01008	LPGIVAEGR	+2y4	456.27	432.22	64.4
405	APOA2	P02652	SPELQAEAK	+2y7	486.75	788.41	66.6
406	APOA2	P02652	SPELQAEAK	+2y6	486.75	659.37	66.6
407	APOA2	P02652	SPELQAEAK	+2y5	486.75	546.29	66.6
408	APOA2	P02652	SPELQAEAK	+2y4	486.75	418.23	66.6
409	APOA2	P02652	SPELQAEAK	+2y3	486.75	347.19	66.6
410	APOA2	P02652	EQLTPLIK	+2y6	471.29	684.47	65.5
411	APOA2	P02652	EQLTPLIK	+2y5	471.29	571.38	65.5
412	APOA2	P02652	EQLTPLIK	+2y4	471.29	470.33	65.5
413	APOA2	P02652	EQLTPLIK	+2y3	471.29	373.28	65.5
414	APOB	P04114	LEVLNFDFQANAQLSNPK	+2y12	1024.53	1332.65	105.8
415	APOB	P04114	LEVLNFDFQANAQLSNPK	+2y11	1024.53	1217.63	105.8
416	APOB	P04114	LEVLNFDFQANAQLSNPK	+2y10	1024.53	1070.56	105.8
417	APOB	P04114	LEVLNFDFQANAQLSNPK	+2y9	1024.53	942.50	105.8
418	APOB	P04114	LEVLNFDFQANAQLSNPK	+2y8	1024.53	871.46	105.8
419	APOB	P04114	NNALDFVTK	+2y7	511.27	793.45	68.4
420	APOB	P04114	NNALDFVTK	+2y6	511.27	722.41	68.4
421	APOB	P04114	NNALDFVTK	+2y5	511.27	609.32	68.4
422	APOB	P04114	NNALDFVTK	+2y4	511.27	494.30	68.4
423	APOB	P04114	NNALDFVTK	+2y3	511.27	347.23	68.4
424	APOB	P04114	SVSDGIAALDLNAVANK	+2y11	829.44	1099.61	91.6
425	APOB	P04114	SVSDGIAALDLNAVANK	+2y10	829.44	1028.57	91.6
426	APOB	P04114	SVSDGIAALDLNAVANK	+2y9	829.44	957.54	91.6
427	APOB	P04114	SVSDGIAALDLNAVANK	+2y8	829.44	844.45	91.6
428	APOB	P04114	SVSDGIAALDLNAVANK	+2y3	829.44	332.19	91.6
429	APOB	P04114	WNFYYSPQSSPDK	+2y9	809.86	1008.46	90.2
430	APOB	P04114	WNFYYSPQSSPDK	+2y8	809.86	845.40	90.2
431	APOB	P04114	WNFYYSPQSSPDK	+2y7	809.86	758.37	90.2
432	APOB	P04114	WNFYYSPQSSPDK	+2y5	809.86	533.26	90.2
433	APOB	P04114	WNFYYSPQSSPDK	+2y3	809.86	359.19	90.2
434	APOB	P04114	NLQDLLQFIFQLIEDNIK	+2y11	1102.60	1379.76	111.5
435	APOB	P04114	NLQDLLQFIFQLIEDNIK	+2y10	1102.60	1232.69	111.5
436	APOB	P04114	NLQDLLQFIFQLIEDNIK	+2y9	1102.60	1119.60	111.5
437	APOB	P04114	NLQDLLQFIFQLIEDNIK	+2y8	1102.60	972.54	111.5

438	APOB	P04114	NLQDLLQFIFQLIEDNIK	+2y5	1102.60	618.31	111.5
439	APOD	P05090	IPTTFENGR	+2y8	517.77	921.44	68.9
440	APOD	P05090	IPTTFENGR	+2y7	517.77	824.39	68.9
441	APOD	P05090	IPTTFENGR	+2y6	517.77	723.34	68.9
442	APOD	P05090	IPTTFENGR	+2y5	517.77	622.29	68.9
443	APOD	P05090	IPTTFENGR	+2y4	517.77	475.23	68.9
444	APOD	P05090	VLNQELR	+2y6	436.25	772.43	62.9
445	APOD	P05090	VLNQELR	+2y5	436.25	659.35	62.9
446	APOD	P05090	VLNQELR	+2y4	436.25	545.30	62.9
447	APOD	P05090	VLNQELR	+2y3	436.25	417.25	62.9
448	APOD	P05090	NPNLPPETVDSLK	+2y10	712.38	1098.60	83
449	APOD	P05090	NPNLPPETVDSLK	+2y9	712.38	985.52	83
450	APOD	P05090	NPNLPPETVDSLK	+2y8	712.38	888.47	83
451	APOD	P05090	NPNLPPETVDSLK	+2y5	712.38	561.32	83
452	APOD	P05090	NILTSNNIDVK	+2y9	615.84	1003.54	76
453	APOD	P05090	NILTSNNIDVK	+2y8	615.84	890.46	76
454	APOD	P05090	NILTSNNIDVK	+2y7	615.84	789.41	76
455	APOD	P05090	NILTSNNIDVK	+2y6	615.84	702.38	76
456	APOD	P05090	NILTSNNIDVK	+2y3	615.84	361.21	76
457	C9	P02748	SIEVFGQFNGK	+2y9	613.31	1025.51	75.8
458	C9	P02748	SIEVFGQFNGK	+2y8	613.31	896.46	75.8
459	C9	P02748	SIEVFGQFNGK	+2y7	613.31	797.39	75.8
460	C9	P02748	SIEVFGQFNGK	+2y6	613.31	650.33	75.8
461	C9	P02748	SIEVFGQFNGK	+2y4	613.31	465.25	75.8
462	C9	P02748	RPWNVASLIYETK	+2y8	788.93	924.50	88.6
463	C9	P02748	RPWNVASLIYETK	+2y7	788.93	853.47	88.6
464	C9	P02748	RPWNVASLIYETK	+2y6	788.93	766.43	88.6
465	C9	P02748	RPWNVASLIYETK	+2y5	788.93	653.35	88.6
466	C9	P02748	RPWNVASLIYETK	+2y4	788.93	540.27	88.6
467	C9	P02748	TSNFNAAISLK	+2y9	583.31	977.54	73.6
468	C9	P02748	TSNFNAAISLK	+2y8	583.31	863.50	73.6
469	C9	P02748	TSNFNAAISLK	+2y7	583.31	716.43	73.6
470	C9	P02748	TSNFNAAISLK	+2y6	583.31	602.39	73.6
471	C9	P02748	TSNFNAAISLK	+2y3	583.31	347.23	73.6
472	C9	P02748	AVNITSENLIDDVVSLIR	+2y12	986.04	1385.76	103
473	C9	P02748	AVNITSENLIDDVVSLIR	+2y9	986.04	1029.59	103
474	C9	P02748	AVNITSENLIDDVVSLIR	+2y8	986.04	916.51	103
475	C9	P02748	AVNITSENLIDDVVSLIR	+2y5	986.04	587.39	103
476	C9	P02748	AVNITSENLIDDVVSLIR	+2y4	986.04	488.32	103
477	C9	P02748	AIEDYINEFSVR	+2y10	728.36	1271.59	84.2
478	C9	P02748	AIEDYINEFSVR	+2y9	728.36	1142.55	84.2
479	C9	P02748	AIEDYINEFSVR	+2y7	728.36	864.46	84.2
480	C9	P02748	AIEDYINEFSVR	+2y6	728.36	751.37	84.2
481	C9	P02748	AIEDYINEFSVR	+2y4	728.36	508.29	84.2
482	CFH	P08603	SPDVINGSPISQK	+2y9	671.35	943.52	80.1
483	CFH	P08603	SPDVINGSPISQK	+2y8	671.35	830.44	80.1
484	CFH	P08603	SPDVINGSPISQK	+2y7	671.35	716.39	80.1
485	CFH	P08603	SPDVINGSPISQK	+2y5	671.35	572.34	80.1
486	CFH	P08603	SPDVINGSPISQK	+2y3	671.35	362.20	80.1
487	CFH	P08603	RPYFPVAVGK	+2y6	567.33	570.36	72.5

488	CFH	P08603	RPYFPVAVGK	+2y3	567.33	303.20	72.5
489	CFH	P08603	SSIDIENGFISESQYTYALK	+2y11	1133.05	1302.66	113.7
490	CFH	P08603	SSIDIENGFISESQYTYALK	+2y10	1133.05	1189.57	113.7
491	CFH	P08603	SSIDIENGFISESQYTYALK	+2y9	1133.05	1102.54	113.7
492	CFH	P08603	SSIDIENGFISESQYTYALK	+2y8	1133.05	973.50	113.7
493	CFH	P08603	SSIDIENGFISESQYTYALK	+2y6	1133.05	758.41	113.7
494	CFH	P08603	NDFTWFK	+2y6	479.23	843.40	66.1
495	CFH	P08603	NDFTWFK	+2y5	479.23	728.38	66.1
496	CFH	P08603	NDFTWFK	+2y4	479.23	581.31	66.1
497	CFH	P08603	NDFTWFK	+2y3	479.23	480.26	66.1
498	CFH	P08603	NGQWSEPPK	+2y7	521.75	871.43	69.2
499	CFH	P08603	NGQWSEPPK	+2y6	521.75	743.37	69.2
500	CFH	P08603	NGQWSEPPK	+2y5	521.75	557.29	69.2
501	CFH	P08603	NGQWSEPPK	+2y4	521.75	470.26	69.2
502	CFH	P08603	NGQWSEPPK	+2y3	521.75	341.22	69.2
503	FGA	P02671	GLIDEVNQDFTNR	+2y10	760.87	1237.54	86.6
504	FGA	P02671	GLIDEVNQDFTNR	+2y8	760.87	993.47	86.6
505	FGA	P02671	GLIDEVNQDFTNR	+2y7	760.87	894.41	86.6
506	FGA	P02671	GLIDEVNQDFTNR	+2y5	760.87	652.30	86.6
507	FGA	P02671	GLIDEVNQDFTNR	+2y4	760.87	537.28	86.6
508	FGA	P02671	NSLFEYQK	+2y6	514.76	827.43	68.6
509	FGA	P02671	NSLFEYQK	+2y5	514.76	714.35	68.6
510	FGA	P02671	NSLFEYQK	+2y4	514.76	567.28	68.6
511	FGA	P02671	NSLFEYQK	+2y3	514.76	438.23	68.6
512	FGA	P02671	GGSTSYGTGSETESPR	+2y11	786.84	1183.52	88.5
513	FGA	P02671	GGSTSYGTGSETESPR	+2y10	786.84	1020.46	88.5
514	FGA	P02671	GGSTSYGTGSETESPR	+2y8	786.84	862.39	88.5
515	FGA	P02671	GGSTSYGTGSETESPR	+2y5	786.84	589.29	88.5
516	FGA	P02671	GGSTSYGTGSETESPR	+2y3	786.84	359.20	88.5
517	FGA	P02671	QFTSSTSYNR	+2y8	595.78	915.42	74.5
518	FGA	P02671	QFTSSTSYNR	+2y7	595.78	814.37	74.5
519	FGA	P02671	QFTSSTSYNR	+2y6	595.78	727.34	74.5
520	FGA	P02671	QFTSSTSYNR	+2y5	595.78	640.30	74.5
521	FGA	P02671	QFTSSTSYNR	+2y4	595.78	539.26	74.5
522	FGA	P02671	NNSPYEIENGVVWVSFR	+2y10	1005.49	1192.61	104.4
523	FGA	P02671	NNSPYEIENGVVWVSFR	+2y6	1005.49	793.44	104.4
524	FGA	P02671	NNSPYEIENGVVWVSFR	+2y5	1005.49	694.37	104.4
525	FGA	P02671	NNSPYEIENGVVWVSFR	+2y4	1005.49	508.29	104.4
526	FGA	P02671	NNSPYEIENGVVWVSFR	+2y3	1005.49	409.22	104.4
527	GSN	P06396	EVQGFESATFLGYFK	+2y12	861.92	1366.67	94
528	GSN	P06396	EVQGFESATFLGYFK	+2y10	861.92	1162.58	94
529	GSN	P06396	EVQGFESATFLGYFK	+2y9	861.92	1033.54	94
530	GSN	P06396	EVQGFESATFLGYFK	+2y7	861.92	875.47	94
531	GSN	P06396	EVQGFESATFLGYFK	+2y4	861.92	514.27	94
532	GSN	P06396	GGVASGFK	+2y6	361.70	608.34	57.5
533	GSN	P06396	GGVASGFK	+2y5	361.70	509.27	57.5
534	GSN	P06396	GGVASGFK	+2y4	361.70	438.23	57.5
535	GSN	P06396	GGVASGFK	+2y3	361.70	351.20	57.5
536	GSN	P06396	AGALNSNDAFVLK	+2y9	660.35	1007.52	79.3
537	GSN	P06396	AGALNSNDAFVLK	+2y8	660.35	893.47	79.3

538	GSN	P06396	AGALNSNDAFVLK	+2y7	660.35	806.44	79.3
539	GSN	P06396	AGALNSNDAFVLK	+2y5	660.35	577.37	79.3
540	GSN	P06396	AGALNSNDAFVLK	+2y3	660.35	359.27	79.3
541	GSN	P06396	TPSAAYLWVGTGASEAEK	+2y11	919.45	1134.54	98.1
542	GSN	P06396	TPSAAYLWVGTGASEAEK	+2y10	919.45	948.46	98.1
543	GSN	P06396	TPSAAYLWVGTGASEAEK	+2y9	919.45	849.39	98.1
544	GSN	P06396	TPSAAYLWVGTGASEAEK	+2y7	919.45	691.33	98.1
545	GSN	P06396	TPSAAYLWVGTGASEAEK	+2y5	919.45	563.27	98.1
546	GSN	P06396	TPITVVK	+2y6	379.24	656.43	58.8
547	GSN	P06396	TPITVVK	+2y5	379.24	559.38	58.8
548	GSN	P06396	TPITVVK	+2y4	379.24	446.30	58.8
549	GSN	P06396	TPITVVK	+2y3	379.24	345.25	58.8
550	CFB	P00751	STGSWSTLK	+2y7	483.75	778.41	66.4
551	CFB	P00751	STGSWSTLK	+2y6	483.75	721.39	66.4
552	CFB	P00751	STGSWSTLK	+2y5	483.75	634.36	66.4
553	CFB	P00751	STGSWSTLK	+2y4	483.75	448.28	66.4
554	CFB	P00751	STGSWSTLK	+2y3	483.75	361.24	66.4
555	CFB	P00751	YGLVTYATYPK	+2y8	638.33	942.49	77.7
556	CFB	P00751	YGLVTYATYPK	+2y7	638.33	843.42	77.7
557	CFB	P00751	YGLVTYATYPK	+2y6	638.33	742.38	77.7
558	CFB	P00751	YGLVTYATYPK	+2y5	638.33	579.31	77.7
559	CFB	P00751	YGLVTYATYPK	+2y3	638.33	407.23	77.7
560	CFB	P00751	VSEADSSNADWVTK	+2y10	754.85	1122.51	86.1
561	CFB	P00751	VSEADSSNADWVTK	+2y9	754.85	1007.48	86.1
562	CFB	P00751	VSEADSSNADWVTK	+2y8	754.85	920.45	86.1
563	CFB	P00751	VSEADSSNADWVTK	+2y7	754.85	833.42	86.1
564	CFB	P00751	VSEADSSNADWVTK	+2y4	754.85	533.31	86.1
565	CFB	P00751	ISVIRPSK	+2y7	450.29	786.48	63.9
566	CFB	P00751	ISVIRPSK	+2y6	450.29	699.45	63.9
567	CFB	P00751	ISVIRPSK	+2y5	450.29	600.38	63.9
568	CFB	P00751	ISVIRPSK	+2y4	450.29	487.30	63.9
569	CFB	P00751	ISVIRPSK	+2y3	450.29	331.20	63.9
570	CFB	P00751	DAQYAPGYDK	+2y7	564.25	813.38	72.3
571	CFB	P00751	DAQYAPGYDK	+2y6	564.25	650.31	72.3
572	CFB	P00751	DAQYAPGYDK	+2y5	564.25	579.28	72.3
573	CFB	P00751	DAQYAPGYDK	+2y4	564.25	482.22	72.3
574	CFB	P00751	DAQYAPGYDK	+2y3	564.25	425.20	72.3
575	AHSG	P02765	FSVVYAK	+2y6	407.23	666.38	60.8
576	AHSG	P02765	FSVVYAK	+2y5	407.23	579.35	60.8
577	AHSG	P02765	FSVVYAK	+2y4	407.23	480.28	60.8
578	AHSG	P02765	FSVVYAK	+2y3	407.23	381.21	60.8
579	GC	D6RF35	EDFTSLSLVLYSR	+2y7	765.40	837.48	86.9
580	GC	D6RF35	EDFTSLSLVLYSR	+2y6	765.40	750.45	86.9
581	GC	D6RF35	EDFTSLSLVLYSR	+2y5	765.40	637.37	86.9
582	GC	D6RF35	EDFTSLSLVLYSR	+2y4	765.40	538.30	86.9
583	GC	D6RF35	EDFTSLSLVLYSR	+2y3	765.40	425.21	86.9
584	GC	D6RF35	FPSGTFEQVSQLVK	+2y9	783.91	1077.59	88.3
585	GC	D6RF35	FPSGTFEQVSQLVK	+2y8	783.91	930.53	88.3
586	GC	D6RF35	FPSGTFEQVSQLVK	+2y7	783.91	801.48	88.3
587	GC	D6RF35	FPSGTFEQVSQLVK	+2y6	783.91	673.42	88.3

588	GC	D6RF35	FPSGTFEQVSQLVK	+2y5	783.91	574.36	88.3
589	GC	D6RF35	VLEPTLK	+2y6	400.25	700.42	60.3
590	GC	D6RF35	VLEPTLK	+2y5	400.25	587.34	60.3
591	GC	D6RF35	VLEPTLK	+2y4	400.25	458.30	60.3
592	GC	D6RF35	VLEPTLK	+2y3	400.25	361.24	60.3
593	GC	D6RF35	ELSSFIDK	+2y6	469.75	696.36	65.4
594	GC	D6RF35	ELSSFIDK	+2y5	469.75	609.32	65.4
595	GC	D6RF35	ELSSFIDK	+2y4	469.75	522.29	65.4
596	GC	D6RF35	ELSSFIDK	+2y3	469.75	375.22	65.4
597	GC	D6RF35	LPDATPTELAK	+2y10	578.32	1042.54	73.3
598	GC	D6RF35	LPDATPTELAK	+2y9	578.32	945.49	73.3
599	GC	D6RF35	LPDATPTELAK	+2y8	578.32	830.46	73.3
600	GC	D6RF35	LPDATPTELAK	+2y7	578.32	759.42	73.3
601	GC	D6RF35	LPDATPTELAK	+2y6	578.32	658.38	73.3
602	ITIH2	P19823	VQSTITSR	+2y7	446.25	792.42	63.6
603	ITIH2	P19823	VQSTITSR	+2y6	446.25	664.36	63.6
604	ITIH2	P19823	VQSTITSR	+2y5	446.25	577.33	63.6
605	ITIH2	P19823	VQSTITSR	+2y4	446.25	476.28	63.6
606	ITIH2	P19823	VQSTITSR	+2y3	446.25	363.20	63.6
607	ITIH2	P19823	ETAVDGELVVLYDVK	+2y11	825.44	1249.67	91.3
608	ITIH2	P19823	ETAVDGELVVLYDVK	+2y7	825.44	835.49	91.3
609	ITIH2	P19823	ETAVDGELVVLYDVK	+2y6	825.44	736.42	91.3
610	ITIH2	P19823	ETAVDGELVVLYDVK	+2y5	825.44	637.36	91.3
611	ITIH2	P19823	ETAVDGELVVLYDVK	+2y4	825.44	524.27	91.3
612	ITIH2	P19823	IQPSGGTNINEALLR	+2y13	791.93	1341.71	88.8
613	ITIH2	P19823	IQPSGGTNINEALLR	+2y11	791.93	1157.63	88.8
614	ITIH2	P19823	IQPSGGTNINEALLR	+2y8	791.93	942.54	88.8
615	ITIH2	P19823	IQPSGGTNINEALLR	+2y6	791.93	715.41	88.8
616	ITIH2	P19823	IQPSGGTNINEALLR	+2y4	791.93	472.32	88.8
617	ITIH2	P19823	IYGNQDTSSQLK	+2y10	677.34	1077.52	80.5
618	ITIH2	P19823	IYGNQDTSSQLK	+2y8	677.34	906.45	80.5
619	ITIH2	P19823	IYGNQDTSSQLK	+2y7	677.34	778.39	80.5
620	ITIH2	P19823	IYGNQDTSSQLK	+2y6	677.34	663.37	80.5
621	ITIH2	P19823	IYGNQDTSSQLK	+2y5	677.34	562.32	80.5
622	ITIH2	P19823	LWAYLTINQLLAER	+2y9	852.48	1057.60	93.3
623	ITIH2	P19823	LWAYLTINQLLAER	+2y8	852.48	956.55	93.3
624	ITIH2	P19823	LWAYLTINQLLAER	+2y7	852.48	843.47	93.3
625	ITIH2	P19823	LWAYLTINQLLAER	+2y5	852.48	601.37	93.3
626	ITIH2	P19823	LWAYLTINQLLAER	+2y3	852.48	375.20	93.3
627	ITIH1	P19827	QAVDTAVDGVFIR	+2y10	695.87	1092.57	81.8
628	ITIH1	P19827	QAVDTAVDGVFIR	+2y8	695.87	876.49	81.8
629	ITIH1	P19827	QAVDTAVDGVFIR	+2y7	695.87	805.46	81.8
630	ITIH1	P19827	QAVDTAVDGVFIR	+2y6	695.87	706.39	81.8
631	ITIH1	P19827	QAVDTAVDGVFIR	+2y5	695.87	591.36	81.8
632	ITIH1	P19827	LDAQASFLPK	+2y9	545.30	976.51	70.9
633	ITIH1	P19827	LDAQASFLPK	+2y8	545.30	861.48	70.9
634	ITIH1	P19827	LDAQASFLPK	+2y7	545.30	790.45	70.9
635	ITIH1	P19827	LDAQASFLPK	+2y6	545.30	662.39	70.9
636	ITIH1	P19827	LDAQASFLPK	+2y5	545.30	591.35	70.9
637	ITIH1	P19827	GSLVQASEANLQAAQDFVR	+2y10	1002.51	1161.60	104.2

638	ITIH1	P19827	GSLVQASEANLQAAQDFVR	+2y8	1002.51	934.47	104.2
639	ITIH1	P19827	GSLVQASEANLQAAQDFVR	+2y7	1002.51	806.42	104.2
640	ITIH1	P19827	GSLVQASEANLQAAQDFVR	+2y6	1002.51	735.38	104.2
641	ITIH1	P19827	GSLVQASEANLQAAQDFVR	+2y3	1002.51	421.26	104.2
642	ITIH1	P19827	QYYEGSEIVVAGR	+2y10	735.86	1016.54	84.8
643	ITIH1	P19827	QYYEGSEIVVAGR	+2y9	735.86	887.49	84.8
644	ITIH1	P19827	QYYEGSEIVVAGR	+2y5	735.86	501.31	84.8
645	ITIH1	P19827	QYYEGSEIVVAGR	+2y4	735.86	402.25	84.8
646	ITIH1	P19827	QYYEGSEIVVAGR	+2y3	735.86	303.18	84.8
647	ITIH1	P19827	LWAYLTIQELLAK	+2y11	781.45	1262.74	88.1
648	ITIH1	P19827	LWAYLTIQELLAK	+2y9	781.45	1028.64	88.1
649	ITIH1	P19827	LWAYLTIQELLAK	+2y8	781.45	915.55	88.1
650	ITIH1	P19827	LWAYLTIQELLAK	+2y7	781.45	814.50	88.1
651	ITIH1	P19827	LWAYLTIQELLAK	+2y3	781.45	331.23	88.1
652	PLG	P00747	DVVLFEK	+2y5	425.24	635.38	62.1
653	PLG	P00747	DVVLFEK	+2y4	425.24	536.31	62.1
654	PLG	P00747	DVVLFEK	+2y3	425.24	423.22	62.1
655	PLG	P00747	LFLEPTR	+2y6	438.25	762.41	63.1
656	PLG	P00747	LFLEPTR	+2y5	438.25	615.35	63.1
657	PLG	P00747	LFLEPTR	+2y4	438.25	502.26	63.1
658	PLG	P00747	LFLEPTR	+2y3	438.25	373.22	63.1
659	PLG	P00747	LSSPAVITDK	+2y9	515.79	917.49	68.7
660	PLG	P00747	LSSPAVITDK	+2y8	515.79	830.46	68.7
661	PLG	P00747	LSSPAVITDK	+2y7	515.79	743.43	68.7
662	PLG	P00747	LSSPAVITDK	+2y5	515.79	575.34	68.7
663	PLG	P00747	LSSPAVITDK	+2y4	515.79	476.27	68.7
664	PLG	P00747	EAQLPVIENK	+2y7	570.82	812.49	72.7
665	PLG	P00747	EAQLPVIENK	+2y6	570.82	699.40	72.7
666	PLG	P00747	EAQLPVIENK	+2y5	570.82	602.35	72.7
667	PLG	P00747	EAQLPVIENK	+2y4	570.82	503.28	72.7
668	PLG	P00747	EAQLPVIENK	+2y3	570.82	390.20	72.7
669	PLG	P00747	YEFLNGR	+2y6	449.72	735.38	63.9
670	PLG	P00747	YEFLNGR	+2y5	449.72	606.34	63.9
671	PLG	P00747	YEFLNGR	+2y4	449.72	459.27	63.9
672	PLG	P00747	YEFLNGR	+2y3	449.72	346.18	63.9
673	RBP4	Q5VY30	YWGVASFLQK	+2y8	599.82	849.48	74.8
674	RBP4	Q5VY30	YWGVASFLQK	+2y7	599.82	792.46	74.8
675	RBP4	Q5VY30	YWGVASFLQK	+2y6	599.82	693.39	74.8
676	RBP4	Q5VY30	YWGVASFLQK	+2y5	599.82	622.36	74.8
677	RBP4	Q5VY30	YWGVASFLQK	+2y3	599.82	388.26	74.8
678	RBP4	Q5VY30	DPNGLPPEAQK	+2y9	583.30	953.51	73.6
679	RBP4	Q5VY30	DPNGLPPEAQK	+2y8	583.30	839.46	73.6
680	RBP4	Q5VY30	DPNGLPPEAQK	+2y7	583.30	782.44	73.6
681	RBP4	Q5VY30	DPNGLPPEAQK	+2y6	583.30	669.36	73.6
682	RBP4	Q5VY30	DPNGLPPEAQK	+2y5	583.30	572.30	73.6
683	KNG1	P01042	YNSQNQSNNQFVLYR	+2y7	937.94	939.50	99.5
684	KNG1	P01042	YNSQNQSNNQFVLYR	+2y5	937.94	697.40	99.5
685	KNG1	P01042	YNSQNQSNNQFVLYR	+2y4	937.94	550.33	99.5
686	KNG1	P01042	YNSQNQSNNQFVLYR	+2y3	937.94	451.27	99.5
687	KNG1	P01042	YNSQNQSNNQFVLYR	+2y2	937.94	338.18	99.5

688	KNG1	P01042	QVVAGLNFR	+2y7	502.29	776.44	67.7
689	KNG1	P01042	QVVAGLNFR	+2y6	502.29	677.37	67.7
690	KNG1	P01042	QVVAGLNFR	+2y5	502.29	606.34	67.7
691	KNG1	P01042	QVVAGLNFR	+2y4	502.29	549.31	67.7
692	KNG1	P01042	QVVAGLNFR	+2y3	502.29	436.23	67.7
693	KNG1	P01042	DFVQPPTK	+2y6	466.25	669.39	65.1
694	KNG1	P01042	DFVQPPTK	+2y5	466.25	570.32	65.1
695	KNG1	P01042	DFVQPPTK	+2y4	466.25	442.27	65.1
696	KNG1	P01042	DFVQPPTK	+2y3	466.25	345.21	65.1
697	KNG1	P01042	RPPGFSPFR	+2y8	530.79	904.47	69.8
698	KNG1	P01042	RPPGFSPFR	+2y7	530.79	807.41	69.8
699	KNG1	P01042	RPPGFSPFR	+2y6	530.79	710.36	69.8
700	KNG1	P01042	RPPGFSPFR	+2y5	530.79	653.34	69.8
701	KNG1	P01042	RPPGFSPFR	+2y3	530.79	419.24	69.8
702	KNG1	P01042	ESYYFDLTDGLS	+2y9	705.31	1030.47	82.5
703	KNG1	P01042	ESYYFDLTDGLS	+2y8	705.31	867.41	82.5
704	KNG1	P01042	ESYYFDLTDGLS	+2y7	705.31	720.34	82.5
705	KNG1	P01042	ESYYFDLTDGLS	+2y6	705.31	605.31	82.5
706	KNG1	P01042	ESYYFDLTDGLS	+2y5	705.31	492.23	82.5
707	APP	E9PG40	TTSIATTTTTTTESVEEVVR	+2y11	1064.04	1249.63	108.7
708	APP	E9PG40	TTSIATTTTTTTESVEEVVR	+2y9	1064.04	1047.53	108.7
709	APP	E9PG40	TTSIATTTTTTTESVEEVVR	+2y8	1064.04	946.48	108.7
710	APP	E9PG40	TTSIATTTTTTTESVEEVVR	+2y7	1064.04	817.44	108.7
711	APP	E9PG40	TTSIATTTTTTTESVEEVVR	+2y5	1064.04	631.34	108.7
712	APP	E9PG40	WYFDVTEGK	+2y7	572.77	795.39	72.9
713	APP	E9PG40	WYFDVTEGK	+2y6	572.77	648.32	72.9
714	APP	E9PG40	WYFDVTEGK	+2y5	572.77	533.29	72.9
715	APP	E9PG40	WYFDVTEGK	+2y4	572.77	434.22	72.9
716	APP	E9PG40	WYFDVTEGK	+2y3	572.77	333.18	72.9
717	APP	E9PG40	LPTTAASTPDAVDK	+2y12	693.86	1176.57	81.7
718	APP	E9PG40	LPTTAASTPDAVDK	+2y10	693.86	974.48	81.7
719	APP	E9PG40	LPTTAASTPDAVDK	+2y8	693.86	832.40	81.7
720	APP	E9PG40	LPTTAASTPDAVDK	+2y7	693.86	745.37	81.7
721	APP	E9PG40	LPTTAASTPDAVDK	+2y6	693.86	644.32	81.7
722	APP	E9PG40	VESLEQEAANER	+2y8	687.83	946.42	81.3
723	APP	E9PG40	VESLEQEAANER	+2y7	687.83	817.38	81.3
724	APP	E9PG40	VESLEQEAANER	+2y5	687.83	560.28	81.3
725	APP	E9PG40	VESLEQEAANER	+2y4	687.83	489.24	81.3
726	APP	E9PG40	VESLEQEAANER	+2y3	687.83	418.20	81.3
727	APP	E9PG40	LVFFAEDVGSNK	+2y10	663.34	1113.52	79.5
728	APP	E9PG40	LVFFAEDVGSNK	+2y9	663.34	966.45	79.5
729	APP	E9PG40	LVFFAEDVGSNK	+2y8	663.34	819.38	79.5
730	APP	E9PG40	LVFFAEDVGSNK	+2y7	663.34	748.35	79.5
731	APP	E9PG40	LVFFAEDVGSNK	+2y4	663.34	405.21	79.5
732	APLP2	Q06481	WEPDPTGTK	+2y8	515.75	844.40	68.7
733	APLP2	Q06481	WEPDPTGTK	+2y7	515.75	715.36	68.7
734	APLP2	Q06481	WEPDPTGTK	+2y6	515.75	618.31	68.7
735	APLP2	Q06481	WEPDPTGTK	+2y5	515.75	503.28	68.7
736	APLP2	Q06481	WEPDPTGTK	+2y4	515.75	406.23	68.7
737	APLP2	Q06481	EWEEAELQAK	+2y7	616.79	788.41	76.1

738	APLP2	Q06481	EWEEAELQAK	+2y6	616.79	659.37	76.1
739	APLP2	Q06481	EWEEAELQAK	+2y5	616.79	588.34	76.1
740	APLP2	Q06481	EWEEAELQAK	+2y4	616.79	459.29	76.1
741	APLP2	Q06481	EWEEAELQAK	+2y3	616.79	346.21	76.1
742	APLP2	Q06481	VPYVAQEIQEEIDELLQEQR	+2y10	1215.11	1272.64	119.7
743	APLP2	Q06481	VPYVAQEIQEEIDELLQEQR	+2y8	1215.11	1030.52	119.7
744	APLP2	Q06481	VPYVAQEIQEEIDELLQEQR	+2y7	1215.11	915.49	119.7
745	APLP2	Q06481	VPYVAQEIQEEIDELLQEQR	+2y6	1215.11	786.45	119.7
746	APLP2	Q06481	VPYVAQEIQEEIDELLQEQR	+2y5	1215.11	673.36	119.7
747	APLP2	Q06481	GSGVGEQDGGLIGAEEK	+2y13	801.88	1302.62	89.6
748	APLP2	Q06481	GSGVGEQDGGLIGAEEK	+2y10	801.88	988.49	89.6
749	APLP2	Q06481	GSGVGEQDGGLIGAEEK	+2y9	801.88	873.47	89.6
750	APLP2	Q06481	GSGVGEQDGGLIGAEEK	+2y6	801.88	646.34	89.6
751	APLP2	Q06481	GSGVGEQDGGLIGAEEK	+2y5	801.88	533.26	89.6
752	APLP2	Q06481	VGGLEEER	+2y7	444.72	789.37	63.5
753	APLP2	Q06481	VGGLEEER	+2y6	444.72	732.35	63.5
754	APLP2	Q06481	VGGLEEER	+2y4	444.72	562.25	63.5
755	APLP2	Q06481	VGGLEEER	+2y3	444.72	433.20	63.5
756	APLP2	Q06481	VGGLEEER	+2y2	444.72	304.16	63.5
757	CDH2	P19022	VQYESSEPADFK	+2y10	700.32	1172.51	82.2
758	CDH2	P19022	VQYESSEPADFK	+2y9	700.32	1009.45	82.2
759	CDH2	P19022	VQYESSEPADFK	+2y8	700.32	880.40	82.2
760	CDH2	P19022	VQYESSEPADFK	+2y7	700.32	793.37	82.2
761	CDH2	P19022	VQYESSEPADFK	+2y5	700.32	577.30	82.2
762	CDH2	P19022	ESAEVEEIVFPR	+2y8	702.85	988.55	82.4
763	CDH2	P19022	ESAEVEEIVFPR	+2y7	702.85	889.48	82.4
764	CDH2	P19022	ESAEVEEIVFPR	+2y5	702.85	631.39	82.4
765	CDH2	P19022	ESAEVEEIVFPR	+2y4	702.85	518.31	82.4
766	CDH2	P19022	ESAEVEEIVFPR	+2y3	702.85	419.24	82.4
767	CDH2	P19022	LSDPANWLK	+2y8	522.28	930.47	69.2
768	CDH2	P19022	LSDPANWLK	+2y7	522.28	843.44	69.2
769	CDH2	P19022	LSDPANWLK	+2y6	522.28	728.41	69.2
770	CDH2	P19022	LSDPANWLK	+2y5	522.28	631.36	69.2
771	CDH2	P19022	LSDPANWLK	+2y3	522.28	446.28	69.2
772	CDH2	P19022	LNGDFAQLNLK	+2y9	616.84	1005.54	76.1
773	CDH2	P19022	LNGDFAQLNLK	+2y7	616.84	833.49	76.1
774	CDH2	P19022	LNGDFAQLNLK	+2y6	616.84	686.42	76.1
775	CDH2	P19022	LNGDFAQLNLK	+2y4	616.84	487.32	76.1
776	CDH2	P19022	LNGDFAQLNLK	+2y3	616.84	374.24	76.1
777	CDH2	P19022	QLLIDPEDDVR	+2y9	656.84	1071.53	79
778	CDH2	P19022	QLLIDPEDDVR	+2y8	656.84	958.45	79
779	CDH2	P19022	QLLIDPEDDVR	+2y7	656.84	845.36	79
780	CDH2	P19022	QLLIDPEDDVR	+2y6	656.84	730.34	79
781	CDH2	P19022	QLLIDPEDDVR	+2y3	656.84	389.21	79
782	CPE	P16870	SNAQGIDLNR	+2y8	544.28	886.47	70.8
783	CPE	P16870	SNAQGIDLNR	+2y7	544.28	815.44	70.8
784	CPE	P16870	SNAQGIDLNR	+2y6	544.28	687.38	70.8
785	CPE	P16870	SNAQGIDLNR	+2y5	544.28	630.36	70.8
786	CPE	P16870	SNAQGIDLNR	+2y4	544.28	517.27	70.8
787	CPE	P16870	TYWEDNK	+2y5	478.21	691.30	66

788	CPE	P16870	TYWEDNK	+2y4	478.21	505.23	66
789	CPE	P16870	TYWEDNK	+2y3	478.21	376.18	66
790	CPE	P16870	LLIPGNYK	+2y7	459.28	804.46	64.6
791	CPE	P16870	LLIPGNYK	+2y6	459.28	691.38	64.6
792	CPE	P16870	LLIPGNYK	+2y5	459.28	578.29	64.6
793	CPE	P16870	LLIPGNYK	+2y4	459.28	481.24	64.6
794	CPE	P16870	LLIPGNYK	+2y3	459.28	424.22	64.6
795	CPE	P16870	LTASAPGYLAITK	+2y11	653.37	1091.61	78.7
796	CPE	P16870	LTASAPGYLAITK	+2y10	653.37	1020.57	78.7
797	CPE	P16870	LTASAPGYLAITK	+2y9	653.37	933.54	78.7
798	CPE	P16870	LTASAPGYLAITK	+2y8	653.37	862.50	78.7
799	CPE	P16870	LTASAPGYLAITK	+2y4	653.37	432.28	78.7
800	CP	P00450	ALYLQYTDETFR	+2y9	760.38	1172.56	86.5
801	CP	P00450	ALYLQYTDETFR	+2y8	760.38	1059.47	86.5
802	CP	P00450	ALYLQYTDETFR	+2y7	760.38	931.42	86.5
803	CP	P00450	ALYLQYTDETFR	+2y6	760.38	768.35	86.5
804	CP	P00450	ALYLQYTDETFR	+2y4	760.38	552.28	86.5
805	CP	P00450	EYTDASFTNR	+2y8	602.27	911.42	75
806	CP	P00450	EYTDASFTNR	+2y7	602.27	810.37	75
807	CP	P00450	EYTDASFTNR	+2y6	602.27	695.35	75
808	CP	P00450	EYTDASFTNR	+2y5	602.27	624.31	75
809	CP	P00450	EYTDASFTNR	+2y4	602.27	537.28	75
810	CP	P00450	NNEGTYYSPNYNPQSR	+2y10	952.41	1225.56	100.5
811	CP	P00450	NNEGTYYSPNYNPQSR	+2y9	952.41	1062.50	100.5
812	CP	P00450	NNEGTYYSPNYNPQSR	+2y8	952.41	975.46	100.5
813	CP	P00450	NNEGTYYSPNYNPQSR	+2y5	952.41	601.31	100.5
814	CP	P00450	NNEGTYYSPNYNPQSR	+2y4	952.41	487.26	100.5
815	CP	P00450	EDEDFQESNK	+2y7	620.75	867.38	76.4
816	CP	P00450	EDEDFQESNK	+2y6	620.75	752.36	76.4
817	CP	P00450	EDEDFQESNK	+2y5	620.75	605.29	76.4
818	CP	P00450	EDEDFQESNK	+2y4	620.75	477.23	76.4
819	CP	P00450	EDEDFQESNK	+2y3	620.75	348.19	76.4
820	CP	P00450	DDEEFIESNK	+2y7	613.26	866.43	75.8
821	CP	P00450	DDEEFIESNK	+2y6	613.26	737.38	75.8
822	CP	P00450	DDEEFIESNK	+2y5	613.26	590.31	75.8
823	CP	P00450	DDEEFIESNK	+2y4	613.26	477.23	75.8
824	CP	P00450	DDEEFIESNK	+2y3	613.26	348.19	75.8
825	C4B	P0C0L5	VDFTLSSER	+2y8	527.26	954.45	69.6
826	C4B	P0C0L5	VDFTLSSER	+2y7	527.26	839.43	69.6
827	C4B	P0C0L5	VDFTLSSER	+2y6	527.26	692.36	69.6
828	C4B	P0C0L5	VDFTLSSER	+2y5	527.26	591.31	69.6
829	C4B	P0C0L5	VDFTLSSER	+2y4	527.26	478.23	69.6
830	C4B	P0C0L5	TTNIQGINLLFSSR	+2y10	782.43	1134.63	88.2
831	C4B	P0C0L5	TTNIQGINLLFSSR	+2y9	782.43	1006.57	88.2
832	C4B	P0C0L5	TTNIQGINLLFSSR	+2y7	782.43	836.46	88.2
833	C4B	P0C0L5	TTNIQGINLLFSSR	+2y4	782.43	496.25	88.2
834	C4B	P0C0L5	TTNIQGINLLFSSR	+2y3	782.43	349.18	88.2
835	C4B	P0C0L5	FEQLELRPVLYNYLDK	+2y11	1020.54	1393.78	105.5
836	C4B	P0C0L5	FEQLELRPVLYNYLDK	+2y9	1020.54	1124.60	105.5
837	C4B	P0C0L5	FEQLELRPVLYNYLDK	+2y7	1020.54	928.48	105.5

838	C4B	P0C0L5	FEQLELRPVLYNYLDK	+2y6	1020.54	815.39	105.5
839	C4B	P0C0L5	FEQLELRPVLYNYLDK	+2y5	1020.54	652.33	105.5
840	C4B	P0C0L5	VLSLAQEQVGGSPEK	+2y11	771.41	1129.55	87.4
841	C4B	P0C0L5	VLSLAQEQVGGSPEK	+2y10	771.41	1058.51	87.4
842	C4B	P0C0L5	VLSLAQEQVGGSPEK	+2y9	771.41	930.45	87.4
843	C4B	P0C0L5	VLSLAQEQVGGSPEK	+2y6	771.41	574.28	87.4
844	C4B	P0C0L5	VLSLAQEQVGGSPEK	+2y3	771.41	373.21	87.4
845	C4B	P0C0L5	GLEEELQFSLGSK	+2y8	718.87	879.49	83.5
846	C4B	P0C0L5	GLEEELQFSLGSK	+2y7	718.87	766.41	83.5
847	C4B	P0C0L5	GLEEELQFSLGSK	+2y6	718.87	638.35	83.5
848	C4B	P0C0L5	GLEEELQFSLGSK	+2y5	718.87	491.28	83.5
849	C4B	P0C0L5	GLEEELQFSLGSK	+2y4	718.87	404.25	83.5
850	SERPINA6	P08185	GLASANVDFAFSLYK	+2y10	801.91	1203.60	89.6
851	SERPINA6	P08185	GLASANVDFAFSLYK	+2y9	801.91	1089.56	89.6
852	SERPINA6	P08185	GLASANVDFAFSLYK	+2y8	801.91	990.49	89.6
853	SERPINA6	P08185	GLASANVDFAFSLYK	+2y7	801.91	875.47	89.6
854	SERPINA6	P08185	GLASANVDFAFSLYK	+2y4	801.91	510.29	89.6
855	SERPINA6	P08185	GTWTQPFDLASTR	+2y10	740.36	1135.57	85.1
856	SERPINA6	P08185	GTWTQPFDLASTR	+2y9	740.36	1034.53	85.1
857	SERPINA6	P08185	GTWTQPFDLASTR	+2y8	740.36	906.47	85.1
858	SERPINA6	P08185	GTWTQPFDLASTR	+2y5	740.36	547.32	85.1
859	SERPINA6	P08185	GTWTQPFDLASTR	+2y4	740.36	434.24	85.1
860	SERPINA6	P08185	EENFYVDETTVVK	+2y9	786.88	1053.55	88.5
861	SERPINA6	P08185	EENFYVDETTVVK	+2y8	786.88	890.48	88.5
862	SERPINA6	P08185	EENFYVDETTVVK	+2y7	786.88	791.41	88.5
863	SERPINA6	P08185	EENFYVDETTVVK	+2y6	786.88	676.39	88.5
864	SERPINA6	P08185	EENFYVDETTVVK	+2y3	786.88	345.25	88.5
865	SERPINA6	P08185	WSAGLTSSQVDLYIPK	+2y11	882.96	1250.66	95.5
866	SERPINA6	P08185	WSAGLTSSQVDLYIPK	+2y10	882.96	1149.62	95.5
867	SERPINA6	P08185	WSAGLTSSQVDLYIPK	+2y7	882.96	847.49	95.5
868	SERPINA6	P08185	WSAGLTSSQVDLYIPK	+2y6	882.96	748.42	95.5
869	SERPINA6	P08185	WSAGLTSSQVDLYIPK	+2y3	882.96	357.25	95.5
870	SERPINA6	P08185	ITQDAQLK	+2y7	458.76	803.43	64.6
871	SERPINA6	P08185	ITQDAQLK	+2y6	458.76	702.38	64.6
872	SERPINA6	P08185	ITQDAQLK	+2y5	458.76	574.32	64.6
873	SERPINA6	P08185	ITQDAQLK	+2y3	458.76	388.26	64.6
874	FGB	P02675	DNENVVNEYSSELEK	+2y10	884.90	1197.56	95.6
875	FGB	P02675	DNENVVNEYSSELEK	+2y9	884.90	1098.49	95.6
876	FGB	P02675	DNENVVNEYSSELEK	+2y7	884.90	855.41	95.6
877	FGB	P02675	DNENVVNEYSSELEK	+2y6	884.90	692.35	95.6
878	FGB	P02675	DNENVVNEYSSELEK	+2y5	884.90	605.31	95.6
879	FGB	P02675	QDGSVDFGR	+2y8	490.73	852.38	66.9
880	FGB	P02675	QDGSVDFGR	+2y7	490.73	737.36	66.9
881	FGB	P02675	QDGSVDFGR	+2y5	490.73	593.30	66.9
882	FGB	P02675	QDGSVDFGR	+2y4	490.73	494.24	66.9
883	FGB	P02675	QDGSVDFGR	+2y3	490.73	379.21	66.9
884	FGB	P02675	QGFGNVATNTDGK	+2y7	654.81	706.34	78.9
885	FGB	P02675	QGFGNVATNTDGK	+2y6	654.81	635.30	78.9
886	FGB	P02675	QGFGNVATNTDGK	+2y5	654.81	534.25	78.9
887	FGB	P02675	QGFGNVATNTDGK	+2y4	654.81	420.21	78.9

888	FGB	P02675	QGFGNVATNTDGK	+2y3	654.81	319.16	78.9
889	FGB	P02675	EDGGGWWYNR	+2y8	620.26	995.45	76.3
890	FGB	P02675	EDGGGWWYNR	+2y6	620.26	881.41	76.3
891	FGB	P02675	EDGGGWWYNR	+2y5	620.26	824.38	76.3
892	FGB	P02675	EDGGGWWYNR	+2y4	620.26	638.30	76.3
893	FGB	P02675	EDGGGWWYNR	+2y3	620.26	452.23	76.3
894	FGB	P02675	IRPFFPQQ	+2y4	516.78	519.26	68.8
895	FGB	P02675	IRPFFPQQ	+2y3	516.78	372.19	68.8
896	FN1	P02751	NTFAEVTGLSPGVTYYFK	+2y12	997.50	1332.68	103.8
897	FN1	P02751	NTFAEVTGLSPGVTYYFK	+2y11	997.50	1231.64	103.8
898	FN1	P02751	NTFAEVTGLSPGVTYYFK	+2y9	997.50	1061.53	103.8
899	FN1	P02751	NTFAEVTGLSPGVTYYFK	+2y8	997.50	974.50	103.8
900	FN1	P02751	NTFAEVTGLSPGVTYYFK	+2y5	997.50	721.36	103.8
901	FN1	P02751	LDAPTNLQFVNETDSTVLVR	+2y12	1116.58	1379.72	112.5
902	FN1	P02751	LDAPTNLQFVNETDSTVLVR	+2y11	1116.58	1232.65	112.5
903	FN1	P02751	LDAPTNLQFVNETDSTVLVR	+2y9	1116.58	1019.54	112.5
904	FN1	P02751	LDAPTNLQFVNETDSTVLVR	+2y8	1116.58	890.49	112.5
905	FN1	P02751	LDAPTNLQFVNETDSTVLVR	+2y6	1116.58	674.42	112.5
906	FN1	P02751	VTDATETTITISWR	+2y10	797.41	1207.63	89.2
907	FN1	P02751	VTDATETTITISWR	+2y8	797.41	977.54	89.2
908	FN1	P02751	VTDATETTITISWR	+2y7	797.41	876.49	89.2
909	FN1	P02751	VTDATETTITISWR	+2y4	797.41	561.31	89.2
910	FN1	P02751	VTDATETTITISWR	+2y3	797.41	448.23	89.2
911	FN1	P02751	SSPVVIDASTAIDAPSNLR	+2y9	957.00	956.52	100.9
912	FN1	P02751	SSPVVIDASTAIDAPSNLR	+2y8	957.00	885.48	100.9
913	FN1	P02751	SSPVVIDASTAIDAPSNLR	+2y7	957.00	772.39	100.9
914	FN1	P02751	SSPVVIDASTAIDAPSNLR	+2y6	957.00	657.37	100.9
915	FN1	P02751	SSPVVIDASTAIDAPSNLR	+2y5	957.00	586.33	100.9
916	FN1	P02751	FLATTPNSLLVSWQPPR	+2y12	964.03	1393.76	101.4
917	FN1	P02751	FLATTPNSLLVSWQPPR	+2y8	964.03	982.55	101.4
918	FN1	P02751	FLATTPNSLLVSWQPPR	+2y6	964.03	770.39	101.4
919	FN1	P02751	FLATTPNSLLVSWQPPR	+2y4	964.03	497.28	101.4
920	FN1	P02751	FLATTPNSLLVSWQPPR	+2y3	964.03	369.22	101.4
921	ORM1	P02763	WFYIASAFR	+2y8	580.80	974.51	73.5
922	ORM1	P02763	WFYIASAFR	+2y7	580.80	827.44	73.5
923	ORM1	P02763	WFYIASAFR	+2y6	580.80	664.38	73.5
924	ORM1	P02763	WFYIASAFR	+2y5	580.80	551.29	73.5
925	ORM1	P02763	WFYIASAFR	+2y4	580.80	480.26	73.5
926	ORM1	P02763	SVQEIQATFFYFTPNK	+2y11	960.48	1363.67	101.1
927	ORM1	P02763	SVQEIQATFFYFTPNK	+2y10	960.48	1235.61	101.1
928	ORM1	P02763	SVQEIQATFFYFTPNK	+2y8	960.48	1063.52	101.1
929	ORM1	P02763	SVQEIQATFFYFTPNK	+2y7	960.48	916.46	101.1
930	ORM1	P02763	SVQEIQATFFYFTPNK	+2y4	960.48	459.26	101.1
931	ORM1	P02763	TEDTIFLR	+2y7	497.76	893.47	67.4
932	ORM1	P02763	TEDTIFLR	+2y6	497.76	764.43	67.4
933	ORM1	P02763	TEDTIFLR	+2y5	497.76	649.40	67.4
934	ORM1	P02763	TEDTIFLR	+2y4	497.76	548.36	67.4
935	ORM1	P02763	TEDTIFLR	+2y3	497.76	435.27	67.4
936	ORM1	P02763	NWGLSVYADKPETTK	+2y11	854.93	1238.63	93.4
937	ORM1	P02763	NWGLSVYADKPETTK	+2y9	854.93	1052.53	93.4

938	ORM1	P02763	NWGLSVYADKPETTK	+2y8	854.93	889.46	93.4
939	ORM1	P02763	NWGLSVYADKPETTK	+2y6	854.93	703.40	93.4
940	ORM1	P02763	NWGLSVYADKPETTK	+2y5	854.93	575.30	93.4
941	ORM1	P02763	SDVVYTDWK	+2y6	556.77	811.40	71.7
942	ORM1	P02763	SDVVYTDWK	+2y5	556.77	712.33	71.7
943	ORM1	P02763	SDVVYTDWK	+2y4	556.77	549.27	71.7
944	ORM1	P02763	SDVVYTDWK	+2y3	556.77	448.22	71.7
945	ORM1	P02763	SDVVYTDWK	+2y2	556.77	333.19	71.7
946	SERPINA4	P29622	FYYLIASETPGK	+2y8	694.86	802.43	81.8
947	SERPINA4	P29622	FYYLIASETPGK	+2y7	694.86	689.35	81.8
948	SERPINA4	P29622	FYYLIASETPGK	+2y6	694.86	618.31	81.8
949	SERPINA4	P29622	FYYLIASETPGK	+2y4	694.86	402.23	81.8
950	SERPINA4	P29622	FYYLIASETPGK	+2y3	694.86	301.19	81.8
951	SERPINA4	P29622	DFYVDENTTVR	+2y9	679.81	1096.53	80.7
952	SERPINA4	P29622	DFYVDENTTVR	+2y8	679.81	933.46	80.7
953	SERPINA4	P29622	DFYVDENTTVR	+2y7	679.81	834.40	80.7
954	SERPINA4	P29622	DFYVDENTTVR	+2y6	679.81	719.37	80.7
955	SERPINA4	P29622	DFYVDENTTVR	+2y5	679.81	590.33	80.7
956	SERPINA4	P29622	GDATVFFILPNQGK	+2y9	753.90	1063.59	86.1
957	SERPINA4	P29622	GDATVFFILPNQGK	+2y8	753.90	916.53	86.1
958	SERPINA4	P29622	GDATVFFILPNQGK	+2y7	753.90	769.46	86.1
959	SERPINA4	P29622	GDATVFFILPNQGK	+2y6	753.90	656.37	86.1
960	SERPINA4	P29622	GDATVFFILPNQGK	+2y5	753.90	543.29	86.1
961	SERPINA4	P29622	FSISGSYVLDQILPR	+2y12	847.96	1347.73	92.9
962	SERPINA4	P29622	FSISGSYVLDQILPR	+2y8	847.96	953.58	92.9
963	SERPINA4	P29622	FSISGSYVLDQILPR	+2y7	847.96	854.51	92.9
964	SERPINA4	P29622	FSISGSYVLDQILPR	+2y6	847.96	741.43	92.9
965	SERPINA4	P29622	FSISGSYVLDQILPR	+2y3	847.96	385.26	92.9
966	SERPINA4	P29622	LGFTDLFSK	+2y8	514.28	914.46	68.6
967	SERPINA4	P29622	LGFTDLFSK	+2y7	514.28	857.44	68.6
968	SERPINA4	P29622	LGFTDLFSK	+2y6	514.28	710.37	68.6
969	SERPINA4	P29622	LGFTDLFSK	+2y5	514.28	609.32	68.6
970	SERPINA4	P29622	LGFTDLFSK	+2y3	514.28	381.21	68.6
971	AMBP	P02760	ETLLQDFR	+2y6	511.27	791.44	68.4
972	AMBP	P02760	ETLLQDFR	+2y5	511.27	678.36	68.4
973	AMBP	P02760	ETLLQDFR	+2y4	511.27	565.27	68.4
974	AMBP	P02760	ETLLQDFR	+2y3	511.27	437.21	68.4
975	AMBP	P02760	ETLLQDFR	+2y2	511.27	322.19	68.4
976	AMBP	P02760	AFIQLWAFDAVK	+2y8	704.88	949.51	82.5
977	AMBP	P02760	AFIQLWAFDAVK	+2y7	704.88	836.43	82.5
978	AMBP	P02760	AFIQLWAFDAVK	+2y6	704.88	650.35	82.5
979	AMBP	P02760	AFIQLWAFDAVK	+2y5	704.88	579.31	82.5
980	AMBP	P02760	AFIQLWAFDAVK	+2y4	704.88	432.25	82.5
981	CLU	P10909	EIQNAVNGVK	+2y8	536.29	829.45	70.2
982	CLU	P10909	EIQNAVNGVK	+2y7	536.29	701.39	70.2
983	CLU	P10909	EIQNAVNGVK	+2y6	536.29	587.35	70.2
984	CLU	P10909	EIQNAVNGVK	+2y5	536.29	516.31	70.2
985	CLU	P10909	EIQNAVNGVK	+2y4	536.29	417.25	70.2
986	CLU	P10909	ASSIIDELFQDR	+2y8	697.35	1035.51	82
987	CLU	P10909	ASSIIDELFQDR	+2y7	697.35	922.43	82

988	CLU	P10909	ASSIIDELFQDR	+2y6	697.35	807.40	82
989	CLU	P10909	ASSIIDELFQDR	+2y5	697.35	678.36	82
990	CLU	P10909	ASSIIDELFQDR	+2y4	697.35	565.27	82
991	CLU	P10909	ELDESLQVAER	+2y9	644.82	1046.51	78.1
992	CLU	P10909	ELDESLQVAER	+2y7	644.82	802.44	78.1
993	CLU	P10909	ELDESLQVAER	+2y5	644.82	602.33	78.1
994	CLU	P10909	ELDESLQVAER	+2y4	644.82	474.27	78.1
995	CLU	P10909	ELDESLQVAER	+2y3	644.82	375.20	78.1
996	CLU	P10909	LANLTQGEDQYYLR	+2y10	842.42	1272.59	92.5
997	CLU	P10909	LANLTQGEDQYYLR	+2y9	842.42	1171.54	92.5
998	CLU	P10909	LANLTQGEDQYYLR	+2y8	842.42	1043.48	92.5
999	CLU	P10909	LANLTQGEDQYYLR	+2y6	842.42	857.42	92.5
1000	CLU	P10909	LANLTQGEDQYYLR	+2y5	842.42	742.39	92.5
1001	CLU	P10909	LFDSDPITVTVPVEVSR	+2y12	937.50	1296.75	99.5
1002	CLU	P10909	LFDSDPITVTVPVEVSR	+2y10	937.50	1086.62	99.5
1003	CLU	P10909	LFDSDPITVTVPVEVSR	+2y9	937.50	985.57	99.5
1004	CLU	P10909	LFDSDPITVTVPVEVSR	+2y8	937.50	886.50	99.5
1005	CLU	P10909	LFDSDPITVTVPVEVSR	+2y6	937.50	686.38	99.5
1006	CLEC3B	E9PHK0	NWETEITAQPDGGK	+2y11	773.36	1116.55	87.5
1007	CLEC3B	E9PHK0	NWETEITAQPDGGK	+2y8	773.36	773.38	87.5
1008	CLEC3B	E9PHK0	NWETEITAQPDGGK	+2y7	773.36	672.33	87.5
1009	CLEC3B	E9PHK0	NWETEITAQPDGGK	+2y6	773.36	601.29	87.5
1010	CLEC3B	E9PHK0	NWETEITAQPDGGK	+2y5	773.36	473.24	87.5
1011	ITIH4	Q14624	NGIDIYSLTVDSR	+2y10	726.87	1168.58	84.1
1012	ITIH4	Q14624	NGIDIYSLTVDSR	+2y8	726.87	940.47	84.1
1013	ITIH4	Q14624	NGIDIYSLTVDSR	+2y7	726.87	777.41	84.1
1014	ITIH4	Q14624	NGIDIYSLTVDSR	+2y6	726.87	690.38	84.1
1015	ITIH4	Q14624	NGIDIYSLTVDSR	+2y5	726.87	577.29	84.1
1016	ITIH4	Q14624	AEAQAQYSAAVAK	+2y9	654.33	908.48	78.8
1017	ITIH4	Q14624	AEAQAQYSAAVAK	+2y8	654.33	837.45	78.8
1018	ITIH4	Q14624	AEAQAQYSAAVAK	+2y7	654.33	709.39	78.8
1019	ITIH4	Q14624	AEAQAQYSAAVAK	+2y6	654.33	546.32	78.8
1020	ITIH4	Q14624	AEAQAQYSAAVAK	+2y4	654.33	388.26	78.8
1021	ITIH4	Q14624	ITFELVYEELLK	+2y10	748.92	1282.69	85.7
1022	ITIH4	Q14624	ITFELVYEELLK	+2y8	748.92	1006.58	85.7
1023	ITIH4	Q14624	ITFELVYEELLK	+2y7	748.92	893.50	85.7
1024	ITIH4	Q14624	ITFELVYEELLK	+2y6	748.92	794.43	85.7
1025	ITIH4	Q14624	ITFELVYEELLK	+2y5	748.92	631.37	85.7
1026	ITIH4	Q14624	GPDVLTATVSGK	+2y9	572.81	875.52	72.9
1027	ITIH4	Q14624	GPDVLTATVSGK	+2y8	572.81	776.45	72.9
1028	ITIH4	Q14624	GPDVLTATVSGK	+2y7	572.81	663.37	72.9
1029	ITIH4	Q14624	GPDVLTATVSGK	+2y6	572.81	562.32	72.9
1030	ITIH4	Q14624	GPDVLTATVSGK	+2y5	572.81	491.28	72.9
1031	ITIH4	Q14624	AGFSWIEVTFK	+2y8	642.83	1009.54	78
1032	ITIH4	Q14624	AGFSWIEVTFK	+2y7	642.83	922.50	78
1033	ITIH4	Q14624	AGFSWIEVTFK	+2y6	642.83	736.42	78
1034	ITIH4	Q14624	AGFSWIEVTFK	+2y5	642.83	623.34	78
1035	ITIH4	Q14624	AGFSWIEVTFK	+2y3	642.83	395.23	78
1036	LGALS3BP	Q08380	ALGFENATQALGR	+2y11	674.35	1163.58	80.3
1037	LGALS3BP	Q08380	ALGFENATQALGR	+2y7	674.35	716.40	80.3

1038	LGALS3BP	Q08380	ALGFENATQALGR	+2y6	674.35	645.37	80.3
1039	LGALS3BP	Q08380	ALGFENATQALGR	+2y5	674.35	544.32	80.3
1040	LGALS3BP	Q08380	ALGFENATQALGR	+2y4	674.35	416.26	80.3
1041	LGALS3BP	Q08380	ELSEALGQIFDSQR	+2y9	796.90	1063.55	89.2
1042	LGALS3BP	Q08380	ELSEALGQIFDSQR	+2y8	796.90	950.47	89.2
1043	LGALS3BP	Q08380	ELSEALGQIFDSQR	+2y6	796.90	765.39	89.2
1044	LGALS3BP	Q08380	ELSEALGQIFDSQR	+2y5	796.90	652.30	89.2
1045	LGALS3BP	Q08380	ELSEALGQIFDSQR	+2y3	796.90	390.21	89.2
1046	LGALS3BP	Q08380	SDLAVPSELALLK	+2y9	678.39	969.60	80.6
1047	LGALS3BP	Q08380	SDLAVPSELALLK	+2y8	678.39	870.53	80.6
1048	LGALS3BP	Q08380	SDLAVPSELALLK	+2y5	678.39	557.40	80.6
1049	LGALS3BP	Q08380	SDLAVPSELALLK	+2y4	678.39	444.32	80.6
1050	LGALS3BP	Q08380	SDLAVPSELALLK	+2y3	678.39	373.28	80.6
1051	LGALS3BP	Q08380	GLNLTEDTYKPR	+2y8	703.87	1009.49	82.4
1052	LGALS3BP	Q08380	GLNLTEDTYKPR	+2y7	703.87	908.45	82.4
1053	LGALS3BP	Q08380	GLNLTEDTYKPR	+2y6	703.87	779.40	82.4
1054	LGALS3BP	Q08380	GLNLTEDTYKPR	+2y5	703.87	664.38	82.4
1055	LGALS3BP	Q08380	GLNLTEDTYKPR	+2y4	703.87	563.33	82.4
1056	LGALS3BP	Q08380	IYTSPTWSAFVTDSSWSAR	+2y12	1081.51	1313.61	110
1057	LGALS3BP	Q08380	IYTSPTWSAFVTDSSWSAR	+2y10	1081.51	1155.54	110
1058	LGALS3BP	Q08380	IYTSPTWSAFVTDSSWSAR	+2y9	1081.51	1008.47	110
1059	LGALS3BP	Q08380	IYTSPTWSAFVTDSSWSAR	+2y8	1081.51	909.41	110
1060	LGALS3BP	Q08380	IYTSPTWSAFVTDSSWSAR	+2y6	1081.51	693.33	110
1061	ENPP2	Q13822	WWGGQPLWITATK	+2y11	772.41	1171.65	87.4
1062	ENPP2	Q13822	WWGGQPLWITATK	+2y8	772.41	929.55	87.4
1063	ENPP2	Q13822	WWGGQPLWITATK	+2y6	772.41	719.41	87.4
1064	ENPP2	Q13822	WWGGQPLWITATK	+2y5	772.41	533.33	87.4
1065	ENPP2	Q13822	WWGGQPLWITATK	+2y4	772.41	420.25	87.4
1066	ENPP2	Q13822	VWNYFQR	+2y6	506.75	913.43	68.1
1067	ENPP2	Q13822	VWNYFQR	+2y5	506.75	727.35	68.1
1068	ENPP2	Q13822	VWNYFQR	+2y4	506.75	613.31	68.1
1069	ENPP2	Q13822	VWNYFQR	+2y3	506.75	450.25	68.1
1070	SERPINA5	P05154	TLYLADTFPTNFR	+2y9	779.90	1068.51	88
1071	SERPINA5	P05154	TLYLADTFPTNFR	+2y8	779.90	997.47	88
1072	SERPINA5	P05154	TLYLADTFPTNFR	+2y7	779.90	882.45	88
1073	SERPINA5	P05154	TLYLADTFPTNFR	+2y6	779.90	781.40	88
1074	SERPINA5	P05154	TLYLADTFPTNFR	+2y5	779.90	634.33	88
1075	SERPINA5	P05154	GTQEQDFYVTSETVVR	+2y10	929.94	1200.63	98.9
1076	SERPINA5	P05154	GTQEQDFYVTSETVVR	+2y9	929.94	1053.56	98.9
1077	SERPINA5	P05154	GTQEQDFYVTSETVVR	+2y8	929.94	890.49	98.9
1078	SERPINA5	P05154	GTQEQDFYVTSETVVR	+2y7	929.94	791.43	98.9
1079	SERPINA5	P05154	GTQEQDFYVTSETVVR	+2y6	929.94	690.38	98.9
1080	SERPINA5	P05154	QLELYLPK	+2y6	502.29	762.44	67.7
1081	SERPINA5	P05154	QLELYLPK	+2y5	502.29	633.40	67.7
1082	SERPINA5	P05154	QLELYLPK	+2y4	502.29	520.31	67.7
1083	SERPINA5	P05154	QLELYLPK	+2y3	502.29	357.25	67.7
1084	SERPINA5	P05154	FSIEGSYQLEK	+2y9	650.82	1066.54	78.6
1085	SERPINA5	P05154	FSIEGSYQLEK	+2y8	650.82	953.46	78.6
1086	SERPINA5	P05154	FSIEGSYQLEK	+2y7	650.82	824.41	78.6
1087	SERPINA5	P05154	FSIEGSYQLEK	+2y6	650.82	767.39	78.6

1088	SERPINA5	P05154	FSIEGSYQLEK	+2y5	650.82	680.36	78.6
1089	SERPINA5	P05154	AAAATGTIFTFR	+2y8	613.83	942.50	75.9
1090	SERPINA5	P05154	AAAATGTIFTFR	+2y7	613.83	841.46	75.9
1091	SERPINA5	P05154	AAAATGTIFTFR	+2y4	613.83	570.30	75.9
1092	CST3	P01034	ALDFAVGEYNK	+2y9	613.81	1042.48	75.9
1093	CST3	P01034	ALDFAVGEYNK	+2y7	613.81	780.39	75.9
1094	CST3	P01034	ALDFAVGEYNK	+2y6	613.81	709.35	75.9
1095	CST3	P01034	ALDFAVGEYNK	+2y5	613.81	610.28	75.9
1096	CST3	P01034	ALDFAVGEYNK	+2y3	613.81	424.22	75.9
1097	CST3	P01034	QIVAGVNYFLDVELGR	+2y12	896.98	1381.71	96.5
1098	CST3	P01034	QIVAGVNYFLDVELGR	+2y10	896.98	1225.62	96.5
1099	CST3	P01034	QIVAGVNYFLDVELGR	+2y9	896.98	1111.58	96.5
1100	CST3	P01034	QIVAGVNYFLDVELGR	+2y8	896.98	948.51	96.5
1101	CST3	P01034	QIVAGVNYFLDVELGR	+2y6	896.98	688.36	96.5
1102	OPTC	Q9UBM4	EGDSFEVLPLR	+2y7	631.32	873.52	77.1
1103	OPTC	Q9UBM4	EGDSFEVLPLR	+2y6	631.32	726.45	77.1
1104	OPTC	Q9UBM4	EGDSFEVLPLR	+2y5	631.32	597.41	77.1
1105	OPTC	Q9UBM4	EGDSFEVLPLR	+2y4	631.32	498.34	77.1
1106	OPTC	Q9UBM4	EGDSFEVLPLR	+2y3	631.32	385.26	77.1
1107	DKK3	Q9UBP4	LLDLITWELEPDGALDR	+2y11	985.02	1300.62	102.9
1108	DKK3	Q9UBP4	LLDLITWELEPDGALDR	+2y10	985.02	1114.54	102.9
1109	DKK3	Q9UBP4	LLDLITWELEPDGALDR	+2y8	985.02	872.41	102.9
1110	DKK3	Q9UBP4	LLDLITWELEPDGALDR	+2y7	985.02	743.37	102.9
1111	DKK3	Q9UBP4	LLDLITWELEPDGALDR	+2y5	985.02	531.29	102.9
1112	DKK3	Q9UBP4	DQDGEILLPR	+2y8	578.30	912.51	73.3
1113	DKK3	Q9UBP4	DQDGEILLPR	+2y7	578.30	797.49	73.3
1114	DKK3	Q9UBP4	DQDGEILLPR	+2y5	578.30	611.42	73.3
1115	DKK3	Q9UBP4	DQDGEILLPR	+2y4	578.30	498.34	73.3
1116	DKK3	Q9UBP4	DQDGEILLPR	+2y3	578.30	385.26	73.3
1117	DKK3	Q9UBP4	QELEDLER	+2y6	516.25	774.40	68.8
1118	DKK3	Q9UBP4	QELEDLER	+2y5	516.25	661.32	68.8
1119	DKK3	Q9UBP4	QELEDLER	+2y4	516.25	532.27	68.8
1120	DKK3	Q9UBP4	QELEDLER	+2y3	516.25	417.25	68.8
1121	DKK3	Q9UBP4	QELEDLER	+2y2	516.25	304.16	68.8
1122	DKK3	Q9UBP4	EPAAAAAALLGGEEI	+2y11	691.86	1014.55	81.6
1123	DKK3	Q9UBP4	EPAAAAAALLGGEEI	+2y8	691.86	801.44	81.6
1124	DKK3	Q9UBP4	EPAAAAAALLGGEEI	+2y7	691.86	730.40	81.6
1125	DKK3	Q9UBP4	EPAAAAAALLGGEEI	+2y6	691.86	617.31	81.6
1126	DKK3	Q9UBP4	EPAAAAAALLGGEEI	+2y5	691.86	504.23	81.6
1127	TPP1	O14773	ADPEEELSLTFALR	+2y9	795.90	1049.60	89.1
1128	TPP1	O14773	ADPEEELSLTFALR	+2y8	795.90	920.56	89.1
1129	TPP1	O14773	ADPEEELSLTFALR	+2y6	795.90	720.44	89.1
1130	TPP1	O14773	ADPEEELSLTFALR	+2y5	795.90	607.36	89.1
1131	TPP1	O14773	ADPEEELSLTFALR	+2y4	795.90	506.31	89.1
1132	TPP1	O14773	LSELVQAVSDPSSPQYGK	+2y11	952.98	1164.55	100.6
1133	TPP1	O14773	LSELVQAVSDPSSPQYGK	+2y10	952.98	1065.48	100.6
1134	TPP1	O14773	LSELVQAVSDPSSPQYGK	+2y9	952.98	978.45	100.6
1135	TPP1	O14773	LSELVQAVSDPSSPQYGK	+2y8	952.98	863.43	100.6
1136	TPP1	O14773	LSELVQAVSDPSSPQYGK	+2y5	952.98	592.31	100.6
1137	TPP1	O14773	AYPDVAALSDGYWVVSNR	+2y10	991.98	1182.55	103.4

1138	TPP1	O14773	AYPDVAALSDGYWVVSNR	+2y9	991.98	1095.52	103.4
1139	TPP1	O14773	AYPDVAALSDGYWVVSNR	+2y8	991.98	980.49	103.4
1140	TPP1	O14773	AYPDVAALSDGYWVVSNR	+2y6	991.98	760.41	103.4
1141	TPP1	O14773	AYPDVAALSDGYWVVSNR	+2y4	991.98	475.26	103.4
1142	TPP1	O14773	ILSGRPPLGFLNPR	+2y9	768.95	1010.58	87.2
1143	TPP1	O14773	ILSGRPPLGFLNPR	+2y8	768.95	913.53	87.2
1144	TPP1	O14773	ILSGRPPLGFLNPR	+2y6	768.95	703.39	87.2
1145	TPP1	O14773	ILSGRPPLGFLNPR	+2y5	768.95	646.37	87.2
1146	TPP1	O14773	ILSGRPPLGFLNPR	+2y4	768.95	499.30	87.2
1147	ABI3BP	Q7Z7G0	FTEAIVDAEPK	+2y9	610.31	971.50	75.6
1148	ABI3BP	Q7Z7G0	FTEAIVDAEPK	+2y8	610.31	842.46	75.6
1149	ABI3BP	Q7Z7G0	FTEAIVDAEPK	+2y7	610.31	771.42	75.6
1150	ABI3BP	Q7Z7G0	FTEAIVDAEPK	+2y6	610.31	658.34	75.6
1151	ABI3BP	Q7Z7G0	FTEAIVDAEPK	+2y5	610.31	559.27	75.6
1152	ABI3BP	Q7Z7G0	YLIVVRPAPPPSQK	+2y11	782.96	1175.69	88.2
1153	ABI3BP	Q7Z7G0	YLIVVRPAPPPSQK	+2y10	782.96	1076.62	88.2
1154	ABI3BP	Q7Z7G0	YLIVVRPAPPPSQK	+2y9	782.96	977.55	88.2
1155	ABI3BP	Q7Z7G0	YLIVVRPAPPPSQK	+2y8	782.96	821.45	88.2
1156	ABI3BP	Q7Z7G0	YLIVVRPAPPPSQK	+2y6	782.96	653.36	88.2
1157	ABI3BP	Q7Z7G0	DNVEGGIWSK	+2y8	552.77	875.46	71.4
1158	ABI3BP	Q7Z7G0	DNVEGGIWSK	+2y7	552.77	776.39	71.4
1159	ABI3BP	Q7Z7G0	DNVEGGIWSK	+2y6	552.77	647.35	71.4
1160	ABI3BP	Q7Z7G0	DNVEGGIWSK	+2y5	552.77	590.33	71.4
1161	ABI3BP	Q7Z7G0	DNVEGGIWSK	+2y3	552.77	420.22	71.4
1162	ABI3BP	Q7Z7G0	FYNIGDQR	+2y6	506.75	702.35	68.1
1163	ABI3BP	Q7Z7G0	FYNIGDQR	+2y5	506.75	588.31	68.1
1164	ABI3BP	Q7Z7G0	FYNIGDQR	+2y4	506.75	475.23	68.1
1165	ABI3BP	Q7Z7G0	FYNIGDQR	+2y3	506.75	418.20	68.1
1166	ABI3BP	Q7Z7G0	FYNIGDQR	+2y2	506.75	303.18	68.1
1167	ABI3BP	Q7Z7G0	TGQQLTSDQLPIK	+2y10	714.89	1142.64	83.2
1168	ABI3BP	Q7Z7G0	TGQQLTSDQLPIK	+2y9	714.89	1014.58	83.2
1169	ABI3BP	Q7Z7G0	TGQQLTSDQLPIK	+2y8	714.89	901.50	83.2
1170	ABI3BP	Q7Z7G0	TGQQLTSDQLPIK	+2y7	714.89	800.45	83.2
1171	ABI3BP	Q7Z7G0	TGQQLTSDQLPIK	+2y3	714.89	357.25	83.2
1172	LRG1	P02750	GPLQLER	+2y5	406.73	658.39	60.8
1173	LRG1	P02750	GPLQLER	+2y4	406.73	545.30	60.8
1174	LRG1	P02750	GPLQLER	+2y3	406.73	417.25	60.8
1175	LRG1	P02750	GPLQLER	+2y2	406.73	304.16	60.8
1176	LRG1	P02750	DLLLPQPDLR	+2y8	590.34	951.56	74.2
1177	LRG1	P02750	DLLLPQPDLR	+2y7	590.34	838.48	74.2
1178	LRG1	P02750	DLLLPQPDLR	+2y6	590.34	725.39	74.2
1179	LRG1	P02750	DLLLPQPDLR	+2y5	590.34	628.34	74.2
1180	LRG1	P02750	DLLLPQPDLR	+2y4	590.34	500.28	74.2
1181	LRG1	P02750	YLFLNGNK	+2y7	484.76	805.46	66.5
1182	LRG1	P02750	YLFLNGNK	+2y6	484.76	692.37	66.5
1183	LRG1	P02750	YLFLNGNK	+2y5	484.76	545.30	66.5
1184	LRG1	P02750	YLFLNGNK	+2y4	484.76	432.22	66.5
1185	LRG1	P02750	YLFLNGNK	+2y3	484.76	318.18	66.5
1186	LRG1	P02750	VAAGAFQGLR	+2y9	495.28	890.48	67.2
1187	LRG1	P02750	VAAGAFQGLR	+2y8	495.28	819.45	67.2

1188	LRG1	P02750	VAAGAFQGLR	+2y7	495.28	748.41	67.2
1189	LRG1	P02750	VAAGAFQGLR	+2y5	495.28	620.35	67.2
1190	LRG1	P02750	VAAGAFQGLR	+2y3	495.28	345.22	67.2
1191	LRG1	P02750	GQTLLAVAK	+2y7	450.78	715.47	64
1192	LRG1	P02750	GQTLLAVAK	+2y6	450.78	614.42	64
1193	LRG1	P02750	GQTLLAVAK	+2y5	450.78	501.34	64
1194	LRG1	P02750	GQTLLAVAK	+2y4	450.78	388.26	64
1195	LRG1	P02750	GQTLLAVAK	+2y3	450.78	317.22	64
1196	SPON1	Q9HCB6	EGYTEFSLR	+2y7	551.26	915.46	71.3
1197	SPON1	Q9HCB6	EGYTEFSLR	+2y6	551.26	752.39	71.3
1198	SPON1	Q9HCB6	EGYTEFSLR	+2y5	551.26	651.35	71.3
1199	SPON1	Q9HCB6	EGYTEFSLR	+2y4	551.26	522.30	71.3
1200	SPON1	Q9HCB6	EGYTEFSLR	+2y3	551.26	375.24	71.3
1201	SPON1	Q9HCB6	GFTLIALR	+2y6	445.78	686.46	63.6
1202	SPON1	Q9HCB6	GFTLIALR	+2y5	445.78	585.41	63.6
1203	SPON1	Q9HCB6	GFTLIALR	+2y4	445.78	472.32	63.6
1204	SPON1	Q9HCB6	GFTLIALR	+2y3	445.78	359.24	63.6
1205	SPON1	Q9HCB6	IIYFQDEGSLTK	+2y10	707.37	1187.56	82.7
1206	SPON1	Q9HCB6	IIYFQDEGSLTK	+2y9	707.37	1024.49	82.7
1207	SPON1	Q9HCB6	IIYFQDEGSLTK	+2y8	707.37	877.43	82.7
1208	SPON1	Q9HCB6	IIYFQDEGSLTK	+2y7	707.37	749.37	82.7
1209	SPON1	Q9HCB6	IIYFQDEGSLTK	+2y6	707.37	634.34	82.7
1210	SPON1	Q9HCB6	NYVLWEYGGYASEGVK	+2y12	917.94	1345.61	98
1211	SPON1	Q9HCB6	NYVLWEYGGYASEGVK	+2y11	917.94	1159.53	98
1212	SPON1	Q9HCB6	NYVLWEYGGYASEGVK	+2y10	917.94	1030.48	98
1213	SPON1	Q9HCB6	NYVLWEYGGYASEGVK	+2y9	917.94	867.42	98
1214	SPON1	Q9HCB6	NYVLWEYGGYASEGVK	+2y8	917.94	810.40	98
1215	SPON1	Q9HCB6	AQWPAWQPLNVR	+2y10	733.39	1266.67	84.6
1216	SPON1	Q9HCB6	AQWPAWQPLNVR	+2y9	733.39	1080.59	84.6
1217	SPON1	Q9HCB6	AQWPAWQPLNVR	+2y7	733.39	912.51	84.6
1218	SPON1	Q9HCB6	AQWPAWQPLNVR	+2y6	733.39	726.43	84.6
1219	SPON1	Q9HCB6	AQWPAWQPLNVR	+2y5	733.39	598.37	84.6
1220	IGHA1	P01876	DASGVTFTWTPSSGK	+2y10	770.87	1111.54	87.3
1221	IGHA1	P01876	DASGVTFTWTPSSGK	+2y8	770.87	863.43	87.3
1222	IGHA1	P01876	DASGVTFTWTPSSGK	+2y7	770.87	762.38	87.3
1223	IGHA1	P01876	DASGVTFTWTPSSGK	+2y6	770.87	576.30	87.3
1224	IGHA1	P01876	DASGVTFTWTPSSGK	+2y5	770.87	475.25	87.3
1225	IGHA1	P01876	TPLTATLSK	+2y7	466.28	733.45	65.1
1226	IGHA1	P01876	TPLTATLSK	+2y6	466.28	620.36	65.1
1227	IGHA1	P01876	TPLTATLSK	+2y5	466.28	519.31	65.1
1228	IGHA1	P01876	TPLTATLSK	+2y4	466.28	448.28	65.1
1229	IGHA1	P01876	TPLTATLSK	+2y3	466.28	347.23	65.1
1230	IGHA1	P01876	WLQGSQELPR	+2y9	607.32	1027.55	75.4
1231	IGHA1	P01876	WLQGSQELPR	+2y8	607.32	914.47	75.4
1232	IGHA1	P01876	WLQGSQELPR	+2y7	607.32	786.41	75.4
1233	IGHA1	P01876	WLQGSQELPR	+2y4	607.32	514.30	75.4
1234	IGHA1	P01876	WLQGSQELPR	+2y3	607.32	385.26	75.4
1235	IGHA1	P01876	YLTWASR	+2y6	448.73	733.40	63.8
1236	IGHA1	P01876	YLTWASR	+2y5	448.73	620.32	63.8
1237	IGHA1	P01876	YLTWASR	+2y4	448.73	519.27	63.8

1238	IGHA1	P01876	YLTWASR	+2y3	448.73	333.19	63.8
1239	IGHA1	P01876	QEPSQGTTTFAVTSILR	+2y12	918.48	1266.71	98.1
1240	IGHA1	P01876	QEPSQGTTTFAVTSILR	+2y10	918.48	1108.64	98.1
1241	IGHA1	P01876	QEPSQGTTTFAVTSILR	+2y8	918.48	906.54	98.1
1242	IGHA1	P01876	QEPSQGTTTFAVTSILR	+2y7	918.48	759.47	98.1
1243	IGHA1	P01876	QEPSQGTTTFAVTSILR	+2y5	918.48	589.37	98.1
1244	RS1	O15537	FQDSSQWLQIDLK	+2y10	804.41	1217.65	89.8
1245	RS1	O15537	FQDSSQWLQIDLK	+2y9	804.41	1130.62	89.8
1246	RS1	O15537	FQDSSQWLQIDLK	+2y8	804.41	1043.59	89.8
1247	RS1	O15537	FQDSSQWLQIDLK	+2y7	804.41	915.53	89.8
1248	RS1	O15537	FQDSSQWLQIDLK	+2y6	804.41	729.45	89.8
1249	APOC3	P02656	DALSSVQESQVAQQAR	+2y10	858.93	1144.57	93.7
1250	APOC3	P02656	DALSSVQESQVAQQAR	+2y8	858.93	887.47	93.7
1251	APOC3	P02656	DALSSVQESQVAQQAR	+2y5	858.93	573.31	93.7
1252	APOC3	P02656	DALSSVQESQVAQQAR	+2y4	858.93	502.27	93.7
1253	APOC3	P02656	DALSSVQESQVAQQAR	+2y3	858.93	374.21	93.7
1254	APOC3	P02656	GWVTDGFSSLK	+2y9	598.80	953.49	74.8
1255	APOC3	P02656	GWVTDGFSSLK	+2y8	598.80	854.43	74.8
1256	APOC3	P02656	GWVTDGFSSLK	+2y7	598.80	753.38	74.8
1257	APOC3	P02656	GWVTDGFSSLK	+2y6	598.80	638.35	74.8
1258	APOC3	P02656	GWVTDGFSSLK	+2y4	598.80	434.26	74.8
1259	APOC3	P02656	DYWSTVK	+2y6	449.72	783.40	63.9
1260	APOC3	P02656	DYWSTVK	+2y5	449.72	620.34	63.9
1261	APOC3	P02656	DYWSTVK	+2y4	449.72	434.26	63.9
1262	APOC3	P02656	DYWSTVK	+2y3	449.72	347.23	63.9
1263	APOC3	P02656	FSEFWDLDPEVRPTSAVAA	+2y13	1069.02	1325.71	109
1264	APOC3	P02656	FSEFWDLDPEVRPTSAVAA	+2y12	1069.02	1212.62	109
1265	APOC3	P02656	FSEFWDLDPEVRPTSAVAA	+2y11	1069.02	1097.59	109
1266	APOC3	P02656	FSEFWDLDPEVRPTSAVAA	+2y9	1069.02	871.50	109
1267	APOC3	P02656	FSEFWDLDPEVRPTSAVAA	+2y8	1069.02	772.43	109
1268	APOE	P02649	WELALGR	+2y6	422.74	658.39	61.9
1269	APOE	P02649	WELALGR	+2y5	422.74	529.35	61.9
1270	APOE	P02649	WELALGR	+2y4	422.74	416.26	61.9
1271	APOE	P02649	WELALGR	+2y3	422.74	345.22	61.9
1272	APOE	P02649	SELEEQLTPVAEETR	+2y10	865.93	1143.60	94.2
1273	APOE	P02649	SELEEQLTPVAEETR	+2y9	865.93	1015.54	94.2
1274	APOE	P02649	SELEEQLTPVAEETR	+2y8	865.93	902.46	94.2
1275	APOE	P02649	SELEEQLTPVAEETR	+2y7	865.93	801.41	94.2
1276	APOE	P02649	SELEEQLTPVAEETR	+2y5	865.93	605.29	94.2
1277	APOE	P02649	ELQAAQAR	+2y6	443.74	644.35	63.5
1278	APOE	P02649	ELQAAQAR	+2y5	443.74	516.29	63.5
1279	APOE	P02649	ELQAAQAR	+2y4	443.74	445.25	63.5
1280	APOE	P02649	ELQAAQAR	+2y3	443.74	374.21	63.5
1281	APOE	P02649	AATVGSLAGQPLQER	+2y11	749.40	1155.61	85.7
1282	APOE	P02649	AATVGSLAGQPLQER	+2y10	749.40	1098.59	85.7
1283	APOE	P02649	AATVGSLAGQPLQER	+2y8	749.40	898.47	85.7
1284	APOE	P02649	AATVGSLAGQPLQER	+2y7	749.40	827.44	85.7
1285	APOE	P02649	AATVGSLAGQPLQER	+2y5	749.40	642.36	85.7
1286	APOE	P02649	LEEQAQQIR	+2y7	557.80	872.46	71.8
1287	APOE	P02649	LEEQAQQIR	+2y6	557.80	743.42	71.8

1288	APOE	P02649	LEEQAQQIR	+2y5	557.80	615.36	71.8
1289	APOE	P02649	LEEQAQQIR	+2y4	557.80	544.32	71.8
1290	APOE	P02649	LEEQAQQIR	+2y3	557.80	416.26	71.8
1291	CAT	P04040	LNVITVGPR	+2y7	484.80	741.46	66.5
1292	CAT	P04040	LNVITVGPR	+2y6	484.80	642.39	66.5
1293	CAT	P04040	LNVITVGPR	+2y5	484.80	529.31	66.5
1294	CAT	P04040	LNVITVGPR	+2y4	484.80	428.26	66.5
1295	CAT	P04040	LNVITVGPR	+2y3	484.80	329.19	66.5
1296	CAT	P04040	FSTVAGESGSADTVR	+2y11	742.35	1049.49	85.2
1297	CAT	P04040	FSTVAGESGSADTVR	+2y10	742.35	978.45	85.2
1298	CAT	P04040	FSTVAGESGSADTVR	+2y8	742.35	792.38	85.2
1299	CAT	P04040	FSTVAGESGSADTVR	+2y7	742.35	705.35	85.2
1300	CAT	P04040	FSTVAGESGSADTVR	+2y3	742.35	375.24	85.2
1301	CAT	P04040	FNTANDDNVTQVR	+2y9	747.35	1060.50	85.6
1302	CAT	P04040	FNTANDDNVTQVR	+2y7	747.35	831.43	85.6
1303	CAT	P04040	FNTANDDNVTQVR	+2y6	747.35	716.40	85.6
1304	CAT	P04040	FNTANDDNVTQVR	+2y4	747.35	503.29	85.6
1305	CAT	P04040	FNTANDDNVTQVR	+2y3	747.35	402.25	85.6
1306	CAT	P04040	AFYVNVLNEEQR	+2y8	741.37	1001.50	85.2
1307	CAT	P04040	AFYVNVLNEEQR	+2y7	741.37	887.46	85.2
1308	CAT	P04040	AFYVNVLNEEQR	+2y6	741.37	788.39	85.2
1309	CAT	P04040	AFYVNVLNEEQR	+2y5	741.37	675.31	85.2
1310	CAT	P04040	AFYVNVLNEEQR	+2y2	741.37	303.18	85.2
1311	CAT	P04040	DAQIFIQK	+2y6	481.77	776.47	66.2
1312	CAT	P04040	DAQIFIQK	+2y5	481.77	648.41	66.2
1313	CAT	P04040	DAQIFIQK	+2y4	481.77	535.32	66.2
1314	CAT	P04040	DAQIFIQK	+2y3	481.77	388.26	66.2
1315	VCAN	P13611	LATVGELQAAWR	+2y8	657.86	930.48	79.1
1316	VCAN	P13611	LATVGELQAAWR	+2y5	657.86	631.33	79.1
1317	VCAN	P13611	LATVGELQAAWR	+2y4	657.86	503.27	79.1
1318	VCAN	P13611	LATVGELQAAWR	+2y3	657.86	432.24	79.1
1319	VCAN	P13611	LATVGELQAAWR	+2y2	657.86	361.20	79.1
1320	VCAN	P13611	ITEGTTQEEFPWK	+2y10	783.38	1222.57	88.2
1321	VCAN	P13611	ITEGTTQEEFPWK	+2y8	783.38	1064.50	88.2
1322	VCAN	P13611	ITEGTTQEEFPWK	+2y6	783.38	835.40	88.2
1323	VCAN	P13611	ITEGTTQEEFPWK	+2y4	783.38	577.31	88.2
1324	VCAN	P13611	ITEGTTQEEFPWK	+2y3	783.38	430.24	88.2
1325	VCAN	P13611	DPEAAEAR	+2y6	429.70	646.32	62.4
1326	VCAN	P13611	DPEAAEAR	+2y5	429.70	517.27	62.4
1327	VCAN	P13611	DPEAAEAR	+2y4	429.70	446.24	62.4
1328	VCAN	P13611	DPEAAEAR	+2y3	429.70	375.20	62.4
1329	VCAN	P13611	QEIESETTSEEQIQEEK	+2y11	1018.96	1321.61	105.4
1330	VCAN	P13611	QEIESETTSEEQIQEEK	+2y10	1018.96	1220.56	105.4
1331	VCAN	P13611	QEIESETTSEEQIQEEK	+2y9	1018.96	1119.52	105.4
1332	VCAN	P13611	QEIESETTSEEQIQEEK	+2y7	1018.96	903.44	105.4
1333	VCAN	P13611	QEIESETTSEEQIQEEK	+2y4	1018.96	533.26	105.4
1334	VCAN	P13611	GQDSTIAASEQQVAAR	+2y10	816.40	1030.53	90.6
1335	VCAN	P13611	GQDSTIAASEQQVAAR	+2y9	816.40	959.49	90.6
1336	VCAN	P13611	GQDSTIAASEQQVAAR	+2y8	816.40	888.45	90.6
1337	VCAN	P13611	GQDSTIAASEQQVAAR	+2y7	816.40	801.42	90.6

1338	VCAN	P13611	GQDSTIAASEQQVAAR	+2y6	816.40	672.38	90.6
1339	C5	P01031	FQNSAILTIQPK	+2y10	680.39	1084.64	80.7
1340	C5	P01031	FQNSAILTIQPK	+2y7	680.39	812.52	80.7
1341	C5	P01031	FQNSAILTIQPK	+2y6	680.39	699.44	80.7
1342	C5	P01031	FQNSAILTIQPK	+2y5	680.39	586.36	80.7
1343	C5	P01031	FQNSAILTIQPK	+2y3	680.39	372.22	80.7
1344	C5	P01031	VVTEADVYITFGIR	+2y7	791.93	869.49	88.8
1345	C5	P01031	VVTEADVYITFGIR	+2y6	791.93	706.42	88.8
1346	C5	P01031	VVTEADVYITFGIR	+2y5	791.93	593.34	88.8
1347	C5	P01031	VVTEADVYITFGIR	+2y4	791.93	492.29	88.8
1348	C5	P01031	VVTEADVYITFGIR	+2y3	791.93	345.22	88.8
1349	C5	P01031	TDAPDLPEENQAR	+2y10	728.34	1168.56	84.2
1350	C5	P01031	TDAPDLPEENQAR	+2y9	728.34	1071.51	84.2
1351	C5	P01031	TDAPDLPEENQAR	+2y8	728.34	956.48	84.2
1352	C5	P01031	TDAPDLPEENQAR	+2y7	728.34	843.40	84.2
1353	C5	P01031	TDAPDLPEENQAR	+2y5	728.34	617.30	84.2
1354	C5	P01031	ENSLYLTAFTVIGIR	+2y11	848.97	1253.73	93
1355	C5	P01031	ENSLYLTAFTVIGIR	+2y10	848.97	1090.66	93
1356	C5	P01031	ENSLYLTAFTVIGIR	+2y9	848.97	977.58	93
1357	C5	P01031	ENSLYLTAFTVIGIR	+2y8	848.97	876.53	93
1358	C5	P01031	ENSLYLTAFTVIGIR	+2y7	848.97	805.49	93
1359	C5	P01031	ALVEGVDQLFTDYQIK	+2y10	919.98	1270.63	98.2
1360	C5	P01031	ALVEGVDQLFTDYQIK	+2y8	919.98	1027.55	98.2
1361	C5	P01031	ALVEGVDQLFTDYQIK	+2y6	919.98	767.39	98.2
1362	C5	P01031	ALVEGVDQLFTDYQIK	+2y5	919.98	666.35	98.2
1363	C5	P01031	ALVEGVDQLFTDYQIK	+2y4	919.98	551.32	98.2
1364	GAPDH	P04406	LVINGNPITIFQER	+2y10	807.45	1174.62	90
1365	GAPDH	P04406	LVINGNPITIFQER	+2y8	807.45	1003.56	90
1366	GAPDH	P04406	LVINGNPITIFQER	+2y6	807.45	793.42	90
1367	GAPDH	P04406	LVINGNPITIFQER	+2y5	807.45	692.37	90
1368	GAPDH	P04406	LVINGNPITIFQER	+2y4	807.45	579.29	90
1369	GAPDH	P04406	GALQNIIPASTGAAK	+2y11	706.40	1042.59	82.6
1370	GAPDH	P04406	GALQNIIPASTGAAK	+2y10	706.40	928.55	82.6
1371	GAPDH	P04406	GALQNIIPASTGAAK	+2y9	706.40	815.46	82.6
1372	GAPDH	P04406	GALQNIIPASTGAAK	+2y8	706.40	702.38	82.6
1373	GAPDH	P04406	GALQNIIPASTGAAK	+2y6	706.40	534.29	82.6
1374	GAPDH	P04406	VIPELNGK	+2y6	435.26	657.36	62.8
1375	GAPDH	P04406	VIPELNGK	+2y5	435.26	560.30	62.8
1376	GAPDH	P04406	VIPELNGK	+2y3	435.26	318.18	62.8
1377	GAPDH	P04406	LISWYDNEFGYSNR	+2y10	882.40	1264.52	95.4
1378	GAPDH	P04406	LISWYDNEFGYSNR	+2y9	882.40	1101.46	95.4
1379	GAPDH	P04406	LISWYDNEFGYSNR	+2y8	882.40	986.43	95.4
1380	GAPDH	P04406	LISWYDNEFGYSNR	+2y6	882.40	743.35	95.4
1381	GAPDH	P04406	LISWYDNEFGYSNR	+2y5	882.40	596.28	95.4
1382	KRT1	P04264	FLEQQNQVLQTK	+2y8	738.40	958.53	84.9
1383	KRT1	P04264	FLEQQNQVLQTK	+2y7	738.40	830.47	84.9
1384	KRT1	P04264	FLEQQNQVLQTK	+2y5	738.40	588.37	84.9
1385	KRT1	P04264	FLEQQNQVLQTK	+2y4	738.40	489.30	84.9
1386	KRT1	P04264	FLEQQNQVLQTK	+2y3	738.40	376.22	84.9
1387	KRT1	P04264	AQYEDIAQK	+2y7	533.26	866.43	70

1388	KRT1	P04264	AQYEDIAQK	+2y6	533.26	703.36	70
1389	KRT1	P04264	AQYEDIAQK	+2y5	533.26	574.32	70
1390	KRT1	P04264	AQYEDIAQK	+2y4	533.26	459.29	70
1391	KRT1	P04264	AQYEDIAQK	+2y3	533.26	346.21	70
1392	KRT1	P04264	QISNLQQSISDAEQR	+2y9	858.93	1033.49	93.7
1393	KRT1	P04264	QISNLQQSISDAEQR	+2y8	858.93	905.43	93.7
1394	KRT1	P04264	QISNLQQSISDAEQR	+2y6	858.93	705.32	93.7
1395	KRT1	P04264	QISNLQQSISDAEQR	+2y4	858.93	503.26	93.7
1396	KRT1	P04264	QISNLQQSISDAEQR	+2y2	858.93	303.18	93.7
1397	KRT1	P04264	LALDLEIATYR	+2y8	639.36	980.50	77.7
1398	KRT1	P04264	LALDLEIATYR	+2y6	639.36	752.39	77.7
1399	KRT1	P04264	LALDLEIATYR	+2y5	639.36	623.35	77.7
1400	KRT1	P04264	LALDLEIATYR	+2y4	639.36	510.27	77.7
1401	KRT1	P04264	LALDLEIATYR	+2y3	639.36	439.23	77.7
1402	KRT1	P04264	GSGGGSSGGSIGGR	+2y10	546.76	834.41	71
1403	KRT1	P04264	GSGGGSSGGSIGGR	+2y9	546.76	777.38	71
1404	KRT1	P04264	GSGGGSSGGSIGGR	+2y8	546.76	690.35	71
1405	KRT1	P04264	GSGGGSSGGSIGGR	+2y7	546.76	603.32	71
1406	KRT1	P04264	GSGGGSSGGSIGGR	+2y6	546.76	546.30	71
1407	HPR	P00739	TEGDGVYTLNDK	+2y10	656.31	1081.52	79
1408	HPR	P00739	TEGDGVYTLNDK	+2y6	656.31	753.38	79
1409	HPR	P00739	TEGDGVYTLNDK	+2y5	656.31	590.31	79
1410	HPR	P00739	TEGDGVYTLNDK	+2y4	656.31	489.27	79
1411	HPR	P00739	TEGDGVYTLNDK	+2y3	656.31	376.18	79
1412	HPR	P00739	NPANPVQR	+2y6	448.24	684.38	63.8
1413	HPR	P00739	NPANPVQR	+2y5	448.24	613.34	63.8
1414	HPR	P00739	NPANPVQR	+2y4	448.24	499.30	63.8
1415	HPR	P00739	NPANPVQR	+2y3	448.24	402.25	63.8
1416	HPR	P00739	NPANPVQR	+2y2	448.24	303.18	63.8
1417	HPR	P00739	GSFPWQAK	+2y6	460.73	776.41	64.7
1418	HPR	P00739	GSFPWQAK	+2y5	460.73	629.34	64.7
1419	HPR	P00739	GSFPWQAK	+2y4	460.73	532.29	64.7
1420	HPR	P00739	GSFPWQAK	+2y3	460.73	346.21	64.7
1421	HPR	P00739	DIAPTLTLYVGK	+2y9	645.87	991.58	78.2
1422	HPR	P00739	DIAPTLTLYVGK	+2y8	645.87	894.53	78.2
1423	HPR	P00739	DIAPTLTLYVGK	+2y6	645.87	680.40	78.2
1424	HPR	P00739	DIAPTLTLYVGK	+2y5	645.87	579.35	78.2
1425	HPR	P00739	DIAPTLTLYVGK	+2y4	645.87	466.27	78.2
1426	HPR	P00739	QLVEIEK	+2y5	429.75	617.35	62.4
1427	HPR	P00739	QLVEIEK	+2y4	429.75	518.28	62.4
1428	HPR	P00739	QLVEIEK	+2y3	429.75	389.24	62.4
1429	HBB	P68871	LLVVYPWTQR	+2y8	637.87	1048.56	77.6
1430	HBB	P68871	LLVVYPWTQR	+2y7	637.87	949.49	77.6
1431	HBB	P68871	LLVVYPWTQR	+2y6	637.87	850.42	77.6
1432	HBB	P68871	LLVVYPWTQR	+2y5	637.87	687.36	77.6
1433	HBB	P68871	LLVVYPWTQR	+2y3	637.87	404.23	77.6
1434	HBB	P68871	EFTPPVQAAYQK	+2y9	689.85	1001.54	81.4
1435	HBB	P68871	EFTPPVQAAYQK	+2y8	689.85	904.49	81.4
1436	HBB	P68871	EFTPPVQAAYQK	+2y6	689.85	708.37	81.4
1437	HBB	P68871	EFTPPVQAAYQK	+2y5	689.85	580.31	81.4

1438	HBB	P68871	EFTPPVQAAYQK	+2y3	689.85	438.23	81.4
1439	HRG	P04196	DGYLFQLLR	+2y7	562.81	952.56	72.1
1440	HRG	P04196	DGYLFQLLR	+2y6	562.81	789.50	72.1
1441	HRG	P04196	DGYLFQLLR	+2y5	562.81	676.41	72.1
1442	HRG	P04196	DGYLFQLLR	+2y4	562.81	529.35	72.1
1443	HRG	P04196	DGYLFQLLR	+2y3	562.81	401.29	72.1
1444	HRG	P04196	DSPVLIDFFEDTER	+2y9	841.90	1171.53	92.5
1445	HRG	P04196	DSPVLIDFFEDTER	+2y8	841.90	1058.44	92.5
1446	HRG	P04196	DSPVLIDFFEDTER	+2y7	841.90	943.42	92.5
1447	HRG	P04196	DSPVLIDFFEDTER	+2y6	841.90	796.35	92.5
1448	HRG	P04196	DSPVLIDFFEDTER	+2y3	841.90	405.21	92.5
1449	HRG	P04196	EENDDFASFR	+2y6	615.26	742.35	76
1450	HRG	P04196	EENDDFASFR	+2y5	615.26	627.32	76
1451	HRG	P04196	EENDDFASFR	+2y4	615.26	480.26	76
1452	HRG	P04196	EENDDFASFR	+2y3	615.26	409.22	76
1453	HRG	P04196	EENDDFASFR	+2y2	615.26	322.19	76
1454	HRG	P04196	GGEGTGYFVDFSVR	+2y9	745.85	1089.54	85.5
1455	HRG	P04196	GGEGTGYFVDFSVR	+2y7	745.85	869.45	85.5
1456	HRG	P04196	GGEGTGYFVDFSVR	+2y6	745.85	722.38	85.5
1457	HRG	P04196	GGEGTGYFVDFSVR	+2y5	745.85	623.31	85.5
1458	HRG	P04196	GGEGTGYFVDFSVR	+2y4	745.85	508.29	85.5
1459	HRG	P04196	ADLFYDVEALDLESPK	+2y12	912.95	1378.67	97.7
1460	HRG	P04196	ADLFYDVEALDLESPK	+2y11	912.95	1215.61	97.7
1461	HRG	P04196	ADLFYDVEALDLESPK	+2y9	912.95	1001.51	97.7
1462	HRG	P04196	ADLFYDVEALDLESPK	+2y8	912.95	872.47	97.7
1463	HRG	P04196	ADLFYDVEALDLESPK	+2y3	912.95	331.20	97.7
1464	CD14	P08571	VDADADPR	+2y7	429.70	759.33	62.4
1465	CD14	P08571	VDADADPR	+2y6	429.70	644.30	62.4
1466	CD14	P08571	VDADADPR	+2y5	429.70	573.26	62.4
1467	CD14	P08571	VDADADPR	+2y4	429.70	458.24	62.4
1468	CD14	P08571	VDADADPR	+2y3	429.70	387.20	62.4
1469	CD14	P08571	SWLAELQQWLKPGLK	+2y10	899.01	1210.73	96.7
1470	CD14	P08571	SWLAELQQWLKPGLK	+2y9	899.01	1097.65	96.7
1471	CD14	P08571	SWLAELQQWLKPGLK	+2y7	899.01	841.53	96.7
1472	CD14	P08571	SWLAELQQWLKPGLK	+2y5	899.01	542.37	96.7
1473	CD14	P08571	SWLAELQQWLKPGLK	+2y4	899.01	414.27	96.7
1474	CD14	P08571	AFPALTSLDLSDNPGLGER	+2y11	987.00	1172.55	103.1
1475	CD14	P08571	AFPALTSLDLSDNPGLGER	+2y9	987.00	944.44	103.1
1476	CD14	P08571	AFPALTSLDLSDNPGLGER	+2y8	987.00	857.41	103.1
1477	CD14	P08571	AFPALTSLDLSDNPGLGER	+2y7	987.00	742.38	103.1
1478	CD14	P08571	AFPALTSLDLSDNPGLGER	+2y6	987.00	628.34	103.1
1479	CD14	P08571	FPAIQNLALR	+2y8	571.84	898.55	72.8
1480	CD14	P08571	FPAIQNLALR	+2y7	571.84	827.51	72.8
1481	CD14	P08571	FPAIQNLALR	+2y6	571.84	714.43	72.8
1482	CD14	P08571	FPAIQNLALR	+2y5	571.84	586.37	72.8
1483	CD14	P08571	FPAIQNLALR	+2y3	571.84	359.24	72.8
1484	CD14	P08571	ATVNPSAPR	+2y7	456.75	740.40	64.4
1485	CD14	P08571	ATVNPSAPR	+2y6	456.75	641.34	64.4
1486	CD14	P08571	ATVNPSAPR	+2y5	456.75	527.29	64.4
1487	CD14	P08571	ATVNPSAPR	+2y4	456.75	430.24	64.4

1488	CD14	P08571	ATVNPSAPR	+2y3	456.75	343.21	64.4
1489	RNASE1	P07998	LTNGSR	+2y5	324.18	534.26	54.7
1490	RNASE1	P07998	LTNGSR	+2y4	324.18	433.22	54.7
1491	RNASE1	P07998	LTNGSR	+2y3	324.18	319.17	54.7
1492	SOD3	P08294	VTGVVLFR	+2y7	445.78	791.48	63.6
1493	SOD3	P08294	VTGVVLFR	+2y6	445.78	690.43	63.6
1494	SOD3	P08294	VTGVVLFR	+2y4	445.78	534.34	63.6
1495	SOD3	P08294	VTGVVLFR	+2y3	445.78	435.27	63.6
1496	SOD3	P08294	GGNQASVENGNAGR	+2y10	665.81	974.46	79.7
1497	SOD3	P08294	GGNQASVENGNAGR	+2y9	665.81	903.43	79.7
1498	SOD3	P08294	GGNQASVENGNAGR	+2y8	665.81	816.40	79.7
1499	SOD3	P08294	GGNQASVENGNAGR	+2y7	665.81	717.33	79.7
1500	SOD3	P08294	GGNQASVENGNAGR	+2y6	665.81	588.28	79.7
1501	VTN	P04004	AVRPGYPK	+2y7	444.26	816.47	63.5
1502	VTN	P04004	AVRPGYPK	+2y6	444.26	717.40	63.5
1503	VTN	P04004	AVRPGYPK	+2y5	444.26	561.30	63.5
1504	VTN	P04004	AVRPGYPK	+2y4	444.26	464.25	63.5
1505	VTN	P04004	AVRPGYPK	+2y3	444.26	407.23	63.5
1506	VTN	P04004	DVWGIEGPIDAAFTR	+2y12	823.91	1246.64	91.2
1507	VTN	P04004	DVWGIEGPIDAAFTR	+2y10	823.91	1076.54	91.2
1508	VTN	P04004	DVWGIEGPIDAAFTR	+2y9	823.91	947.49	91.2
1509	VTN	P04004	DVWGIEGPIDAAFTR	+2y8	823.91	890.47	91.2
1510	VTN	P04004	DVWGIEGPIDAAFTR	+2y5	823.91	565.31	91.2
1511	VTN	P04004	FEDGVLDPDYPR	+2y10	711.83	1146.54	83
1512	VTN	P04004	FEDGVLDPDYPR	+2y7	711.83	875.43	83
1513	VTN	P04004	FEDGVLDPDYPR	+2y6	711.83	762.34	83
1514	VTN	P04004	FEDGVLDPDYPR	+2y5	711.83	647.31	83
1515	VTN	P04004	FEDGVLDPDYPR	+2y3	711.83	435.24	83
1516	VTN	P04004	DSWEDIFELLFWGR	+2y8	906.93	1067.57	97.2
1517	VTN	P04004	DSWEDIFELLFWGR	+2y6	906.93	791.46	97.2
1518	VTN	P04004	DSWEDIFELLFWGR	+2y5	906.93	678.37	97.2
1519	VTN	P04004	DSWEDIFELLFWGR	+2y4	906.93	565.29	97.2
1520	VTN	P04004	DSWEDIFELLFWGR	+2y3	906.93	418.22	97.2
1521	VTN	P04004	VDTVDPPYPR	+2y8	579.79	944.48	73.4
1522	VTN	P04004	VDTVDPPYPR	+2y7	579.79	843.44	73.4
1523	VTN	P04004	VDTVDPPYPR	+2y6	579.79	744.37	73.4
1524	VTN	P04004	VDTVDPPYPR	+2y5	579.79	629.34	73.4
1525	VTN	P04004	VDTVDPPYPR	+2y4	579.79	532.29	73.4
1526	CFI	G3XAM2	AQLGDLPWQVAIK	+2y11	719.91	1239.71	83.6
1527	CFI	G3XAM2	AQLGDLPWQVAIK	+2y10	719.91	1126.63	83.6
1528	CFI	G3XAM2	AQLGDLPWQVAIK	+2y8	719.91	954.58	83.6
1529	CFI	G3XAM2	AQLGDLPWQVAIK	+2y7	719.91	841.49	83.6
1530	CFI	G3XAM2	AQLGDLPWQVAIK	+2y3	719.91	331.23	83.6
1531	CFI	G3XAM2	IVIEYVDR	+2y7	503.78	893.47	67.8
1532	CFI	G3XAM2	IVIEYVDR	+2y6	503.78	794.40	67.8
1533	CFI	G3XAM2	IVIEYVDR	+2y5	503.78	681.32	67.8
1534	CFI	G3XAM2	IVIEYVDR	+2y4	503.78	552.28	67.8
1535	CFI	G3XAM2	IVIEYVDR	+2y3	503.78	389.21	67.8
1536	CFI	G3XAM2	VFSLQWGEVK	+2y8	596.82	946.50	74.6
1537	CFI	G3XAM2	VFSLQWGEVK	+2y7	596.82	859.47	74.6

1538	CFI	G3XAM2	VFSLQWGEVK	+2y6	596.82	746.38	74.6
1539	CFI	G3XAM2	VFSLQWGEVK	+2y5	596.82	618.32	74.6
1540	CFI	G3XAM2	VFSLQWGEVK	+2y4	596.82	432.25	74.6
1541	C6	P13671	VPANLENVGFEVQTAEDDLK	+2y12	1094.54	1351.64	110.9
1542	C6	P13671	VPANLENVGFEVQTAEDDLK	+2y11	1094.54	1294.62	110.9
1543	C6	P13671	VPANLENVGFEVQTAEDDLK	+2y10	1094.54	1147.55	110.9
1544	C6	P13671	VPANLENVGFEVQTAEDDLK	+2y9	1094.54	1018.51	110.9
1545	C6	P13671	VPANLENVGFEVQTAEDDLK	+2y7	1094.54	791.38	110.9
1546	C6	P13671	SEYGAALAWEK	+2y9	612.80	1008.51	75.8
1547	C6	P13671	SEYGAALAWEK	+2y8	612.80	845.45	75.8
1548	C6	P13671	SEYGAALAWEK	+2y6	612.80	717.39	75.8
1549	C6	P13671	SEYGAALAWEK	+2y5	612.80	646.36	75.8
1550	C6	P13671	SEYGAALAWEK	+2y3	612.80	462.23	75.8
1551	C6	P13671	TFSEWLESVK	+2y8	613.31	977.49	75.8
1552	C6	P13671	TFSEWLESVK	+2y7	613.31	890.46	75.8
1553	C6	P13671	TFSEWLESVK	+2y6	613.31	761.42	75.8
1554	C6	P13671	TFSEWLESVK	+2y5	613.31	575.34	75.8
1555	C6	P13671	TFSEWLESVK	+2y4	613.31	462.26	75.8
1556	C6	P13671	ENPAVIDFELAPIVDLVR	+2y12	1005.55	1386.76	104.4
1557	C6	P13671	ENPAVIDFELAPIVDLVR	+2y10	1005.55	1124.67	104.4
1558	C6	P13671	ENPAVIDFELAPIVDLVR	+2y9	1005.55	995.62	104.4
1559	C6	P13671	ENPAVIDFELAPIVDLVR	+2y8	1005.55	882.54	104.4
1560	C6	P13671	ENPAVIDFELAPIVDLVR	+2y7	1005.55	811.50	104.4
1561	C6	P13671	QLEWGLER	+2y6	515.77	789.39	68.7
1562	C6	P13671	QLEWGLER	+2y5	515.77	660.35	68.7
1563	C6	P13671	QLEWGLER	+2y4	515.77	474.27	68.7
1564	C6	P13671	QLEWGLER	+2y3	515.77	417.25	68.7
1565	C6	P13671	QLEWGLER	+2y2	515.77	304.16	68.7
1566	C7	P10643	LSGNVLSYTFQVK	+2y8	728.40	985.54	84.2
1567	C7	P10643	LSGNVLSYTFQVK	+2y7	728.40	872.45	84.2
1568	C7	P10643	LSGNVLSYTFQVK	+2y6	728.40	785.42	84.2
1569	C7	P10643	LSGNVLSYTFQVK	+2y5	728.40	622.36	84.2
1570	C7	P10643	LSGNVLSYTFQVK	+2y3	728.40	374.24	84.2
1571	C7	P10643	INNDFNYEFYNSTWSYVK	+2y10	1152.52	1294.61	115.1
1572	C7	P10643	INNDFNYEFYNSTWSYVK	+2y7	1152.52	870.44	115.1
1573	C7	P10643	INNDFNYEFYNSTWSYVK	+2y6	1152.52	783.40	115.1
1574	C7	P10643	INNDFNYEFYNSTWSYVK	+2y5	1152.52	682.36	115.1
1575	C7	P10643	INNDFNYEFYNSTWSYVK	+2y4	1152.52	496.28	115.1
1576	C7	P10643	AASGTQNNVLR	+2y9	565.80	988.52	72.4
1577	C7	P10643	AASGTQNNVLR	+2y8	565.80	901.49	72.4
1578	C7	P10643	AASGTQNNVLR	+2y7	565.80	844.46	72.4
1579	C7	P10643	AASGTQNNVLR	+2y6	565.80	743.42	72.4
1580	C7	P10643	AASGTQNNVLR	+2y5	565.80	615.36	72.4
1581	C7	P10643	YSAWAESVTNLPQVIK	+2y12	903.48	1298.73	97
1582	C7	P10643	YSAWAESVTNLPQVIK	+2y10	903.48	1098.65	97
1583	C7	P10643	YSAWAESVTNLPQVIK	+2y8	903.48	912.55	97
1584	C7	P10643	YSAWAESVTNLPQVIK	+2y7	903.48	811.50	97
1585	C7	P10643	YSAWAESVTNLPQVIK	+2y5	903.48	584.38	97
1586	C7	P10643	LTPLYELVK	+2y7	538.32	861.51	70.4
1587	C7	P10643	LTPLYELVK	+2y6	538.32	764.46	70.4

1588	C7	P10643	LTPLYELVK	+2y5	538.32	651.37	70.4
1589	C7	P10643	LTPLYELVK	+2y4	538.32	488.31	70.4
1590	C7	P10643	LTPLYELVK	+2y3	538.32	359.27	70.4
1591	CA2	P00918	GGPLDGTYR	+2y7	468.23	821.42	65.3
1592	CA2	P00918	GGPLDGTYR	+2y6	468.23	724.36	65.3
1593	CA2	P00918	GGPLDGTYR	+2y5	468.23	611.28	65.3
1594	CA2	P00918	GGPLDGTYR	+2y4	468.23	496.25	65.3
1595	CA2	P00918	GGPLDGTYR	+2y2	468.23	338.18	65.3
1596	CA2	P00918	AVQQPDGLAVLGIFLK	+2y12	834.99	1242.75	92
1597	CA2	P00918	AVQQPDGLAVLGIFLK	+2y8	834.99	860.56	92
1598	CA2	P00918	AVQQPDGLAVLGIFLK	+2y6	834.99	690.45	92
1599	CA2	P00918	AVQQPDGLAVLGIFLK	+2y5	834.99	577.37	92
1600	CA2	P00918	AVQQPDGLAVLGIFLK	+2y3	834.99	407.27	92
1601	CA2	P00918	VGSAKPGLQK	+2y9	492.80	885.52	67
1602	CA2	P00918	VGSAKPGLQK	+2y8	492.80	828.49	67
1603	CA2	P00918	VGSAKPGLQK	+2y7	492.80	741.46	67
1604	CA2	P00918	VGSAKPGLQK	+2y6	492.80	670.42	67
1605	CA2	P00918	VGSAKPGLQK	+2y5	492.80	542.33	67
1606	CA2	P00918	VVDVLDSIK	+2y7	494.29	789.44	67.2
1607	CA2	P00918	VVDVLDSIK	+2y6	494.29	674.41	67.2
1608	CA2	P00918	VVDVLDSIK	+2y5	494.29	575.34	67.2
1609	CA2	P00918	VVDVLDSIK	+2y4	494.29	462.26	67.2
1610	CA2	P00918	VVDVLDSIK	+2y3	494.29	347.23	67.2
1611	CA2	P00918	SADFTNFDPR	+2y8	585.26	1011.45	73.8
1612	CA2	P00918	SADFTNFDPR	+2y7	585.26	896.43	73.8
1613	CA2	P00918	SADFTNFDPR	+2y6	585.26	749.36	73.8
1614	CA2	P00918	SADFTNFDPR	+2y5	585.26	648.31	73.8
1615	CA2	P00918	SADFTNFDPR	+2y3	585.26	387.20	73.8
1616	LRP2	E9PC35	VFWTDTVQNK	+2y8	619.31	991.48	76.3
1617	LRP2	E9PC35	VFWTDTVQNK	+2y7	619.31	805.41	76.3
1618	LRP2	E9PC35	VFWTDTVQNK	+2y6	619.31	704.36	76.3
1619	LRP2	E9PC35	VFWTDTVQNK	+2y5	619.31	589.33	76.3
1620	LRP2	E9PC35	VFWTDTVQNK	+2y3	619.31	389.21	76.3
1621	LRP2	E9PC35	TDNDGLGFR	+2y7	497.73	778.38	67.4
1622	LRP2	E9PC35	TDNDGLGFR	+2y6	497.73	664.34	67.4
1623	LRP2	E9PC35	TDNDGLGFR	+2y5	497.73	549.31	67.4
1624	LRP2	E9PC35	TDNDGLGFR	+2y3	497.73	379.21	67.4
1625	LRP2	E9PC35	TDNDGLGFR	+2y2	497.73	322.19	67.4
1626	LRP2	E9PC35	VENVESLAFDWISK	+2y10	818.91	1195.60	90.8
1627	LRP2	E9PC35	VENVESLAFDWISK	+2y9	818.91	1066.56	90.8
1628	LRP2	E9PC35	VENVESLAFDWISK	+2y7	818.91	866.44	90.8
1629	LRP2	E9PC35	VENVESLAFDWISK	+2y6	818.91	795.40	90.8
1630	LRP2	E9PC35	VENVESLAFDWISK	+2y5	818.91	648.34	90.8
1631	LRP2	E9PC35	TVVQYLNNPR	+2y8	602.33	1003.53	75
1632	LRP2	E9PC35	TVVQYLNNPR	+2y7	602.33	904.46	75
1633	LRP2	E9PC35	TVVQYLNNPR	+2y6	602.33	776.40	75
1634	LRP2	E9PC35	TVVQYLNNPR	+2y4	602.33	500.26	75
1635	LRP2	E9PC35	TVVQYLNNPR	+2y3	602.33	386.21	75
1636	LRP2	E9PC35	LYWVDAYFDK	+2y8	660.32	1043.48	79.3
1637	LRP2	E9PC35	LYWVDAYFDK	+2y7	660.32	857.40	79.3

1638	LRP2	E9PC35	LYWVDAYFDK	+2y6	660.32	758.34	79.3
1639	LRP2	E9PC35	LYWVDAYFDK	+2y5	660.32	643.31	79.3
1640	LRP2	E9PC35	LYWVDAYFDK	+2y4	660.32	572.27	79.3
1641	KRT2	P35908	FLEQQNQVLQTK	+2y8	738.40	958.53	84.9
1642	KRT2	P35908	FLEQQNQVLQTK	+2y7	738.40	830.47	84.9
1643	KRT2	P35908	FLEQQNQVLQTK	+2y5	738.40	588.37	84.9
1644	KRT2	P35908	FLEQQNQVLQTK	+2y4	738.40	489.30	84.9
1645	KRT2	P35908	FLEQQNQVLQTK	+2y3	738.40	376.22	84.9
1646	KRT2	P35908	LALDVEIATYR	+2y8	632.35	966.49	77.2
1647	KRT2	P35908	LALDVEIATYR	+2y7	632.35	851.46	77.2
1648	KRT2	P35908	LALDVEIATYR	+2y6	632.35	752.39	77.2
1649	KRT2	P35908	LALDVEIATYR	+2y5	632.35	623.35	77.2
1650	KRT2	P35908	LALDVEIATYR	+2y4	632.35	510.27	77.2
1651	KRT2	P35908	GSSSGGGYSSGSSSYGSGGR	+2y12	870.86	1088.46	94.6
1652	KRT2	P35908	GSSSGGGYSSGSSSYGSGGR	+2y11	870.86	1001.43	94.6
1653	KRT2	P35908	GSSSGGGYSSGSSSYGSGGR	+2y10	870.86	914.40	94.6
1654	KRT2	P35908	GSSSGGGYSSGSSSYGSGGR	+2y8	870.86	770.34	94.6
1655	KRT2	P35908	GSSSGGGYSSGSSSYGSGGR	+2y5	870.86	433.22	94.6
1656	KRT2	P35908	GGSSSGGGYGSGGGGSSSVK	+2y15	794.85	1213.54	89.1
1657	KRT2	P35908	GGSSSGGGYGSGGGGSSSVK	+2y14	794.85	1156.52	89.1
1658	KRT2	P35908	GGSSSGGGYGSGGGGSSSVK	+2y12	794.85	1042.48	89.1
1659	KRT2	P35908	GGSSSGGGYGSGGGGSSSVK	+2y11	794.85	879.42	89.1
1660	KRT2	P35908	GGSSSGGGYGSGGGGSSSVK	+2y9	794.85	735.36	89.1
1661	KRT2	P35908	GSSGEAFGSSVTFSFR	+2y10	811.88	1134.56	90.3
1662	KRT2	P35908	GSSGEAFGSSVTFSFR	+2y9	811.88	987.49	90.3
1663	KRT2	P35908	GSSGEAFGSSVTFSFR	+2y8	811.88	930.47	90.3
1664	KRT2	P35908	GSSGEAFGSSVTFSFR	+2y5	811.88	657.34	90.3
1665	KRT2	P35908	GSSGEAFGSSVTFSFR	+2y3	811.88	409.22	90.3
1666	LUM	P51884	SLEYLDLSFNQIAR	+2y9	834.93	1063.55	92
1667	LUM	P51884	SLEYLDLSFNQIAR	+2y8	834.93	948.53	92
1668	LUM	P51884	SLEYLDLSFNQIAR	+2y7	834.93	835.44	92
1669	LUM	P51884	SLEYLDLSFNQIAR	+2y6	834.93	748.41	92
1670	LUM	P51884	SLEYLDLSFNQIAR	+2y5	834.93	601.34	92
1671	LUM	P51884	ISNIPDEYFK	+2y9	613.31	1112.53	75.8
1672	LUM	P51884	ISNIPDEYFK	+2y8	613.31	1025.49	75.8
1673	LUM	P51884	ISNIPDEYFK	+2y6	613.31	798.37	75.8
1674	LUM	P51884	ISNIPDEYFK	+2y4	613.31	586.29	75.8
1675	LUM	P51884	ISNIPDEYFK	+2y3	613.31	457.24	75.8
1676	LUM	P51884	FNALQYLR	+2y7	512.78	877.49	68.5
1677	LUM	P51884	FNALQYLR	+2y6	512.78	763.45	68.5
1678	LUM	P51884	FNALQYLR	+2y5	512.78	692.41	68.5
1679	LUM	P51884	FNALQYLR	+2y4	512.78	579.32	68.5
1680	LUM	P51884	FNALQYLR	+2y3	512.78	451.27	68.5
1681	LUM	P51884	ILGPLSYSK	+2y8	489.29	864.48	66.8
1682	LUM	P51884	ILGPLSYSK	+2y7	489.29	751.40	66.8
1683	LUM	P51884	ILGPLSYSK	+2y6	489.29	694.38	66.8
1684	LUM	P51884	ILGPLSYSK	+2y4	489.29	484.24	66.8
1685	LUM	P51884	ILGPLSYSK	+2y3	489.29	397.21	66.8
1686	LUM	P51884	VANEVTLN	+2y6	430.23	689.35	62.5
1687	LUM	P51884	VANEVTLN	+2y5	430.23	575.30	62.5

1688	LUM	P51884	VANEVTLN	+2y4	430.23	446.26	62.5
1689	LUM	P51884	VANEVTLN	+2y3	430.23	347.19	62.5
1690	NPC2	G3V2V8	TYSYLNK	+2y6	444.73	787.40	63.5
1691	NPC2	G3V2V8	TYSYLNK	+2y5	444.73	624.34	63.5
1692	NPC2	G3V2V8	TYSYLNK	+2y4	444.73	537.30	63.5
1693	NPC2	G3V2V8	TYSYLNK	+2y3	444.73	374.24	63.5
1694	IGFBP7	Q16270	TELLPGDR	+2y6	450.74	670.39	64
1695	IGFBP7	Q16270	TELLPGDR	+2y5	450.74	557.30	64
1696	IGFBP7	Q16270	TELLPGDR	+2y4	450.74	444.22	64
1697	IGFBP7	Q16270	TELLPGDR	+2y3	450.74	347.17	64
1698	IGFBP7	Q16270	DNLAIQTR	+2y6	465.75	701.43	65.1
1699	IGFBP7	Q16270	DNLAIQTR	+2y5	465.75	588.35	65.1
1700	IGFBP7	Q16270	DNLAIQTR	+2y4	465.75	517.31	65.1
1701	IGFBP7	Q16270	DNLAIQTR	+2y3	465.75	404.23	65.1
1702	SERPING1	P05155	TNLESILSYPK	+2y9	632.84	1049.59	77.3
1703	SERPING1	P05155	TNLESILSYPK	+2y8	632.84	936.50	77.3
1704	SERPING1	P05155	TNLESILSYPK	+2y7	632.84	807.46	77.3
1705	SERPING1	P05155	TNLESILSYPK	+2y5	632.84	607.34	77.3
1706	SERPING1	P05155	TNLESILSYPK	+2y4	632.84	494.26	77.3
1707	SERPING1	P05155	VLSNNSDANLELINTWVAK	+2y11	1051.05	1300.73	107.7
1708	SERPING1	P05155	VLSNNSDANLELINTWVAK	+2y9	1051.05	1073.60	107.7
1709	SERPING1	P05155	VLSNNSDANLELINTWVAK	+2y8	1051.05	944.56	107.7
1710	SERPING1	P05155	VLSNNSDANLELINTWVAK	+2y7	1051.05	831.47	107.7
1711	SERPING1	P05155	VLSNNSDANLELINTWVAK	+2y6	1051.05	718.39	107.7
1712	SERPING1	P05155	LLDSLPSDTR	+2y9	558.80	1003.51	71.9
1713	SERPING1	P05155	LLDSLPSDTR	+2y8	558.80	890.42	71.9
1714	SERPING1	P05155	LLDSLPSDTR	+2y7	558.80	775.39	71.9
1715	SERPING1	P05155	LLDSLPSDTR	+2y6	558.80	688.36	71.9
1716	SERPING1	P05155	LLDSLPSDTR	+2y5	558.80	575.28	71.9
1717	SERPING1	P05155	LVLLNAIYLSAK	+2y10	659.41	1105.66	79.2
1718	SERPING1	P05155	LVLLNAIYLSAK	+2y9	659.41	992.58	79.2
1719	SERPING1	P05155	LVLLNAIYLSAK	+2y8	659.41	879.49	79.2
1720	SERPING1	P05155	LVLLNAIYLSAK	+2y7	659.41	765.45	79.2
1721	SERPING1	P05155	LVLLNAIYLSAK	+2y5	659.41	581.33	79.2
1722	SERPING1	P05155	FQPTLLTLPR	+2y8	593.35	910.57	74.4
1723	SERPING1	P05155	FQPTLLTLPR	+2y7	593.35	813.52	74.4
1724	SERPING1	P05155	FQPTLLTLPR	+2y6	593.35	712.47	74.4
1725	SERPING1	P05155	FQPTLLTLPR	+2y5	593.35	599.39	74.4
1726	SERPING1	P05155	FQPTLLTLPR	+2y4	593.35	486.30	74.4
1727	SPARCL1	Q14515	SSSQELGLK	+2y7	474.75	774.44	65.7
1728	SPARCL1	Q14515	SSSQELGLK	+2y6	474.75	687.40	65.7
1729	SPARCL1	Q14515	SSSQELGLK	+2y5	474.75	559.34	65.7
1730	SPARCL1	Q14515	SSSQELGLK	+2y4	474.75	430.30	65.7
1731	SPARCL1	Q14515	SSSQELGLK	+2y3	474.75	317.22	65.7
1732	SPARCL1	Q14515	DQGNQEQDPNISNGEEEEEK	+2y12	1144.97	1374.60	114.6
1733	SPARCL1	Q14515	DQGNQEQDPNISNGEEEEEK	+2y9	1144.97	1050.42	114.6
1734	SPARCL1	Q14515	DQGNQEQDPNISNGEEEEEK	+2y8	1144.97	963.39	114.6
1735	SPARCL1	Q14515	DQGNQEQDPNISNGEEEEEK	+2y7	1144.97	849.35	114.6
1736	SPARCL1	Q14515	DQGNQEQDPNISNGEEEEEK	+2y5	1144.97	663.28	114.6
1737	SPARCL1	Q14515	EEDIDENLLF	+2y7	618.79	863.45	76.2

1738	SPARCL1	Q14515	EEDIDENLLF	+2y6	618.79	750.37	76.2
1739	SPARCL1	Q14515	EEDIDENLLF	+2y5	618.79	635.34	76.2
1740	SPARCL1	Q14515	EEDIDENLLF	+2y4	618.79	506.30	76.2
1741	SPARCL1	Q14515	EEDIDENLLF	+2y3	618.79	392.25	76.2
1742	BTD	P43251	VDLITFDTPFAGR	+2y9	726.38	1011.49	84.1
1743	BTD	P43251	VDLITFDTPFAGR	+2y8	726.38	910.44	84.1
1744	BTD	P43251	VDLITFDTPFAGR	+2y7	726.38	763.37	84.1
1745	BTD	P43251	VDLITFDTPFAGR	+2y6	726.38	648.35	84.1
1746	BTD	P43251	VDLITFDTPFAGR	+2y5	726.38	547.30	84.1
1747	BTD	P43251	LSSGLVTAALYGR	+2y8	654.37	850.48	78.8
1748	BTD	P43251	LSSGLVTAALYGR	+2y7	654.37	751.41	78.8
1749	BTD	P43251	LSSGLVTAALYGR	+2y6	654.37	650.36	78.8
1750	BTD	P43251	LSSGLVTAALYGR	+2y5	654.37	579.32	78.8
1751	BTD	P43251	LSSGLVTAALYGR	+2y3	654.37	395.20	78.8
1752	B3GNT1	O43505	TALASGGVLDASGDYR	+2y12	776.88	1196.55	87.8
1753	B3GNT1	O43505	TALASGGVLDASGDYR	+2y8	776.88	896.41	87.8
1754	B3GNT1	O43505	TALASGGVLDASGDYR	+2y6	776.88	668.30	87.8
1755	B3GNT1	O43505	TALASGGVLDASGDYR	+2y5	776.88	597.26	87.8
1756	B3GNT1	O43505	TALASGGVLDASGDYR	+2y2	776.88	338.18	87.8
1757	B3GNT1	O43505	WEGPLSVSVFAATK	+2y12	746.40	1176.66	85.5
1758	B3GNT1	O43505	WEGPLSVSVFAATK	+2y11	746.40	1119.64	85.5
1759	B3GNT1	O43505	WEGPLSVSVFAATK	+2y9	746.40	909.50	85.5
1760	B3GNT1	O43505	WEGPLSVSVFAATK	+2y8	746.40	822.47	85.5
1761	B3GNT1	O43505	WEGPLSVSVFAATK	+2y7	746.40	723.40	85.5
1762	B3GNT1	O43505	YEAAVPDPR	+2y7	509.25	725.39	68.2
1763	B3GNT1	O43505	YEAAVPDPR	+2y6	509.25	654.36	68.2
1764	B3GNT1	O43505	YEAAVPDPR	+2y5	509.25	583.32	68.2
1765	B3GNT1	O43505	YEAAVPDPR	+2y4	509.25	484.25	68.2
1766	B3GNT1	O43505	YEAAVPDPR	+2y3	509.25	387.20	68.2
1767	B3GNT1	O43505	VPTFDER	+2y6	432.22	764.36	62.6
1768	B3GNT1	O43505	VPTFDER	+2y5	432.22	667.30	62.6
1769	B3GNT1	O43505	VPTFDER	+2y4	432.22	566.26	62.6
1770	B3GNT1	O43505	VPTFDER	+2y3	432.22	419.19	62.6
1771	B3GNT1	O43505	VPTFDER	+2y2	432.22	304.16	62.6
1772	B3GNT1	O43505	QYGFNR	+2y5	392.69	656.32	59.7
1773	B3GNT1	O43505	QYGFNR	+2y4	392.69	493.25	59.7
1774	B3GNT1	O43505	QYGFNR	+2y3	392.69	436.23	59.7
1775	PGLYRP2	Q96PD5	DGSPDVTTADIGANTPDATK	+2y14	973.45	1375.67	102.1
1776	PGLYRP2	Q96PD5	DGSPDVTTADIGANTPDATK	+2y13	973.45	1274.62	102.1
1777	PGLYRP2	Q96PD5	DGSPDVTTADIGANTPDATK	+2y11	973.45	1102.54	102.1
1778	PGLYRP2	Q96PD5	DGSPDVTTADIGANTPDATK	+2y9	973.45	874.43	102.1
1779	PGLYRP2	Q96PD5	DGSPDVTTADIGANTPDATK	+2y5	973.45	531.28	102.1
1780	PGLYRP2	Q96PD5	TFTLLDPK	+2y7	467.77	833.48	65.2
1781	PGLYRP2	Q96PD5	TFTLLDPK	+2y6	467.77	686.41	65.2
1782	PGLYRP2	Q96PD5	TFTLLDPK	+2y5	467.77	585.36	65.2
1783	PGLYRP2	Q96PD5	TFTLLDPK	+2y4	467.77	472.28	65.2
1784	PGLYRP2	Q96PD5	TFTLLDPK	+2y3	467.77	359.19	65.2
1785	PGLYRP2	Q96PD5	WGAAPYR	+2y6	410.71	634.33	61.1
1786	PGLYRP2	Q96PD5	WGAAPYR	+2y5	410.71	577.31	61.1
1787	PGLYRP2	Q96PD5	WGAAPYR	+2y4	410.71	506.27	61.1

1788	PGLYRP2	Q96PD5	WGAAPYR	+2y3	410.71	435.24	61.1
1789	IGHG1	P01857	ALPAPIEK	+2y7	419.76	767.47	61.7
1790	IGHG1	P01857	ALPAPIEK	+2y6	419.76	654.38	61.7
1791	IGHG1	P01857	ALPAPIEK	+2y5	419.76	557.33	61.7
1792	IGHG1	P01857	ALPAPIEK	+2y4	419.76	486.29	61.7
1793	IGHG1	P01857	ALPAPIEK	+2y3	419.76	389.24	61.7
1794	IGHG1	P01857	EPQVYTLPPSR	+2y8	643.84	932.52	78.1
1795	IGHG1	P01857	EPQVYTLPPSR	+2y7	643.84	833.45	78.1
1796	IGHG1	P01857	EPQVYTLPPSR	+2y6	643.84	670.39	78.1
1797	IGHG1	P01857	EPQVYTLPPSR	+2y5	643.84	569.34	78.1
1798	IGHG1	P01857	EPQVYTLPPSR	+2y4	643.84	456.26	78.1
1799	IGHG1	P01857	TTPPVLDSDGSFFLYSK	+2y12	937.46	1378.65	99.5
1800	IGHG1	P01857	TTPPVLDSDGSFFLYSK	+2y11	937.46	1265.57	99.5
1801	IGHG1	P01857	TTPPVLDSDGSFFLYSK	+2y10	937.46	1150.54	99.5
1802	IGHG1	P01857	TTPPVLDSDGSFFLYSK	+2y8	937.46	948.48	99.5
1803	IGHG1	P01857	TTPPVLDSDGSFFLYSK	+2y3	937.46	397.21	99.5
1804	CUTA	O60888	TQSSLVPALTDFVR	+2y12	767.42	1304.72	87.1
1805	CUTA	O60888	TQSSLVPALTDFVR	+2y9	767.42	1017.57	87.1
1806	CUTA	O60888	TQSSLVPALTDFVR	+2y8	767.42	918.50	87.1
1807	CUTA	O60888	TQSSLVPALTDFVR	+2y5	767.42	637.33	87.1
1808	CUTA	O60888	TQSSLVPALTDFVR	+2y3	767.42	421.26	87.1
1809	CUTA	O60888	QVTESVSDSITVLP	+2y12	737.89	1247.64	84.9
1810	CUTA	O60888	QVTESVSDSITVLP	+2y8	737.89	831.45	84.9
1811	CUTA	O60888	QVTESVSDSITVLP	+2y6	737.89	629.39	84.9
1812	CUTA	O60888	QVTESVSDSITVLP	+2y4	737.89	429.27	84.9
1813	OAF	Q86UD1	ALILGELEK	+2y7	493.30	801.47	67.1
1814	OAF	Q86UD1	ALILGELEK	+2y6	493.30	688.39	67.1
1815	OAF	Q86UD1	ALILGELEK	+2y5	493.30	575.30	67.1
1816	OAF	Q86UD1	ALILGELEK	+2y4	493.30	518.28	67.1
1817	OAF	Q86UD1	ALILGELEK	+2y3	493.30	389.24	67.1
1818	OAF	Q86UD1	FWLEQGVDSSVFEALPK	+2y13	976.49	1376.71	102.3
1819	OAF	Q86UD1	FWLEQGVDSSVFEALPK	+2y12	976.49	1248.65	102.3
1820	OAF	Q86UD1	FWLEQGVDSSVFEALPK	+2y11	976.49	1191.63	102.3
1821	OAF	Q86UD1	FWLEQGVDSSVFEALPK	+2y10	976.49	1092.56	102.3
1822	OAF	Q86UD1	FWLEQGVDSSVFEALPK	+2y6	976.49	704.40	102.3
1823	OAF	Q86UD1	ASEQAELPR	+2y7	500.76	842.44	67.6
1824	OAF	Q86UD1	ASEQAELPR	+2y6	500.76	713.39	67.6
1825	OAF	Q86UD1	ASEQAELPR	+2y5	500.76	585.34	67.6
1826	OAF	Q86UD1	ASEQAELPR	+2y4	500.76	514.30	67.6
1827	OAF	Q86UD1	ASEQAELPR	+2y3	500.76	385.26	67.6
1828	OAF	Q86UD1	SYSFDFYVPQR	+2y7	704.83	924.46	82.5
1829	OAF	Q86UD1	SYSFDFYVPQR	+2y6	704.83	809.43	82.5
1830	OAF	Q86UD1	SYSFDFYVPQR	+2y5	704.83	662.36	82.5
1831	OAF	Q86UD1	SYSFDFYVPQR	+2y4	704.83	499.30	82.5
1832	OAF	Q86UD1	SYSFDFYVPQR	+2y3	704.83	400.23	82.5
1833	SERPINI1	Q99574	QEVPLATLEPLVK	+2y11	718.92	1179.73	83.5
1834	SERPINI1	Q99574	QEVPLATLEPLVK	+2y10	718.92	1080.67	83.5
1835	SERPINI1	Q99574	QEVPLATLEPLVK	+2y8	718.92	870.53	83.5
1836	SERPINI1	Q99574	QEVPLATLEPLVK	+2y7	718.92	799.49	83.5
1837	SERPINI1	Q99574	QEVPLATLEPLVK	+2y4	718.92	456.32	83.5

1838	SERPINI1	Q99574	AQLVEEWANSVK	+2y10	687.36	1174.61	81.2
1839	SERPINI1	Q99574	AQLVEEWANSVK	+2y9	687.36	1061.53	81.2
1840	SERPINI1	Q99574	AQLVEEWANSVK	+2y8	687.36	962.46	81.2
1841	SERPINI1	Q99574	AQLVEEWANSVK	+2y7	687.36	833.42	81.2
1842	SERPINI1	Q99574	AQLVEEWANSVK	+2y6	687.36	704.37	81.2
1843	SERPINI1	Q99574	ALGITEIFIK	+2y8	552.84	920.55	71.4
1844	SERPINI1	Q99574	ALGITEIFIK	+2y7	552.84	863.52	71.4
1845	SERPINI1	Q99574	ALGITEIFIK	+2y6	552.84	750.44	71.4
1846	SERPINI1	Q99574	ALGITEIFIK	+2y5	552.84	649.39	71.4
1847	SERPINI1	Q99574	ALGITEIFIK	+2y3	552.84	407.27	71.4
1848	RTBDN	K7EKV3	SQQHHGLAADLGK	+2y10	681.35	1018.54	80.8
1849	RTBDN	K7EKV3	SQQHHGLAADLGK	+2y9	681.35	881.48	80.8
1850	RTBDN	K7EKV3	SQQHHGLAADLGK	+2y8	681.35	744.43	80.8
1851	RTBDN	K7EKV3	SQQHHGLAADLGK	+2y7	681.35	687.40	80.8
1852	RTBDN	K7EKV3	SQQHHGLAADLGK	+2y6	681.35	574.32	80.8
1853	RTBDN	K7EKV3	SQQHHGLAADLGK	+2y5	681.35	503.28	80.8
1854	FRZB	Q92765	NNYNYVIR	+2y6	528.27	827.44	69.6
1855	FRZB	Q92765	NNYNYVIR	+2y5	528.27	664.38	69.6
1856	FRZB	Q92765	NNYNYVIR	+2y4	528.27	550.33	69.6
1857	FRZB	Q92765	NNYNYVIR	+2y3	528.27	387.27	69.6
1858	FRZB	Q92765	SSLVNIPR	+2y6	443.26	711.45	63.4
1859	FRZB	Q92765	SSLVNIPR	+2y5	443.26	598.37	63.4
1860	FRZB	Q92765	SSLVNIPR	+2y4	443.26	499.30	63.4
1861	FRZB	Q92765	SSLVNIPR	+2y3	443.26	385.26	63.4
1862	FRZB	Q92765	LLLVEGSIAEK	+2y9	586.35	945.53	73.9
1863	FRZB	Q92765	LLLVEGSIAEK	+2y8	586.35	832.44	73.9
1864	FRZB	Q92765	LLLVEGSIAEK	+2y7	586.35	733.37	73.9
1865	FRZB	Q92765	LLLVEGSIAEK	+2y6	586.35	604.33	73.9
1866	FRZB	Q92765	LLLVEGSIAEK	+2y5	586.35	547.31	73.9
1867	WIF1	Q9Y5W5	QHTPSLK	+2y6	405.73	682.39	60.7
1868	WIF1	Q9Y5W5	QHTPSLK	+2y5	405.73	545.33	60.7
1869	WIF1	Q9Y5W5	QHTPSLK	+2y4	405.73	444.28	60.7
1870	WIF1	Q9Y5W5	QHTPSLK	+2y3	405.73	347.23	60.7

CE[f)]	MASCOT[g)]
35.9	X
35.9	X
35.9	X
35.9	X
35.9	X
22.2	X
22.2	X
22.2	X
46.6	O
46.6	O
46.6	O
46.6	O
46.6	O
32.1	O
32.1	O
32.1	O
32.1	O
32.1	O
27.9	X
27.9	X
27.9	X
27.9	X
27.9	X
41.7	X
41.7	X
41.7	X
41.7	X
41.7	X
50.9	X
50.9	X
50.9	X
50.9	X
50.9	X
31.5	X
31.5	X
31.5	X
31.5	X

31.5	X
20.7	X
20.7	X
20.7	X
20.7	X
20.7	X
35.3	X
35.3	X
35.3	X
35.3	X
35.3	X
49.9	X
49.9	X
49.9	X
49.9	X
49.9	X
20.8	X
20.8	X
20.8	X
20.8	X
20.8	X
25.6	X
25.6	X
25.6	X
25.6	X
25.6	X
27.4	X
27.4	X
27.4	X
27.4	X
27.4	X
19.1	X
19.1	X
19.1	X
19.1	X
19.1	X
31.8	O
31.8	O
31.8	O
31.8	O
31.8	O
20.6	O
20.6	O
20.6	O
20.6	O
20.6	O
18.7	X
18.7	X
18.7	X
26.8	X

26.8	X
26.8	X
26.8	X
26.8	X
24.9	O
24.9	O
24.9	O
24.9	O
24.9	O
40.2	X
40.2	X
40.2	X
40.2	X
40.2	X
30.4	X
30.4	X
30.4	X
30.4	X
30.4	X
23.6	O
23.6	O
23.6	O
23.6	O
23.6	O
25	O
25	O
25	O
25	O
25	O
31.4	X
31.4	X
31.4	X
31.4	X
31.4	X
35.5	O
35.5	O
35.5	O
35.5	O
35.5	O
41.5	X
41.5	X
41.5	X
41.5	X
41.5	X
48.5	X
48.5	X
48.5	X
48.5	X
48.5	X
40.4	O

40.4	O
40.4	O
40.4	O
40.4	O
57.4	X
57.4	X
57.4	X
57.4	X
57.4	X
23.5	X
23.5	X
23.5	X
23.5	X
26	X
26	X
26	X
26	X
26	X
61.2	X
61.2	X
61.2	X
61.2	X
61.2	X
50.1	X
50.1	X
50.1	X
50.1	X
50.1	X
30.4	X
30.4	X
30.4	X
30.4	X
30.4	X
42.4	X
42.4	X
42.4	X
42.4	X
42.4	X
35.9	X
35.9	X
35.9	X
35.9	X
35.9	X
32.5	X
32.5	X
32.5	X
32.5	X
32.5	X
26.8	O
26.8	O

26.8	O
26.8	O
26.8	O
23.8	O
23.8	O
23.8	O
23.8	O
23.8	O
50.2	X
50.2	X
50.2	X
50.2	X
50.2	X
57.5	X
57.5	X
57.5	X
57.5	X
57.5	X
24.3	O
24.3	O
24.3	O
24.3	O
24.3	O
33.8	O
33.8	O
33.8	O
33.8	O
33.8	O
57.1	X
57.1	X
57.1	X
57.1	X
57.1	X
38.9	X
38.9	X
38.9	X
38.9	X
38.9	X
32.6	X
32.6	X
32.6	X
32.6	X
32.6	X
20.9	X
20.9	X
20.9	X
20.9	X
20.9	X
27.6	X
27.6	X

27.6	X
27.6	X
27.6	X
49.7	X
49.7	X
49.7	X
49.7	X
49.7	X
30.6	X
30.6	X
30.6	X
30.6	X
30.6	X
24.8	X
24.8	X
24.8	X
24.8	X
24.8	X
46.3	X
46.3	X
46.3	X
46.3	X
46.3	X
28.3	X
28.3	X
28.3	X
28.3	X
28.3	X
23.5	X
23.5	X
23.5	X
23.5	X
40.3	O
40.3	O
40.3	O
40.3	O
40.3	O
29.8	O
29.8	O
29.8	O
29.8	O
29.8	O
31.4	X
31.4	X
31.4	X
31.4	X
31.4	X
20.7	X
20.7	X
20.7	X

20.7	X
24.4	X
24.4	X
24.4	X
24.4	X
24.4	X
30.1	X
30.1	X
30.1	X
30.1	X
30.1	X
34	X
34	X
34	X
34	X
34	X
40.8	X
40.8	X
40.8	X
40.8	X
40.8	X
50.5	X
50.5	X
50.5	X
50.5	X
50.5	X
17.9	X
17.9	X
17.9	X
17.9	X
50.2	X
50.2	X
50.2	X
50.2	X
40.1	X
40.1	X
40.1	X
40.1	X
40.1	X
51.5	X
51.5	X
51.5	X
51.5	X
51.5	X
29.9	O
29.9	O
29.9	O
29.9	O
29.9	O
29.9	X

29.9	X
29.9	X
29.9	X
29.9	X
28	X
28	X
28	X
28	X
28	X
46.8	X
46.8	X
46.8	X
46.8	X
46.8	X
18.4	X
18.4	X
18.4	X
18.4	X
18.4	X
49.6	X
49.6	X
49.6	X
49.6	X
49.6	X
27.9	O
27.9	O
27.9	O
27.9	O
27.9	O
22.8	X
22.8	X
22.8	X
22.8	X
44.6	X
44.6	X
44.6	X
44.6	X
44.6	X
19.5	X
19.5	X
19.5	X
19.5	X
26.9	X
26.9	X
26.9	X
26.9	X
26.9	X
57.8	X
57.8	X
57.8	X

57.8	X
57.8	X
36.5	O
36.5	O
36.5	O
36.5	O
36.5	O
21.7	O
21.7	O
21.7	O
21.7	O
21.7	O
21.7	X
21.7	X
21.7	X
21.7	X
21.7	X
23.5	O
23.5	O
23.5	O
23.5	O
23.5	O
22.6	O
22.6	O
22.6	O
22.6	O
54.1	X
54.1	X
54.1	X
54.1	X
54.1	X
24.9	O
24.9	O
24.9	O
24.9	O
24.9	O
43	O
43	O
43	O
43	O
43	O
41.9	X
41.9	X
41.9	X
41.9	X
41.9	X
58.6	X
58.6	X
58.6	X
58.6	X

58.6	X
25.2	X
25.2	X
25.2	X
25.2	X
25.2	X
20.6	X
20.6	X
20.6	X
20.6	X
36.3	X
36.3	X
36.3	X
36.3	X
30.8	X
30.8	X
30.8	X
30.8	X
30.8	X
30.7	X
30.7	X
30.7	X
30.7	X
30.7	X
40.7	X
40.7	X
40.7	X
40.7	X
40.7	X
29	X
29	X
29	X
29	X
29	X
51.9	X
51.9	X
51.9	X
51.9	X
51.9	X
37.3	O
37.3	O
37.3	O
37.3	O
37.3	O
34	X
34	X
34	X
34	X
34	X
28.1	X

28.1	X
60.3	X
60.3	X
60.3	X
60.3	X
60.3	X
23.1	X
23.1	X
23.1	X
23.1	X
25.5	X
25.5	X
25.5	X
25.5	X
25.5	X
39.1	O
39.1	O
39.1	O
39.1	O
39.1	O
25.1	O
25.1	O
25.1	O
25.1	O
40.6	X
40.6	X
40.6	X
40.6	X
40.6	X
29.7	X
29.7	X
29.7	X
29.7	X
29.7	X
53	X
53	X
53	X
53	X
53	X
44.9	X
44.9	X
44.9	X
44.9	X
44.9	X
16.4	O
16.4	O
16.4	O
16.4	O
33.4	X
33.4	X

33.4	X
33.4	X
33.4	X
48.1	X
48.1	X
48.1	X
48.1	X
48.1	X
17.4	X
17.4	X
17.4	X
17.4	X
23.3	X
23.3	X
23.3	X
23.3	X
23.3	X
32.1	X
32.1	X
32.1	X
32.1	X
32.1	X
38.8	X
38.8	X
38.8	X
38.8	X
38.8	X
21.4	X
21.4	X
21.4	X
21.4	X
21.4	X
27.9	X
27.9	X
27.9	X
27.9	X
27.9	X
18.9	X
18.9	X
18.9	X
18.9	X
39.4	X
39.4	X
39.4	X
39.4	X
39.4	X
40.4	X
40.4	X
40.4	X
40.4	X

40.4	X
18.5	X
18.5	X
18.5	X
18.5	X
22.5	X
22.5	X
22.5	X
22.5	X
28.7	X
28.7	X
28.7	X
28.7	X
28.7	X
21.2	O
21.2	O
21.2	O
21.2	O
21.2	O
42.8	X
42.8	X
42.8	X
42.8	X
42.8	X
40.9	X
40.9	X
40.9	X
40.9	X
40.9	X
34.3	X
34.3	X
34.3	X
34.3	X
34.3	X
44.3	X
44.3	X
44.3	X
44.3	X
44.3	X
35.4	X
35.4	X
35.4	X
35.4	X
35.4	X
26.8	O
26.8	O
26.8	O
26.8	O
26.8	O
52.9	O

52.9	O
52.9	O
52.9	O
52.9	O
37.7	X
37.7	X
37.7	X
37.7	X
37.7	X
40.3	X
40.3	X
40.3	X
40.3	X
40.3	X
20	O
20	O
20	O
20.7	X
20.7	X
20.7	X
20.7	X
25.1	O
25.1	O
25.1	O
25.1	O
25.1	O
28.3	X
28.3	X
28.3	X
28.3	X
28.3	X
21.4	X
21.4	X
21.4	X
21.4	X
29.9	O
29.9	O
29.9	O
29.9	O
29.9	O
29	X
29	X
29	X
29	X
29	X
49.2	O
49.2	O
49.2	O
49.2	O
49.2	O

24.4	O
24.4	O
24.4	O
24.4	O
24.4	O
22.3	X
22.3	X
22.3	X
22.3	X
26	X
26	X
26	X
26	X
26	X
35.9	X
35.9	X
35.9	X
35.9	X
35.9	X
56.4	X
56.4	X
56.4	X
56.4	X
56.4	X
28.4	X
28.4	X
28.4	X
28.4	X
28.4	X
35.3	X
35.3	X
35.3	X
35.3	X
35.3	X
34.9	X
34.9	X
34.9	X
34.9	X
34.9	X
33.5	X
33.5	X
33.5	X
33.5	X
33.5	X
25.1	O
25.1	O
25.1	O
25.1	O
25.1	O
30.9	O

30.9	O
30.9	O
30.9	O
30.9	O
65	X
65	X
65	X
65	X
65	X
41.4	X
41.4	X
41.4	X
41.4	X
41.4	X
21.1	X
21.1	X
21.1	X
21.1	X
21.1	X
35.7	X
35.7	X
35.7	X
35.7	X
35.7	X
35.8	X
35.8	X
35.8	X
35.8	X
35.8	X
25.5	X
25.5	X
25.5	X
25.5	X
25.5	X
30.9	X
30.9	X
30.9	X
30.9	X
30.9	X
33.2	X
33.2	X
33.2	X
33.2	X
33.2	X
26.8	X
26.8	X
26.8	X
26.8	X
26.8	X
23	X

23	X
23	X
21.9	X
21.9	X
21.9	X
21.9	X
21.9	X
33	X
33	X
33	X
33	X
33	X
39.1	X
39.1	X
39.1	X
39.1	X
39.1	X
30.1	X
30.1	X
30.1	X
30.1	X
30.1	X
50	X
50	X
50	X
50	X
50	X
31.1	X
31.1	X
31.1	X
31.1	X
31.1	X
30.7	X
30.7	X
30.7	X
30.7	X
30.7	X
25.8	O
25.8	O
25.8	O
25.8	O
25.8	O
40.3	X
40.3	X
40.3	X
40.3	X
40.3	X
53.9	X
53.9	X
53.9	X

53.9	X
53.9	X
39.7	O
39.7	O
39.7	O
39.7	O
39.7	O
36.7	X
36.7	X
36.7	X
36.7	X
36.7	X
41.4	X
41.4	X
41.4	X
41.4	X
41.4	X
37.9	X
37.9	X
37.9	X
37.9	X
37.9	X
40.6	X
40.6	X
40.6	X
40.6	X
40.6	X
46.1	X
46.1	X
46.1	X
46.1	X
46.1	X
21.9	X
21.9	X
21.9	X
21.9	X
46.2	X
46.2	X
46.2	X
46.2	X
46.2	X
23.7	X
23.7	X
23.7	X
23.7	X
23.7	X
33.1	X
33.1	X
33.1	X
33.1	X

33.1	X
31.1	X
31.1	X
31.1	X
31.1	X
31.1	X
25.2	X
25.2	X
52.6	X
52.6	X
52.6	X
52.6	X
52.6	X
59.4	X
59.4	X
59.4	X
59.4	X
59.4	X
41.2	X
41.2	X
41.2	X
41.2	X
41.2	X
50.3	X
50.3	X
50.3	X
50.3	X
50.3	X
50.7	O
50.7	O
50.7	O
50.7	O
50.7	O
28.8	O
28.8	O
28.8	O
28.8	O
28.8	O
50.5	X
50.5	X
50.5	X
50.5	X
50.5	X
24.1	O
24.1	O
24.1	O
24.1	O
24.1	O
44.5	X
44.5	X

44.5	X
44.5	X
44.5	X
27.5	X
27.5	X
27.5	X
27.5	X
27.5	X
35.3	X
35.3	X
35.3	X
35.3	X
35.3	X
34.5	X
34.5	X
34.5	X
34.5	X
34.5	X
38.7	X
38.7	X
38.7	X
38.7	X
38.7	X
44.1	X
44.1	X
44.1	X
44.1	X
44.1	X
25	X
25	X
25	X
25	X
25	X
24.9	O
24.9	O
24.9	O
24.9	O
24.9	O
35.9	X
35.9	X
35.9	X
35.9	X
35.9	X
26.3	X
26.3	X
26.3	X
26.3	X
26.3	X
35.5	O
35.5	O

35.5	O
35.5	O
35.5	O
32.5	X
32.5	X
32.5	X
32.5	X
32.5	X
43.8	X
43.8	X
43.8	X
43.8	X
43.8	X
49.2	O
49.2	O
49.2	O
49.2	O
49.2	O
39.8	X
39.8	X
39.8	X
39.8	X
39.8	X
37.2	X
37.2	X
37.2	X
37.2	X
37.2	X
33	X
33	X
33	X
33	X
33	X
38.4	X
38.4	X
38.4	X
38.4	X
38.4	X
28.4	X
28.4	X
28.4	X
28.4	X
28.4	X
32.4	X
32.4	X
32.4	X
32.4	X
32.4	X
34.2	X
34.2	X

34.2	X
34.2	X
34.2	X
41.2	X
41.2	X
41.2	X
41.2	X
41.2	X
34.4	X
34.4	X
34.4	X
34.4	X
34.4	X
35.9	X
35.9	X
35.9	X
35.9	X
35.9	X
57.4	X
57.4	X
57.4	X
57.4	X
57.4	X
39.8	X
39.8	X
39.8	X
39.8	X
39.8	X
24.6	X
24.6	X
24.6	X
24.6	X
40.2	X
40.2	X
40.2	X
40.2	X
40.2	X
48.7	X
48.7	X
48.7	X
48.7	X
48.7	X
24.4	X
24.4	X
24.4	X
24.4	X
32.8	X
32.8	X
32.8	X
32.8	X

32.8	X
30.7	X
30.7	X
30.7	X
30.7	X
30.7	X
30.7	X
30.7	X
30.7	X
46.9	X
46.9	X
46.9	X
46.9	X
46.9	X
31.7	X
31.7	X
31.7	X
31.7	X
31.7	X
51.9	X
51.9	X
51.9	X
51.9	X
51.9	X
28.7	X
28.7	X
28.7	X
28.7	X
28.7	X
25.2	X
25.2	X
25.2	X
25.2	X
25.2	X
35.2	X
35.2	X
35.2	X
35.2	X
35.2	X
41.1	X
41.1	X
41.1	X
41.1	X
41.1	X
50.1	X
50.1	X
50.1	X
50.1	X
50.1	X
52.3	X

52.3	X
52.3	X
52.3	X
52.3	X
39.6	X
39.6	X
39.6	X
39.6	X
39.6	X
30.5	X
30.5	X
30.5	X
30.5	X
30.5	X
40.4	X
40.4	X
40.4	X
40.4	X
40.4	X
27.2	X
27.2	X
27.2	X
27.2	X
27.2	X
24.6	X
24.6	X
24.6	X
24.6	X
24.6	X
36.5	X
36.5	X
36.5	X
36.5	X
36.5	X
18.9	X
18.9	X
18.9	X
18.9	X
29.4	O
29.4	O
29.4	O
29.4	O
29.4	O
23.4	X
23.4	X
23.4	X
23.4	X
23.4	X
24	X
24	X

24	X
24	X
24	X
21.4	X
21.4	X
21.4	X
21.4	X
21.4	X
27.2	X
27.2	X
27.2	X
27.2	X
27.2	X
21.1	X
21.1	X
21.1	X
21.1	X
36.1	X
36.1	X
36.1	X
36.1	X
36.1	X
48.1	X
48.1	X
48.1	X
48.1	X
48.1	X
37.5	X
37.5	X
37.5	X
37.5	X
37.5	X
39.7	X
39.7	X
39.7	X
39.7	X
39.7	X
22.3	X
22.3	X
22.3	X
22.3	X
22.3	X
30.4	X
30.4	X
30.4	X
30.4	X
30.4	X
21.3	X
21.3	X
21.3	X

21.3	X
48.1	X
48.1	X
48.1	X
48.1	X
48.1	X
41.6	X
41.6	X
41.6	X
41.6	X
41.6	X
44.7	X
44.7	X
44.7	X
44.7	X
44.7	X
29.9	X
29.9	X
29.9	X
29.9	X
29.9	X
21.4	X
21.4	X
21.4	X
21.4	X
56.7	X
56.7	X
56.7	X
56.7	X
56.7	X
19.8	O
19.8	O
19.8	O
19.8	O
45.1	O
45.1	O
45.1	O
45.1	O
45.1	O
21	X
21	X
21	X
21	X
38.5	X
38.5	X
38.5	X
38.5	X
38.5	X
27.5	X
27.5	X

27.5	X
27.5	X
27.5	X
23.4	X
23.4	X
23.4	X
23.4	X
23.4	X
38	X
38	X
38	X
38	X
38	X
38.3	O
38.3	O
38.3	O
38.3	O
38.3	O
38	X
38	X
38	X
38	X
38	X
23.2	X
23.2	X
23.2	X
23.2	X
33.2	X
33.2	X
33.2	X
33.2	X
33.2	X
40.4	X
40.4	X
40.4	X
40.4	X
40.4	X
20.2	X
20.2	X
20.2	X
20.2	X
53.8	X
53.8	X
53.8	X
53.8	X
53.8	X
42.3	X
42.3	X
42.3	X
42.3	X

42.3	X
34.5	X
34.5	X
34.5	X
34.5	X
34.5	X
40.9	X
40.9	X
40.9	X
40.9	X
40.9	X
37.3	O
37.3	O
37.3	O
37.3	O
37.3	O
44.1	X
44.1	X
44.1	X
44.1	X
44.1	X
48.2	X
48.2	X
48.2	X
48.2	X
48.2	X
41.8	X
41.8	X
41.8	X
41.8	X
41.8	X
36	X
36	X
36	X
36	X
36	X
20.5	X
20.5	X
20.5	X
46	X
46	X
46	X
46	X
46	X
37.8	X
37.8	X
37.8	X
37.8	X
37.8	X
26.1	O

26.1	O
26.1	O
26.1	O
26.1	O
44.7	X
44.7	X
44.7	X
44.7	X
44.7	X
32.2	X
32.2	X
32.2	X
32.2	X
32.2	X
26.9	X
26.9	X
26.9	X
26.9	X
26.9	X
33.1	X
33.1	X
33.1	X
33.1	X
33.1	X
21.3	X
21.3	X
21.3	X
21.3	X
21.3	X
22	O
22	O
22	O
22	O
32.5	X
32.5	X
32.5	X
32.5	X
32.5	X
20.2	X
20.2	X
20.2	X
32.1	O
32.1	O
32.1	O
32.1	O
32.1	O
35.1	X
35.1	X
35.1	X
35.1	X

35.1	X
27.8	X
27.8	X
27.8	X
27.8	X
27.8	X
43.7	X
43.7	X
43.7	X
43.7	X
43.7	X
30.8	X
30.8	X
30.8	X
30.8	X
30.8	X
38.2	X
38.2	X
38.2	X
38.2	X
38.2	X
47.8	X
47.8	X
47.8	X
47.8	X
47.8	X
20.2	X
20.2	X
20.2	X
20.2	X
20.2	X
47	X
47	X
47	X
47	X
47	X
52	X
52	X
52	X
52	X
52	X
28.3	O
28.3	O
28.3	O
28.3	O
28.3	O
21.8	X
21.8	X
21.8	X
21.8	X

21.8	X
14.2	X
14.2	X
14.2	X
21.1	X
21.1	X
21.1	X
21.1	X
33.7	X
33.7	X
33.7	X
33.7	X
33.7	X
21.1	X
21.1	X
21.1	X
21.1	X
21.1	X
42.7	O
42.7	O
42.7	O
42.7	O
42.7	O
36.3	O
36.3	O
36.3	O
36.3	O
36.3	O
47.4	X
47.4	X
47.4	X
47.4	X
47.4	X
28.8	X
28.8	X
28.8	X
28.8	X
28.8	X
36.8	X
36.8	X
36.8	X
36.8	X
36.8	X
24.5	X
24.5	X
24.5	X
24.5	X
24.5	X
29.8	X
29.8	X

29.8	X
29.8	X
29.8	X
58.1	X
58.1	X
58.1	X
58.1	X
58.1	X
30.7	X
30.7	X
30.7	X
30.7	X
30.7	X
30.7	X
30.7	X
30.7	X
30.7	X
30.7	X
53.1	X
53.1	X
53.1	X
53.1	X
53.1	X
25.1	X
25.1	X
25.1	X
25.1	X
25.1	X
37.3	X
37.3	X
37.3	X
37.3	X
37.3	X
61.4	X
61.4	X
61.4	X
61.4	X
61.4	X
28	O
28	O
28	O
28	O
28	O
47.2	X
47.2	X
47.2	X
47.2	X
47.2	X
26.4	X
26.4	X

26.4	X
26.4	X
26.4	X
22.4	X
22.4	X
22.4	X
22.4	X
22.4	X
43.3	X
43.3	X
43.3	X
43.3	X
43.3	X
23.8	X
23.8	X
23.8	X
23.8	X
23.8	X
23.9	O
23.9	O
23.9	O
23.9	O
23.9	O
29.1	O
29.1	O
29.1	O
29.1	O
29.1	O
31	X
31	X
31	X
31	X
31	X
24.1	X
24.1	X
24.1	X
24.1	X
24.1	X
42.4	X
42.4	X
42.4	X
42.4	X
42.4	X
30.1	X
30.1	X
30.1	X
30.1	X
30.1	X
33.4	X
33.4	X

33.4	X
33.4	X
33.4	X
37.8	X
37.8	X
37.8	X
37.8	X
37.8	X
31.8	X
31.8	X
31.8	X
31.8	X
31.8	X
45.4	X
45.4	X
45.4	X
45.4	X
45.4	X
41	X
41	X
41	X
41	X
41	X
42	X
42	X
42	X
42	X
42	X
43.3	X
43.3	X
43.3	X
43.3	X
43.3	X
30.7	X
30.7	X
30.7	X
30.7	X
30.7	X
25	X
25	X
25	X
25	X
25	X
23.6	X
23.6	X
23.6	X
23.6	X
23.6	X
20.3	X
20.3	X

20.3	X
20.3	X
21.1	X
21.1	X
21.1	X
21.1	X
21.4	X
21.4	X
21.4	X
21.4	X
22.3	X
22.3	X
22.3	X
22.3	X
31.8	X
31.8	X
31.8	X
31.8	X
31.8	X
55.6	X
55.6	X
55.6	X
55.6	X
55.6	X
27.6	X
27.6	X
27.6	X
27.6	X
27.6	X
33.3	X
33.3	X
33.3	X
33.3	X
33.3	X
29.6	X
29.6	X
29.6	X
29.6	X
29.6	X
22.8	X
22.8	X
22.8	X
22.8	X
22.8	X
61	X
61	X
61	X
61	X
61	X
31	X

31	X
31	X
31	X
31	X
37.1	X
37.1	X
37.1	X
37.1	X
37.1	X
33	X
33	X
33	X
33	X
33	X
40	X
40	X
40	X
40	X
40	X
38.3	X
38.3	X
38.3	X
38.3	X
38.3	X
24.8	X
24.8	X
24.8	X
24.8	X
24.8	X
20.4	O
20.4	O
20.4	O
20.4	O
20.4	O
18.1	O
18.1	O
18.1	O
51.2	X
51.2	X
51.2	X
51.2	X
51.2	X
22.4	X
22.4	X
22.4	X
22.4	X
22.4	X
19.1	X
19.1	X
19.1	X

19.1	X
19.7	X
19.7	X
19.7	X
19.7	X
19.7	X
32.4	X
32.4	X
32.4	X
32.4	X
32.4	X
49.2	X
49.2	X
49.2	X
49.2	X
49.2	X
39.5	X
39.5	X
39.5	X
39.5	X
39.5	X
37.8	X
37.8	X
37.8	X
37.8	X
23.9	X
23.9	X
23.9	X
23.9	X
23.9	X
51.4	X
51.4	X
51.4	X
51.4	X
51.4	X
24.3	X
24.3	X
24.3	X
24.3	X
24.3	X
35.9	X
35.9	X
35.9	X
35.9	X
35.9	X
36.7	X
36.7	X
36.7	X
36.7	X
36.7	X

34.9	X
34.9	X
34.9	X
34.9	X
34.9	X
27.2	X
27.2	X
27.2	X
27.2	X
27.2	X
34.6	X
34.6	X
34.6	X
34.6	X
34.6	X
34.6	X
25.8	X
25.8	X
25.8	X
25.8	X
21	X
21	X
21	X
21	X
29.2	X
29.2	X
29.2	X
29.2	X
29.2	X
18.9	X
18.9	X
18.9	X
18.9	X
